# Causes of death in children younger than five years in China in 2015: an updated analysis

**DOI:** 10.7189/jogh.06.020802

**Published:** 2016-12

**Authors:** Peige Song, Evropi Theodoratou, Xue Li, Li Liu, Yue Chu, Robert E. Black, Harry Campbell, Igor Rudan, Kit Yee Chan

**Affiliations:** 1 Centre for Global Health Research, Usher Institute of Population Health Sciences and Informatics, University of Edinburgh, Edinburgh, Scotland, United Kingdom; 2 The Institute for International Programs, Department of International Health, Johns Hopkins Bloomberg School of Public Health, Baltimore, MD, USA; * Joint last authorship

## Abstract

**Background:**

Substantial progress in reducing the child mortality rate has been made globally in the last two decades. However, for China, the number of children dying from preventable diseases is still very large. It is important to have regularly updated information on the distribution of causes of death (COD) in children to inform policy and research. In this study, we aim to estimate the COD spectrum in children younger than five years old from 2009 to 2015 with a focus on the year 2015 and to provide an updated COD prediction model for China.

**Methods:**

Updated data of under–five mortality rates (U5MRs) and the number of live births at national and provincial levels were obtained from United Nation's Inter–agency Group for Child Mortality Estimation (UN IGME), Institute for Health Metrics and Evaluation (IHME), and United Nations Population Division (UNPD). Then, we conducted a systematic review across four Chinese and English bibliographic databases and identified high–quality community–based longitudinal studies of COD in children younger than five years in China. We developed a number of single–cause models to predict the number of child death for main COD in different age groups at both national and provincial levels. The jackknife procedure was applied to construct the 95% Uncertainty Ranges (URs).

**Results:**

From 2009 to 2015, the under–five mortality rates have declined by 37.1%. The leading causes of death in 2015 were preterm birth complications (17.4%), birth asphyxia (15.2%), congenital abnormalities (14.1%), accidents (13.5%) and pneumonia (12.4%) for children under five years old. The COD spectrum varied substantially across Chinese provinces with different development levels. The leading cause in children under five years in the wealthier provinces (with lower U5MRs) was congenital abnormalities (up to 18.9%), while in the poorer provinces (with higher U5MRs), pneumonia was the dominant COD (up to 23.4%).

**Conclusions:**

This study updates and validates the accuracy of the findings of our previous COD study and proposes a new modelling method to predict proportions for the most common causes of child death in China. These updated COD estimates suggest that current strategies to reduce child mortality should prioritise action on neonatal deaths and target interventions against the top COD according to the local COD spectrum. Special attention should also be given to reducing differences between Chinese provinces and regions with differing development levels.

Child health is widely regarded as a public priority for every nation [1,2]. The under–five mortality rate (U5MR), which estimates the probability of dying between birth and the fifth birthday (usually expressed per 1000 live births) [3], is a useful indicator that measures not only the level of child health, but also the overall development of a society [4,5]. Since the adoption of the Millennium Development Goals (MDGs) by 189 member states of United Nations (UN) in September 2000 [6,7], substantial progress has been made towards improving global child survival [8].

China has made impressive progress in reducing child mortality in the 21st century and it could, therefore, serve as a model to many other low– and middle–income countries (LMIC) [7,9]. Despite the progress, gaps remain with inequities/disparities by socioeconomic status and geographical locations (rural vs urban). The newly launched UN’s Sustainable Development Goals (SDGs) framework calls for an end to preventable deaths of newborns and children under five years of age by 2030 [10]. For a populous and diverse country like China, this goal is still challenging: the total number of children who died before their fifth birthday is still very large [11]. In addition, there are also large disparities in child mortality rates between the rich and the poor, as well as between the urban and the rural regions of the country [12–14].

Targeted efforts are required immediately to address the growing inequalities by the year 2030 [10,15]. Information on causes of death (COD) is important for targeted health policy development and continuous monitoring of the progress in reducing child mortality [16]. However, a complete and universal coverage of civil registration and vital statistics (CRVS) system for providing detailed information on COD has not been achieved in China. Still, sample–based longitudinal registration systems, based on representative surveillance sites, have been in use for some time. They gradually became the most valuable resource for understanding national–level child mortality patterns [17,18]. Other relevant sources include surveillance systems, household surveys and censuses. They can also be used as additional information to the CRVS system to provide reasonably reliable data for the entire Chinese population [19,20]. A detailed description of the relevant systems for providing mortality data and COD information in China can be found in Table S1 in **Online Supplementary Document(Online Supplementary Document)**.

The two main sources on child death statistics in China are the National Maternal and Child Mortality Surveillance system (MCMS) and the National Maternal and Child Health Annual Reporting System (MCHARS). One of the main aims of MCMS is to monitor the COD in children under five years of age. An important property of MCMS is that it was designed to be representative for the entire nation, or for the three large regions at best, but not for individual provinces or counties. MCHARS has an advantage of covering the whole population of China with substantial density, so it could potentially provide U5MR estimates at the level of specific provinces, but it does not collect COD data in children [21].

To investigate the underlying COD in children under five years of age in China, a study conducted by Rudan, Chan et al. [22] estimated the COD distribution in children under five years in China between the years 2000 and 2008. Based on the information extracted from 206 multi–cause studies, they estimated proportions of different causes at the level of each province and nationally [22]. Since then, this study has been widely adopted as the most relevant source on the causes of child mortality in China [23,24]. However, that study was based on studies that were published between 2000 and 2008, and it now requires an update [1,24,25]. The year 2015 marks an appropriate time for updating the progress with the completion of MDG–4 on child mortality reduction.

The aim of this study was to conduct a new systematic analysis on COD in Chinese children based on all informative studies published in the Chinese and English literature and any other sources from 2009 onwards. The newly obtained information was used to update our estimate of the causes of child deaths in China in the period 2009–2015 and to advance our methods of estimation.

## METHODS

### Data sources

In this study, we used the national–level U5MR estimates from the UN's Inter–agency Group for Child Mortality Estimation (UN IGME), which are largely based on the data from MCMS, as reported in the latest “China Health and Family Planning Statistical Yearbook” (former China Health Statistics Yearbook). The yearbook is considered the most reliable source of the estimates of national–level U5MR, but for our assessment, we also require province–level U5MRs. For the purpose of this study, we used province–level U5MRs for the year 2013, published by Wang and colleagues [26]. We picked these estimates over possible alternatives, such as MCHARS source, because we conducted a plausibility test that compared all available estimates [27]. We concluded that the estimates by Wang et al. [26] were the most plausible because they made an attempt to adjust for the likely under–reporting in MCHARS data [26,28] and then estimated the provincial U5MRs for the year 2013 based on the corresponding national total numbers of child deaths.

In terms of assessing the number of live births in China for each year, previous studies on China’s child mortality often used the estimates from the National Bureau of Statistics of China (NBS) [22,29]. In recent years, however, the United Nations Population Division (UNPD) estimates of live births in China, as well as those from the UN IGME, have started to converge very closely to NBS estimates from China. For this reason, our study was based on live births estimates that were made available from the UNPD and the UN IGME's.

### Systematic review

To develop epidemiological models to predict the cause–specific proportions of deaths in children, a systematic review was conducted in 2015 according to the Preferred Reporting Items for Systematic reviews and Meta–Analyses (PRISMA) guidelines using a PRISMA checklist [30]. The following electronic databases were searched: China National Knowledge Infrastructure (CNKI), Wanfang Data, VIP Database for Chinese Technical Periodical (VIP) and PubMed. The search terms and strategies that were used are presented in Table S2 in **Online Supplementary Document)(Online Supplementary Document)**. All titles, abstracts and keywords were examined, and full texts of potentially relevant papers were obtained for final assessment.

In the assessment procedure, we used the selection procedures that the Child Health Epidemiology Reference Group (CHERG) advised, which came as a result of a decade–long work of this group of international experts on the questions of child mortality estimation [31]. Only the community–based, longitudinal, multi–cause studies that were mutually independent in terms of the population were included in this systematic review and analysis. This is because studies conducted at hospital sites tend to have poor representativeness of the surrounding general population, especially for children in poor rural areas where the access to hospitals is not universal [32,33]. Moreover, studies conducted retrospectively introduce recall bias, so we only included longitudinal, prospective studies. Single–cause studies tend to overestimate the reported cause, so we excluded studies that only reported a single cause, or that didn't give any breakdown by cause. Furthermore, we applied some additional criteria to ensure the quality of included studies. Uncertainties and disagreements in the study selection were resolved through discussions between the two reviewers and a consensus that was achieved in all cases. The detailed inclusion and exclusion criteria are listed in **Table 1**.

**Table 1:** Eligibility criteria for selection of studies in the systematic review

Inclusion criteria
1. Community–based study of the COD in children aged 0–4 years
2. Multi–cause studies
3. Studies with more than 100 observed deaths
**Exclusion criteria**
1. Multiple publications of the same data from the same study
2. Studies with no breakdown of deaths by cause or age
3. Studies with no reported numerical estimates
4. Unclear study design (prospective/retrospective) or unclear definitions
5. Retrospective studies
6. Studies where no overall U5MR was reported
7. Studies with inconsistencies between reported methods and presented results
8. Studies based on CDC death monitoring system
9. Studies with clear calculation mistakes or logical mistakes

All extracted data was stored in the final standardized data abstraction form, which included three parts:

1. Characteristics of the study: authors, publication year, study setting, population type (urban or rural), surveillance period, quality control method and frequency;
2. Mortality data: the number of live births, overall number of deaths and overall mortality rates for neonates, post–neonatal infants, 1–4 years old children and all 0–4 years old children;
3. COD data: Most eligible studies reported the COD data based on the national unified MCMS “child death report card” (see Table S3 in **Online Supplementary Document(Online Supplementary Document)**). Therefore, we included all pre–set causes in the data extraction form.

### Statistical analysis

The statistical procedures for estimating the proportional causes of child death in China for the years 2009–2015 included the following steps:

- Firstly, we acquired the information on national– and province–level U5MRs and the number of live births from UN IGME, IHME, and UNPD, to develop the “envelopes” for the total number of child deaths in China in the years 2009–2015;
- Secondly, based on studies derived from the systematic review, we developed three statistical models that predicted the proportion of deaths that occur in the neonatal (<1 month), postneonatal infant (1–12 months), 1–4 years (12–59 months) period, to create the “envelopes” for these particular age groups. Within each age group, we further developed statistical models that assigned COD to all deaths based on information from independent studies acquired through the systematic review. All the models were based on the best performing model below:
  ln (% *Criterion variable*) = α + β × (ln *U*5*MR*) + γ × (ln *U*5*MR*)^2^
  where the criterion variable was either the proportional contribution of the specific age group, or the specific COD. This model was chosen after an exhaustive pilot–testing of the performance of different models to address different COD (see Table S4 and Figure S1 in **Online Supplementary Document(Online Supplementary Document)** for more details);
- Thirdly, we estimated the COD structure in children in China for each of the years from 2009 to 2015 at both national and provincial levels. The jackknife procedure was applied to construct the 95% Uncertainty Ranges (URs). The procedures are described in detail in Table S5 in **Online Supplementary Document(Online Supplementary Document).** All statistical analyses were performed in R Studio (version 0.99.486) built on R (version 3.2.2).

## RESULTS

**Figure 1:**
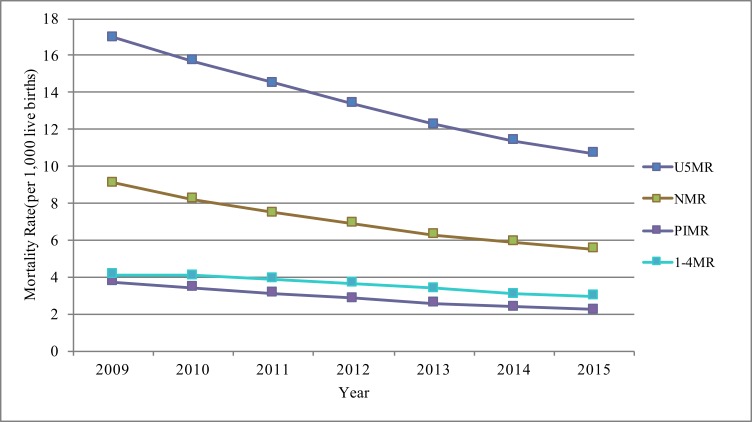
Trends in mortality rates (per 1000 live births) in China during 2009–2015 in neonates, post–neonatal infants, 1–4 year–old children and children under five years.

**Figure 2:**
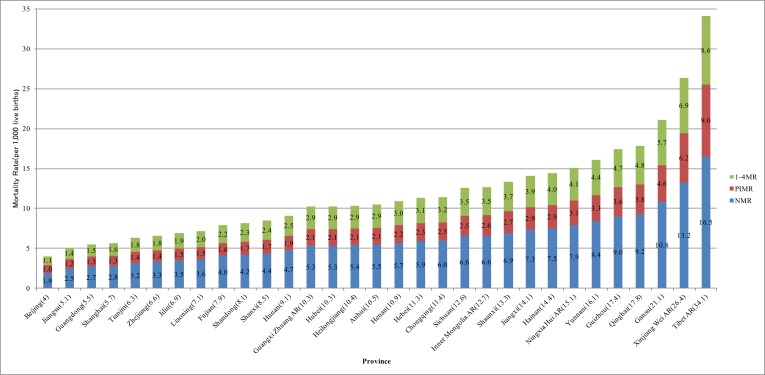
Child mortality rates in 31 provinces in China in 2015. Provinces are ranked according to under–five mortality rates (recorded in x–axis label).

**Figure 3:**
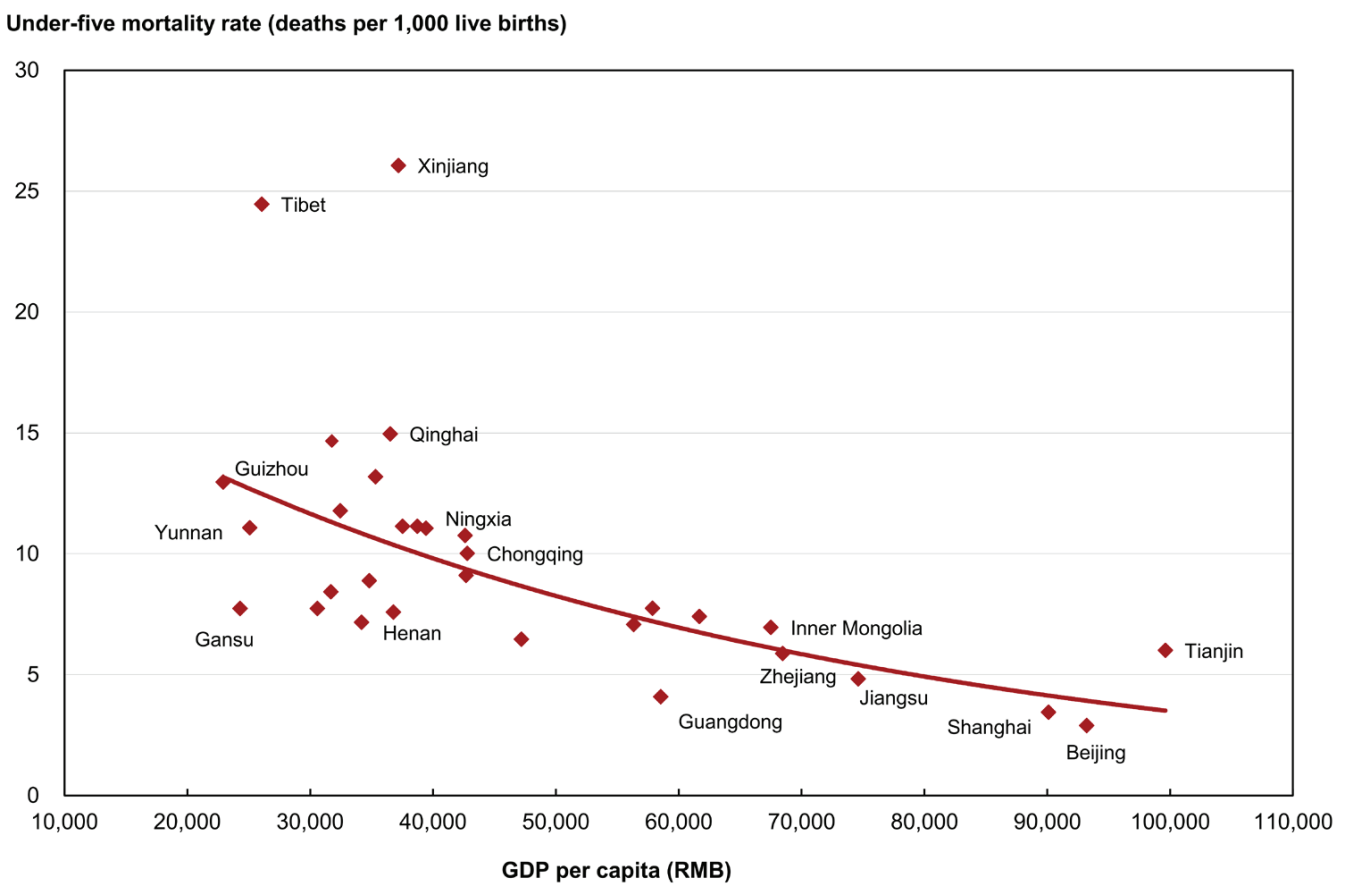
GDP per capita and under–five mortality rate in 31 provinces in 2013 (source: [35]).

According to the estimates of UN IGME [34], from 2009 to 2015, the neonatal mortality rates (NMRs), postneonatal infant mortality rates (PIMRs), 1–4 years mortality rates (1–4MRs) and U5MRs have declined by 39.6% (from 9.1 to 5.5 per 1000 live births), 40.4% (from 3.8 to 2.2 per 1000 live births), 28.5% (from 4.1 to 3.0 per 1000 live births) and 37.1% (from 17.0 to 10.7 per 1000 live births), respectively (**Figure 1**). In 2015, the provincial estimates of U5MR, NMR, PIMR and 1–4MR are shown in **Figure 2**. U5MR was the lowest in Beijing and the highest in Tibet, whereas NMR, PIMR and 1–4MR had the same geographical trend as U5MR. U5MRs in China had an inverse relationship with economic development (based on Gross Domestic Product (GDP) per capita). Provinces with higher GDP per capita had lower U5MRs, such as Beijing and Shanghai, while provinces with lower GDP per capita had higher U5MRs, such as Xinjiang and Tibet (**Figure 3**).

### Systematic review

**Figure 4:**
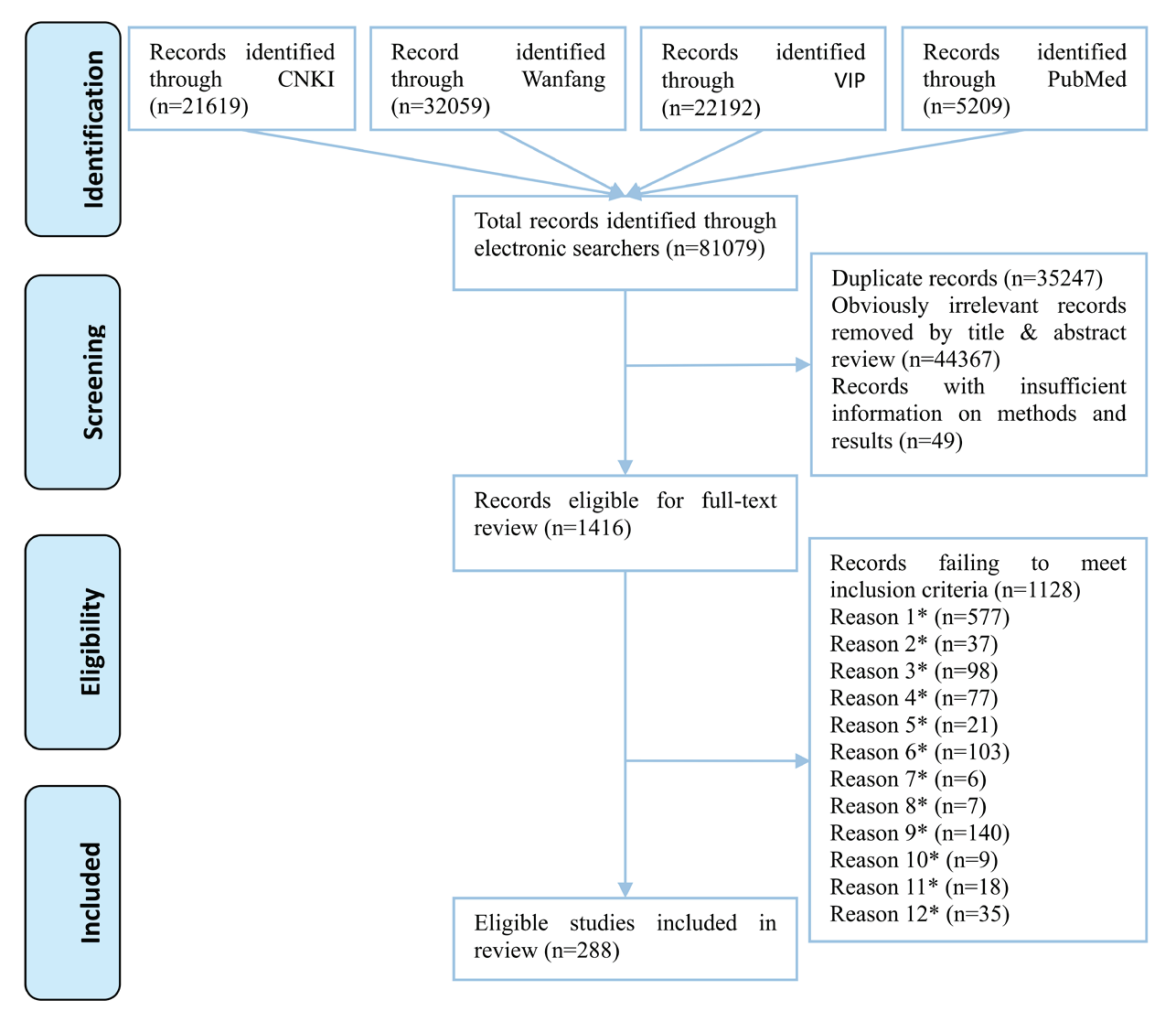
PRISMA flow diagram. Reason 1: Papers that were not community–based study of the COD in children aged 0–4 years; Reason 2: Papers that were not multi–cause studies; Reason 3: Papers with less than 100 deaths observed; Reason 4: Multiple publications of the same study; Reason 5: Papers with no breakdown of deaths by cause or age; Reason 6: Papers with no reported numerical estimates; Reason 7: Studies where design (prospective/retrospective) and definitions were not clear; Reason 8: Studies that were retrospective in design; Reason 9: Papers where no (overall) U5MR was reported for the study site; Reason 10: Papers with inconsistency between reported methods and presented results; Reason 11: Studies based on CDC death monitoring system; Reason 12: Papers with calculation mistakes or logical mistakes.

Through a literature search, a total of 81 079 citations were identified. After removing 35 247 duplicates, 44 367 apparently irrelevant citations by title and abstract review, and 49 citations with no sufficient information on methods and results, a total of 1416 articles with full–texts were reviewed to assess their eligibility. According to the study criteria, a total of 1128 publications were excluded and 288 publications were included (**Figure 4**).

**Figure 5:**
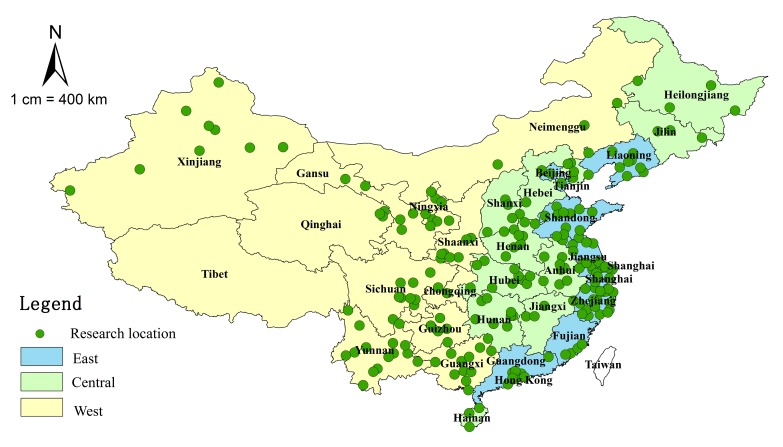
Geographic distribution of the included studies. Note: The classification of East, Central and West was based on MCMS categories according to geography and economic development of each province [36].

A summary of the main characteristics of the 288 included studies is shown in **Table 2**. The detailed information on the included studies can be found in Table S6 in **Online Supplementary Document(Online Supplementary Document)**. The geographic distribution of the 288 studies included 212 different locations in 30 provinces, municipalities and autonomous regions in China (except Tibet Autonomous Region, Hong Kong Special Administrative Region, Macao Special Administrative Region and Taiwan) (**Figure 5**).

**Table 2:** Characteristics of the included studies

Characteristics of study (Total N = 288)	Number of studies (%)
**Year published:**	
2009	51 (17.7)
2010	36 (12.5)
2011	48 (16.7)
2012	61 (21.2)
2013	47 (16.3)
2014	45 (15.6)
**Setting:**	
Urban	45 (15.6)
Rural	17 (5.9)
Both	226 (78.5)
**Number of observed deaths:**	
101–500	146 (50.7)
501–1000	56 (19.4)
1001–2000	38 (13.2)
2001–3000	20 (6.9)
3001–4000	11 (3.8)
>4000	17 (5.9)
**Number of live births:**	
<10 000	11 (3.8)
10 001–30 000	95 (33.0)
30 001–60 000	73 (25.3)
60 001–100 000	38 (13.2)
100 001–150 000	15 (5.2)
>150 000	56 (19.4)
**Reported U5MR (per 1000 live births):**	
<5.0	15 (5.2)
5.1–10.0	113 (39.2)
10.1–15.0	84 (29.2)
15.1–20.0	35 (12.2)
>20.0	41 (14.2)
**Surveillance time (year):**	
<5	69 (24.0)
5–9	167 (58.0)
10–14	50 (17.3)
>15	2 (0.7)
**Conducting quality control:**	
Yes	219 (76.0)
Unknown	69 (24.0)

All the statistical models were based on those 288 studies. The detailed description of our models can be found in Table S7 and Figures S2–S3 in **Online Supplementary Document(Online Supplementary Document)**.

### Main causes of child deaths in China

**Figure 6:**
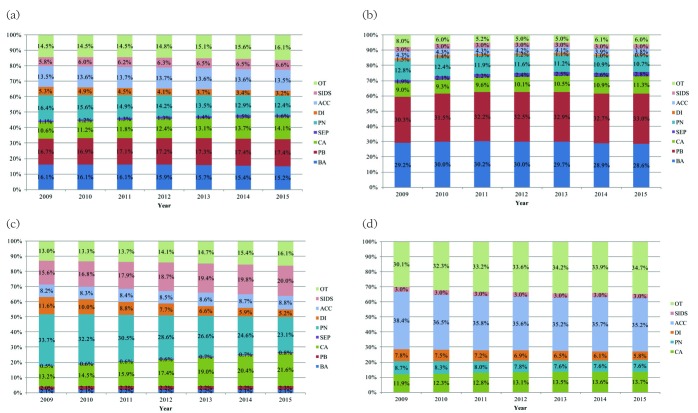
Causes of child deaths in China, 2009–2015. (a) Children under five years; (b) Neonates; (c) Post–neonatal infants; (d) 1–4 year–old children; BA – Birth asphyxia, PB – Preterm birth and low birth weight, CA – Congenital abnormalities, SEP – Neonatal sepsis, PN – Pneumonia, DI – Diarrhea, ACC – Accidents, SIDS – Sudden infant death syndrome, OT – Other.

**Figure 7:**
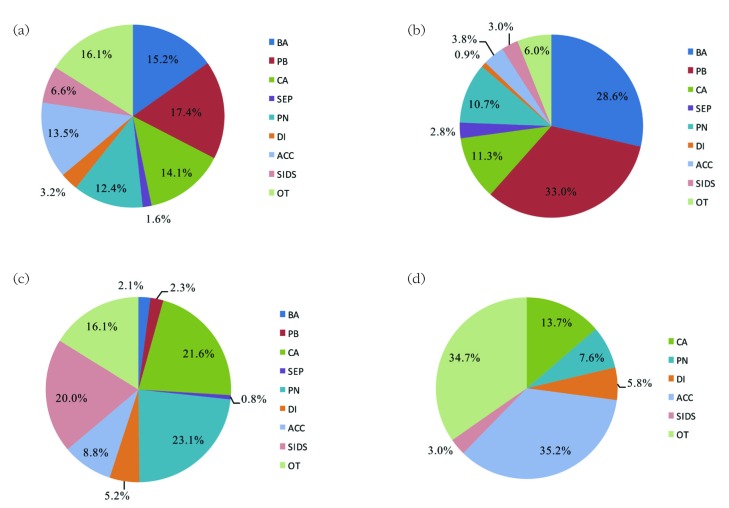
Proportional distributions of main COD in neonates, post–neonatal infants, 1–4 year–old children and children under five years in China in 2015. (a) Children under five years; (b) Neonates; (c) Post–neonatal infants; (d) 1–4 year–old children; BA – Birth asphyxia, PB – Preterm birth and low birth weight, CA – Congenital abnormalities, SEP – Neonatal sepsis, PN – Pneumonia, DI – Diarrhea, ACC – Accidents, SIDS – Sudden infant death syndrome, OT – Other.

The main COD in under five years old children are shown in **Figures 6** to **7**. In the period between 2009 and 2015, the proportions of deaths due to infectious causes – pneumonia and diarrhea – fell substantially among the children under five years of age (**Figure 6**, panel a and **Figure 7**, panel a), leading to an overall reduction of U5MR. Pneumonia decreased from 16.4% (95% UR = 16.3–16.6%) to 12.4% (95% UR = 12.3–12.5%), and diarrhea from 5.3% (95% UR = 4.9–5.5%) to 3.2% (95% UR = 2.9–3.4%). In addition, the proportion of birth asphyxia also fell slightly (from 16.1% [95% UR = 16.0–16.2%] to 15.2% [95% UR = 15.1–15.3%]), while the proportion of preterm birth / low birth weight rose slightly (from 16.7% [95% UR = 16.6–16.8%] to 17.4% [95% UR = 17.3–17.5%]). The proportion of congenital abnormalities increased much faster: from 10.6% (95% UR = 10.5–10.8%) to 14.1% (95% UR = 13.9–14.3%). The proportions of neonatal sepsis and sudden infant death syndrome (SIDS) also showed an increase in this time period (from 1.1% [95% UR = 0.9–4.7%] to 1.6% [95% UR = 1.3–2.4%], and 5.8% [95% UR = 5.5–6.2%] to 6.6% [95% UR = 6.2–6.9%], respectively). The proportion of accidents fluctuated around 13.5%, with no apparent increasing or decreasing trend. In 2015, the leading COD in children under five years of age were preterm birth / low birth weight (17.4%) and birth asphyxia (15.2%). In addition, congenital abnormalities (14.1%), accidents (13.5%) and pneumonia (12.4%) also contributed substantially.

The changes in the distribution of the main COD in neonates from 2009 to 2015 are shown in **Figure 6**, panel b. From 2009 to 2015, the proportions of deaths attributable to neonatal causes – birth asphyxia, preterm birth / low birth weight, and neonatal sepsis – accounted for more than half of all the deaths. The proportional contribution of preterm birth/low birth weight and neonatal sepsis gradually increased from 30.3% to 33.0% and 1.9% to 2.8%, respectively. The proportion of birth asphyxia declined slightly during this period – from 29.2% to 28.6%. Infectious causes – pneumonia and diarrhea – continued to decrease, from 12.8% to 10.7%, and 1.5% to 0.9%, respectively. The proportion of congenital abnormalities increased from 9.0% to 11.3%, while the proportion of accidents decreased from 4.3% to 3.8%, and the proportion of SIDS was fairly constant around 3.0%. In 2015 (**Figure 7**, panel b), preterm birth / low birth weight and birth asphyxia contributed more than half of the neonatal deaths, accounting for 33.0% and 28.6%, respectively. Congenital abnormalities (11.3%) and pneumonia (10.7%) were also the main causes that accounted for more than 10% of all neonatal deaths.

The changes in the distribution of the main COD in post–neonatal infants from 2009 to 2015 are shown in **Figure 6**, panel c: from 2009 to 2015, the proportions of infectious causes – pneumonia and diarrhea – declined substantially, from 33.7% to 23.1%, and from 11.6% to 5.2%, respectively. At the same time, the proportions of congenital abnormalities and SIDS increased from 13.2% to 21.6%, and 15.6% to 20.0%, respectively. The proportions of neonatal sepsis and accidents also rose slightly – from 0.5% to 0.8%, and from 8.2% to 8.8%, respectively. Other neonatal causes – birth asphyxia and preterm birth / low birth weight, remained relatively low, contributing to about 2.0% of all post–neonatal deaths during this period. In 2015 (**Figure 7**, panel c), most post–neonatal deaths were caused by pneumonia (23.1%), congenital abnormalities (21.6%) and SIDS (20.0%). Other important causes were accidents (8.8%) and diarrhea (5.2%).

The changes in the distribution of the main COD in the children 1–4 years of age from 2009 to 2015 are shown in **Figure 6**, panel d. From 2009 to 2015, the proportions of congenital abnormalities continued to increase, from 11.9% to 13.7%, while the other main causes – accidents and diarrhea – both declined, from 38.4% to 35.2%, and from 7.8% to 5.8%, respectively. The proportion of pneumonia declined from 8.7% to 7.6% and then remained at this level. The proportion of SIDS was around 3.0%. This trend of predominant reduction in the main causes resulted in the proportion of “other” causes increasing from 30.1% to 34.7%. In 2015 (**Figure 7**, panel d), accidental deaths became the dominant COD (35.2%) among children aged 1–4 years. Congenital abnormalities (13.7%), pneumonia (7.6%) and diarrhea (5.8%) also contributed substantially. Another dominant category was “other” (34.7%), accounting for more than one third of the deaths in 1–4 year–old children. We noticed that this category mainly included tumors and meningitis.

**Figure 8:**
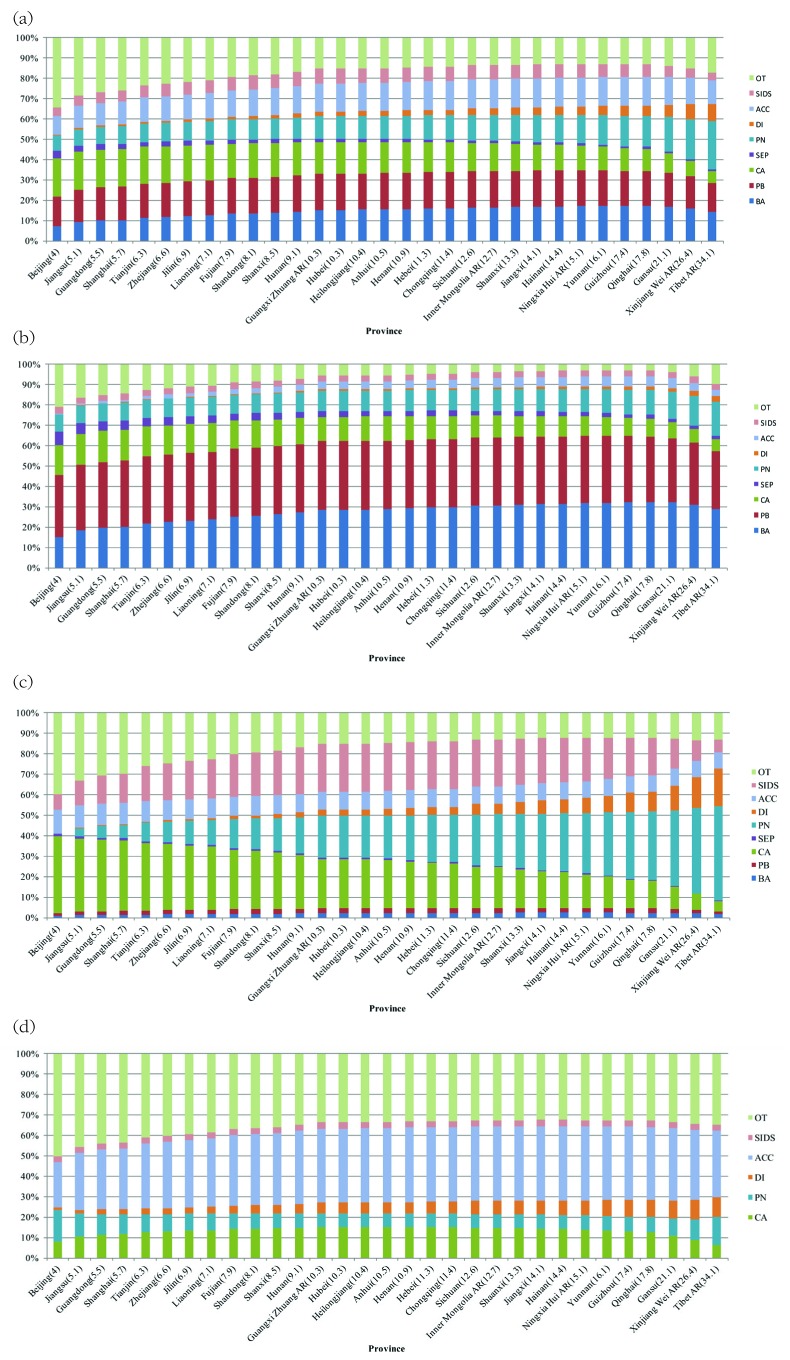
Proportional contributions of common causes of child deaths in 31 provinces in China in 2015. Provinces are ranked according to under–5 mortality rates (recorded in x–axis label); (a) Children under five years; (b) Neonates; (c) Post–neonatal infants; (d) 1–4 year–old children; BA – Birth asphyxia, PB – Preterm birth and low birth weight, CA – Congenital abnormalities, SEP – Neonatal sepsis, PN – Pneumonia, DI – Diarrhea, ACC – Accidents, SIDS – Sudden infant death syndrome, OT – Other.

The spectrum of causes of child deaths in 31 Chinese provinces (ranked by U5MRs) in 2015 is shown in **Figure 8**. In 2015, U5MR ranged from 4.0 per 1000 live births in Beijing to 34.1 per 1000 live births in Tibet. Correspondingly, the leading causes in children under five years (**Figure 8**, panel a) in the wealthier provinces (with lower U5MRs) were congenital abnormalities, preterm birth / low birth weight, and birth asphyxia, while in the poorer provinces (with higher U5MRs), the proportions of infectious diseases were still the dominant COD, especially pneumonia. Among neonates (**Figure 8**, panel b), the distributions of COD between the wealthier and the poorer provinces were quite similar, with birth asphyxia and preterm birth / low birth weight being the top two causes. The proportions of congenital abnormalities were higher in the wealthier provinces than in the poorer provinces, where pneumonia was still one of the leading causes. Among post–neonatal infants (**Figure 8**, panel c), the spectrum of causes changed dramatically with the level of development: congenital abnormalities were a dominant cause in the wealthier provinces, while pneumonia dominated in the poorer provinces. Among the children aged 1–4 years (**Figure 8**, panel d), accidents were the leading COD in every province, while the poorer provinces were still observing a large burden of child deaths from diarrhea.

## DISCUSSION

With the primary aim of this study being related to estimating proportions attributable to different COD, the spectrum of COD in children in China in the period 2009–2015 has been successfully defined in our analysis. This study was based on the most recent comprehensive review of independent studies of causes of child mortality in Mainland China and it provided an update to the previous estimates of COD in children in China in 2008 by Rudan and Chan [22]. The relationship between COD in children and overall U5MR proved to be a useful approach to predict the main COD based on overall mortality [37,38]. Through a systematic review of relevant local community–based epidemiological studies, the leading causes were successfully assessed, and they were very consistent with the previous papers in terms of cause–specific time trends between 2000 and 2015. Within the current global context of determining increasingly accurate COD estimates for children globally, our modelling–based analysis serves as an example of estimation of COD in countries where primary COD data are scarce, but the secondary data are abundant.

Preterm birth complications have been the leading COD in children under five years in China over the past seven years continually, the increasing trend of neonatal sepsis was consistent with the rise in the proportion of complications of preterm birth. This temporal change could perhaps also be explained by the rising rate of caesarean sections in China [39–41], especially of the induction/elective caesarean sections [42,43]. Nevertheless, the increase of caesarean section rate can bring overall benefits and reduce neonatal mortality, as it reduces the occurrence of birth asphyxia [44,45]. The large share of deaths that still occur in the neonatal period highlights the importance of initiating health interventions at the start of life, priorities should be set to enhancing the capacity for antenatal care and early recognition of neonatal diseases among both parents and postpartum care professionals, especially in rural poor areas.

From 2009 to 2015, the overall decrease of U5MR could have mainly been contributed to a substantial decline in deaths attributable to infectious diseases, particularly childhood pneumonia and diarrhea. According to an analysis from National Surveillance System, the decline in pneumonia deaths in China can be largely explained by a rapid economic growth, increasing access to child health care and antibiotic treatment, improvement in child nutrition (such as breastfeeding and nutrients supplementation), and health promotion [46]. These may also be important contributors to another infectious disease – diarrhea. However, our analysis confirmed that in China diarrhea was not such a common COD among children under five, in comparison to some other developing countries [47]. This may be partly explained by the common Chinese cultural practice of eating cooked food and drinking boiled water and some other hygiene practices [48]. Another feature is that infectious diseases were most relevant as the COD in post–neonatal infant period, this implies that special attention should be given to post–neonatal infants when expanding effective preventive and curative interventions among vulnerable children.

The burden of congenital abnormalities increased in relative terms during the period of 2009 to 2015, with a trend to replace birth asphyxia and become the most significant cause of child deaths in China in the future years. In 2015, congenital abnormalities have become the third most common COD in children under five years nationally and the leading COD in several economically highly developed provinces. However, for a broad cause of congenital abnormalities, primary prevention can only be effective when the understanding of causes is clear. Major factors affecting the prevalence and distribution of congenital abnormalities should be understood, with more efforts diverted to revealing the situation of congenital abnormalities in detail, to provide the basis of targeted policy.

The importance of accidents as a cause of child deaths has drawn much attention worldwide, and also in China [49,50]. Among all types of accidents, drowning has long been recognized as a very important cause [50,51]. This suggests that prevention strategies focused on child drowning should be made a national policy priority. In addition, other major accidental causes include accidental asphyxia, traffic accidents and falls. The national, and even provincial estimates of the breakdown of deaths due to accidents, can increase the universal awareness of the harm of accidents and provide the basis for policy–making on a large scale.

The relative burden of mortality due to SIDS was the worst among post–neonatal infants. The reasons for SIDS have long been regarded as unexplained, and it’s quite difficult to distinguish between SIDS and accidental asphyxia as a direct COD, especially in cases of sleep–related infant deaths. In MCMS, there’s no preset category of SIDS for estimating the actual burden. However, the high prevalence of preterm births and culturally highly prevalent practices of all–night bed sharing (particularly with newborns and infants) make it plausible that SIDS is generally classified into the preset category of accidental suffocation [52,53], which includes situations such as being smothered by a quilt, accidentally crushed by mother when she turned over, suffocated with mother's nipple in mouth, or abnormal objects in the trachea, etc. The uncertainty about coding of SIDS makes it difficult to try to understand more about this important COD.

In this study, the rigorous criterion of retaining only the “multi–cause” studies has ensured that the reported sum of COD attributable to each cause was adding up to 100% in all studies. This avoided the potential problem of the sum of all single–cause estimates adding up to more than 100% of the known number of total deaths (“envelope”), which can occur when single–cause models are primarily relied upon [23,54]. Also, a very large number of child deaths observed across 288 independent studies, which exceeded 350000, is certainly much larger than the maximum observable number of deaths within MCMS system over the same period of time. This gives our study a considerable statistical power to estimate the proportions for different COD with substantial precision, and to supplement the observations from the MCMS system. Another very important feature of this study, which provides further evidence for its strength and accuracy, is that the cause–specific estimates for the year 2009 matched very nicely the corresponding estimates for the year 2008 from the previous study [22], although the two studies were based on two entirely different and non–overlapping data sets, both of them acquired through a systematic review of Chinese literature in two different time periods: 2000–2008 and 2009–2015. A remarkable consistency between the two sets of estimates in two studies on their bordering point (2000 to 2009) leads us to a conclusion that the current study provides, in essence, a successful replication of the results presented in the previous study.

Nonetheless, there are several limitations in this study. First, for the modelling method, although the applied model was based on the best available information from high–quality studies, the model may still be biased because of unmeasured characteristics of the study population from each individual study. This is especially the case wherever the vast majority of the included studies were from the Eastern provinces, few from the Western provinces and there were hardly any studies from Tibet. In order to specify the most comprehensive and robust association between the proportions of COD and overall U5MR, a number of additional covariates should be considered, such as the local socio–economic development level, vaccination rate, etc. Second, for the purpose of defining the distribution of COD, this study only focused on a limited number of selected leading causes. This is especially true for a large category of causes such as “accidents” and “congenital abnormalities”. Third, the estimates of SIDS in this study were based on the contribution of accidental suffocation, where an over–estimation may exist and further research should be conducted to find out the real contributors to accidental suffocation. In addition, misclassification may have occurred when there was no clinical diagnosis or treatment before death, even though a detailed interview with the parents or main guardians were conducted to identify the factors attributing to the child death, the low specificity of verbal autopsies may lead to an underestimation of some specific causes, especially those relying on medical diagnosis (eg, infectious diseases, tumor, and nervous system disease) [46,55].

The updated COD estimates can serve as the basis for making child health and development–related policies, especially at a local level, where the model–based analyses are the only solution that is presently available for predicting COD in children. The model–based analyses in this study could now be readily used to conduct national and provincial estimates in all cases where MCMS primary data are not available for analysis. Even if the MCMS data are available locally, the predictive models should still have merit as a supplementary information source, especially when estimating local (provincial) distributions of COD, where MCMS data are either absent or lack representativeness. However, the estimates in this study should only be considered to represent an approximation of the true picture of the spectrum of causes of child deaths in China. Future work will be needed to externally validate the estimates presented in this study. This can be achieved by comparing our results with the primary surveillance data from MCMS, which should also help to clarify the large and uncertain “other” cause, especially for children aged 1–4 years old, where the unknown “other” has become the second dominant cause and had the tendency to become the first in the following years. The newly developed GATHER guidelines (Guidelines for Accurate and Transparent Health Estimates Reporting) should also assist in improving health estimates world–wide, and this study is one of the first examples of adoption of these guidelines [56].

Finally, despite all major advantages, development and improvement of statistical models of COD distribution should never become the ultimate focus of research on child mortality. Substantial efforts should be made to collect sufficient amount of locally informative data [24]. In China, the data presently available in MCMS are only representative nationally or regionally, but they do not allow province–level or county–level COD estimates. More attention should be focused on improving the availability and quality of CRVS, MCMS and MCHARS data resources in China and their combining in order to estimate overall and cause–specific mortality rates, which can then provide an improved picture of the causes of child deaths in China at all levels.
