# Supplementary material for: Causes of death in children younger than five years in China in 2015: an updated analysis
Source: J Glob Health. 2016 Nov 24;6(2):020802. doi: 10.7189/jogh.06.020802 (PMC5140075; doi:10.7189/jogh.06.020802)
Supplement: Online Supplementary Document [file jogh-06-020802-s001.pdf]

Online Supplementary Document

Song et al. Causes of death in children younger than five years in China in 2015: an updated analysis

J Glob Health 2016;6:020802

Table S1. Description of the sources of mortality data in China

**National Mortality Surveillance System**<br>Before 2013, the Chinese CRVS included two systems: the vital registration system of the Chinese National Health and Family Planning Commission (NHFPC) (the former Ministry of Health) and the sample-based disease surveillance points (DSP) system of the Chinese Center for Disease Control and Prevention (CDC). The vital registration system was established in 1973 and started to collect data of vital events. By 2012, this system covered around 230 million people in 22 provinces, helping to provide valuable information on both mortality and COD patterns, although the data were not truly representative for the whole China [55]. DSP was established in 1978 to collect data on individual births, deaths and 35 notifiable infectious diseases in surveillance areas [56]. By 2004, there were 161 sites included in the surveillance system, covering 73 million persons in 31 provinces. The sites were selected from different areas based on a multistage cluster sampling method, leading to a very good national representativeness of the DSP [57, 58]. From 2013, the above two systems were merged together to generate a new “National Mortality Surveillance System” (NMSS), which currently covers 605 surveillance points in 31 provinces and 24% of the whole Chinese population. The selection of surveillance points was based on a national multistage cluster sampling method, after stratifying for different socioeconomic status to ensure the representativeness [17, 58]. However, because of its high underreporting rate among children under five years (as high as 35.0 % according to under-reporting field survey) [59] and the poor performance of linking birth registration [60, 61], this system is not presently used as the official data source on child mortality [61, 62].
**National Retrospective Survey on Causes of Death**<br>Another main source of information on COD structure in China is the National Retrospective Survey on Causes of Death (NRSCD), which is also called the "Cancer Epidemiology Survey". There have been three NRSCD in China, which were conducted to collect death information for 1973-1975, 1990-1992, and 2004-2005 respectively. In these surveys, the age, sex and COD were recorded for each death [63]. The first survey was conducted at the national level between 1973-1975, covering about 850 million persons and identifying about 20 million deaths [64]. The two subsequent surveys were both sample-based surveys that used randomized cluster sampling design. The most recent, third NRSCD, covered 73 million persons in 160 counties and 53 areas with high cancer incidence [65, 66]. It retrospectively investigated all deaths reported by DSP in the study areas. The high-quality data on mortality and COD made NRSCD one of the most reliable sources on COD in China, especially on the issue of cancer prevention and control [66, 67].
**National census and inter-census surveys**<br>Reliable and complete data on population-level mortality can also be derived from direct or indirect estimates based on censuses [68]. In China, the National Bureau of Statistics (NBS) has conducted six national censuses: in 1953, 1964, 1982, 1990, 2000 and 2010. The aim was to collect accurate information on the national demographic features. The overall quality of these censuses was regarded as very high, with net under-enumeration rates of only 0.116%, 0.0014%, 0.04%, 0.06%, 1.81% and 0.12% for the years 1953, 1964, 1982, 1990, 2000 and 2010, respectively [69-71]. Since 1982, NBS regularized the census, so that it is held once in every ten years, each time in the year ending with ‘0’. During the inter-census periods, national sample surveys based on 1% of population and a stratified multi-stage sampling were also conducted every ten years, each time in the year ending with ‘5’. As these sample surveys were similar to the formal censuses, they are also referred to as “mini-censuses”. The national 1% population surveys have already been conducted in 1987, 1995, 2005 and 2015 respectively [29, 72]. In addition, National Sample Survey on Population Changes (NSSPC) was also being conducted by NBS annually from 1983 during the years when there was no census or mini-census [60], using a similar design as the censuses and mini-censuses. Based on the above censuses and surveys, NBS

publishes demographic data with a wide coverage of the whole population. The reports are published annually in the NBS statistical yearbooks. Mortality data can also be obtained from these yearbooks. However, the use of the mortality data is limited because of the lack of COD details [60].
**National Maternal and child mortality surveillance system**<br>National Maternal and Child Mortality Surveillance system (MCMS) was established in 1996 based on three independent surveillance systems, which were: (i) population-based maternal mortality surveillance system; (ii) population-based child mortality surveillance system; and (iii) hospital-based birth defect surveillance system [73, 74]. In 2007, the number of surveillance sites expanded from 116 (37 urban and 79 rural) to 336 counties/districts (126 urban and 210 rural) in 31 provinces (autonomous regions and municipalities) in Mainland China [22, 43]. Based on their geography and economic development, these sites can be further categorized into three regions: East, Central and West, with the East region being the most developed and the West region the least. The East region includes Beijing, Tianjin, Liaoning, Shanghai, Jiangsu, Zhejiang, Fujian, Shandong, and Guangdong; the Central includes Hebei, Shanxi, Jilin, Heilongjiang, Anhui, Jiangxi, Henan, Hubei, Hunan and Hainan, the West includes Inner Mongolia, Guangxi, Sichuan, Chongqing, Guizhou, Yunnan, Tibet, Shaanxi, Gansu, Qinghai, Ningxia, and Xinjiang [54].<br>A stratified cluster sampling method was used for the selection of surveillance sites to ensure the sites were distributed evenly across the 31 provinces (autonomous regions and municipalities), and that the sites are nationally and regionally representative. Data from this system can thus be used to provide national and regional estimates, but not estimates at a provincial level. The surveillance contents, case definitions, reporting methods, and quality control are unified across all surveillance sites within MCMS. The basic contents include:<br>1) The number of live births, the number of children aged 1-4 years and the number of overall population;<br>2) The number of deaths for children younger than five years of age and their corresponding COD;<br>3) The timing, locations and distribution of deaths for of children younger than five years of age;<br>4) The basic situation of health care services for children younger than five years [54, 73, 74].<br>For each community/village, one doctor is responsible for recording every newborn child, child death, or inbound/outbound migration of a child during the surveillance period. Once a death occurs, the community/village doctor is responsible for reporting it to the community health center/township hospital within ten days. Upon receiving this report, a specialist in charge of maternal and child health (MCH) organizes a home visit to verify the death within seven days. A national unified "death report card" is used to record the death related information. When a child dies at home or on the way to a hospital, a "Questionnaire of Child Death Outside of Medical Institutions" is used to conduct a verbal autopsy. The established COD is then recorded in the "Death Report Card" (see **Table S3**). When a child dies in a hospital, the "Death Report Card" would be completed based on the diagnosis from the hospital. All death causes are recorded as the primary COD and coded based on 35 causes categorized by MCMS specifically for children (see **Table S3** for the causes used for classifying child deaths). ICD-10 would be assigned automatically in the electric reporting system after the causes set by MCMS are entered in the computer system.<br>Quality control of the MCMS consists of two parts: the attention is firstly focused on a possible under-reporting of either live births or deaths, and then the focus is placed on a possible COD misclassification. For the part of quality control process relevant to under-reporting, different methods are used to conduct cross-checking, e.g. checking original records and various registrations (such as birth registration, maternal registration, registration of family planning, public security registration, vaccination cards, etc.). For COD misclassification, a team of specialists is invited to review all the reported deaths and their causes every 3, 6 or 12 months at the different levels of surveillance units, aiming to minimize the misclassification. The provincial MCMS administrative office annually checks the MCMS death list against all the child deaths recorded in NMSS, which also helps to guarantee the completeness and accuracy of the deaths and causes.
**National Maternal and Child Health Annual Reporting System**

The National Maternal and Child Health Annual Reporting System (MCHARS) was established in the beginning of 1980s. This is another registration system that specifically records the births and deaths of mothers and children. It is therefore another important source that could illuminate women and child health situation. MCHARS should theoretically cover the whole population of China. Its information is obtained from the county level in rural areas, and from the level of districts in urban areas [21, 75-77]. All data are collected based on ten report forms [54]:

1) "Maternal health annual report form";
2) "Hospital delivery monthly report form";
3) "The health situation of children under seven years old report form";
4) "Non-resident Maternal and Child Health situation annual report form";
5) "Common gynecological disease screening annual report form";
6) "Contraception operation annual report form";
7) "Intermediate induction annual report form";
8) "Family planning counseling and follow-up services annual report form";
9) "Disabled children and contraception operation complications annual report form";
10) "Pre-marital health care annual report form"

All data in the above forms are collected by community/village doctor and reported to higher level Bureaus of Health before the total number of live births from all administrative areas in one province is collated and reported to the central MCHARS office [26, 29, 77]. As a national statutory vital registration system, MCHARS collects routine information on births and maternal and child deaths in both rural and urban areas across the whole country [77]. Despite its nationwide coverage, MCHARS suffers from possible underreporting and lacks details on COD in children. For these reasons, data from MCHARS are of limited use for estimating the COD in children and MCMS should be preferred [75].

Table S2. Search strategies in CNKI, Wanfang, VIP and PubMed

Database	Access date	Subject category	Sub-database	Search terms	Publication date	Search method
**CNKI**	26/08/2015	Medicine & Public Health	Journal, Featured journal, Doctoral dissertation, Master dissertation, Domestic conferences, International conferences	(SU % 'ertong' + 'xiaoer' + 'youer' + 'yinger' + 'yingyouer' + 'xinshenger' OR TI % 'ertong' + 'xiaoer' + 'youer' + 'yinger' + 'yingyouer' + 'xinshenger' OR KY % 'ertong' + 'xiaoer' + 'youer' + 'yinger' + 'yingyouer' + 'xinshenger' OR AB % 'ertong' + 'xiaoer' + 'youer' + 'yinger' + 'yingyouer' + 'xinshenger') AND (SU % 'siwang' + 'shengcun' + 'siyin' OR TI % 'siwang' + 'shengcun' + 'siyin' OR KY % 'siwang' + 'shengcun' + 'siyin' OR AB % 'siwang' + 'shengcun' + 'siyin')	01/01/2009 - 31/12/2014	Comprehensive search: subject, title, keywords and abstract
**Wanfang**	26/08/2015	Not applicable	Journal articles, Dissertations, Conference articles	(subject: (ertong) + subject: (xiaoer) + subject: (youer) + subject: (yinger) + subject: (yingyouer) + subject: (xinshenger)) * (subject: (siwang) + subject: (shengcun) + subject: (siyin))	2009-2014	Comprehensive search: subject (including title, keywords and abstract)
**VIP**	26/08/2015	Medicine & Public Health	All journals	(M=(ertong+xiaoer+youer+yinger+yingyouer+xinshenger)+R=(ertong +xiaoer+youer+yinger+yingyouer+xinshenger))*(M=(siwang+shengcun+siyin)+R=(siwang+shengcun+siyin))	2009-2014	Comprehensive search: title, keywords and abstract
**PubMed**	06/09/2015	Not applicable	Not applicable	(((death* OR mortality or survival) AND (child* OR infant* OR neonat*) AND (China OR Chinese)))	01/01/2009 - 31/12/2014	Comprehensive search: all fields

Card No.：Health Statistics 49

Enact Authority：Ministry of Health

Approval Authority：National Bureau of Statistics

Approval No.：National Statistics [2012]184

Table S3. MCMS Child death report card

Year: 20______

_____District/County□□□□□□ □Re-made Card

No. □□□□□□□□

Address___Town(Area)__Street(Village)

Father's Name_____Mother's Name_____________

Child's Name_____ Tel_____________

Census Register：(1)Local (2)Non-local: living for less 1 year (3)Non-local: living for 1 year and above □

Gender：1.Male 2.Female 3. Sexual Ambiguity □

Birth Date

Year		Month		Day	

Birth Weight ____g (1)Measured (2)Estimated □

Gestational Age____weeks

Birth Location：

Provincial (Municipal) Hospitals

District (County) Hospitals

Street (Town) Health Centres

Village Clinics

On the Way

Home □

Death Date

Year		Month		Day	

Death Age ___Years__Months__Days___Hours

Death Diagnosis:

(a) Disease or Situation directly led to death

(b) Disease or Situation directly led to (a)

(c) Disease or Situation directly led to (b)

(d) Disease or Situation directly led to (c)

Primary Death Cause________________________________

Death Cause Code □□

ICD-10 Code □□□□

Death Location：(1) Hospitals (2) On the Way (3) Home □

Treatment Before Death：(1) In Hospital (2) Outpatient

(3) No Treatment □

Diagnostic level：(1) Provincial (Municipal)

(2) District (County)

(3) Street (Town)

(4) Village Clinics

(5) No Treatment □

Main Reason of No Treatment：(Single Selection)

(1) Financial Difficulty

(2) Traffic Inconvenience

(3) Too Late to Hospital

(4) Parents Thought Disease was not Serious

(5) Custom

(6)Other(Please Specify） □

Death Diagnosis Basis: (1) Pathologic Autopsy

(2) Clinical

(3) Estimated □

Report Institution______ Report Staff______ Report Date

**Death Cause Code**	
0l Dysentery	19 Birth asphyxia
02 Sepsis	20 Neonatal tetanus
03 Measles	2l Neonatal scleredema
04 Tuberculosis	22 Intracranial hemorrhage
05 Other infectious and parasitic diseases	23 Other neonatal diseases
06 Leukemia	24 Drowning
07 Other tumor	25 Traffic accident
08 Meningitis	26 Accidental asphyxia
09 Other neurological disease	27 Accidental poisoning
10 Pneumonia	28 Accidental fall
11 Other respiratory diseases	29 Other accidents
12 Diarrhea	30 Endocrine, nutritional and metabolic diseases
13 Other digestive diseases	31 Hematopoietic and hematopoietic organ diseases
14 Congenital heart disease	32 Circulation system disease
15 Neural tube defects	33 Urinary system disease
16 Down syndrome	34 Other
17 Other congenital abnormalities	35 Unclear diagnosis
18 Preterm or low birth weight	

Table S4. Detailed description of model testing

Model	Equation	Weighing method
**Model 1**	$\ln(\%\,Criterion\ variable) = \alpha + \beta * (\ln U5MR)$	no weighting
**Model 2**	$\ln(\%\,Criterion\ variable) = \alpha + \beta * (\ln U5MR)$	weighting proportional to the number of deaths
**Model 3**	$\ln(\%\,Criterion\ variable) = \alpha + \beta * (\ln U5MR)$	weight proportional to the square root of the number of deaths
**Model 4**	$\ln(\%\,Criterion\ variable) = \alpha + \beta * (\ln U5MR) + \gamma * (U5MR)^2$	no weighting
**Model 5**	$\ln(\%\,Criterion\ variable) = \alpha + \beta * (\ln U5MR) + \gamma * (U5MR)^2$	weighting proportional to the number of deaths
**Model 6**	$\ln(\%\,Criterion\ variable) = \alpha + \beta * (\ln U5MR) + \gamma * (U5MR)^2$	weight proportional to the square root of the number of deaths
**Model 7**	$\ln(\%\,Criterion\ variable) = \alpha + \beta * (\ln U5MR) + \gamma * (\ln U5MR)^2$	no weighting
**Model 8**	$\ln(\%\,Criterion\ variable) = \alpha + \beta * (\ln U5MR) + \gamma * (\ln U5MR)^2$	weighting proportional to the number of deaths
**Model 9**	$\ln(\%\,Criterion\ variable) = \alpha + \beta * (\ln U5MR) + \gamma * (\ln U5MR)^2$	weight proportional to the square root of the number of deaths

*Note: Where ln (U5MR) is the natural logarithm of U5MR, criterion variable refers to the proportion of each targeted age group (neonates, postneonatal infants, 1-4 years old children) or death cause.

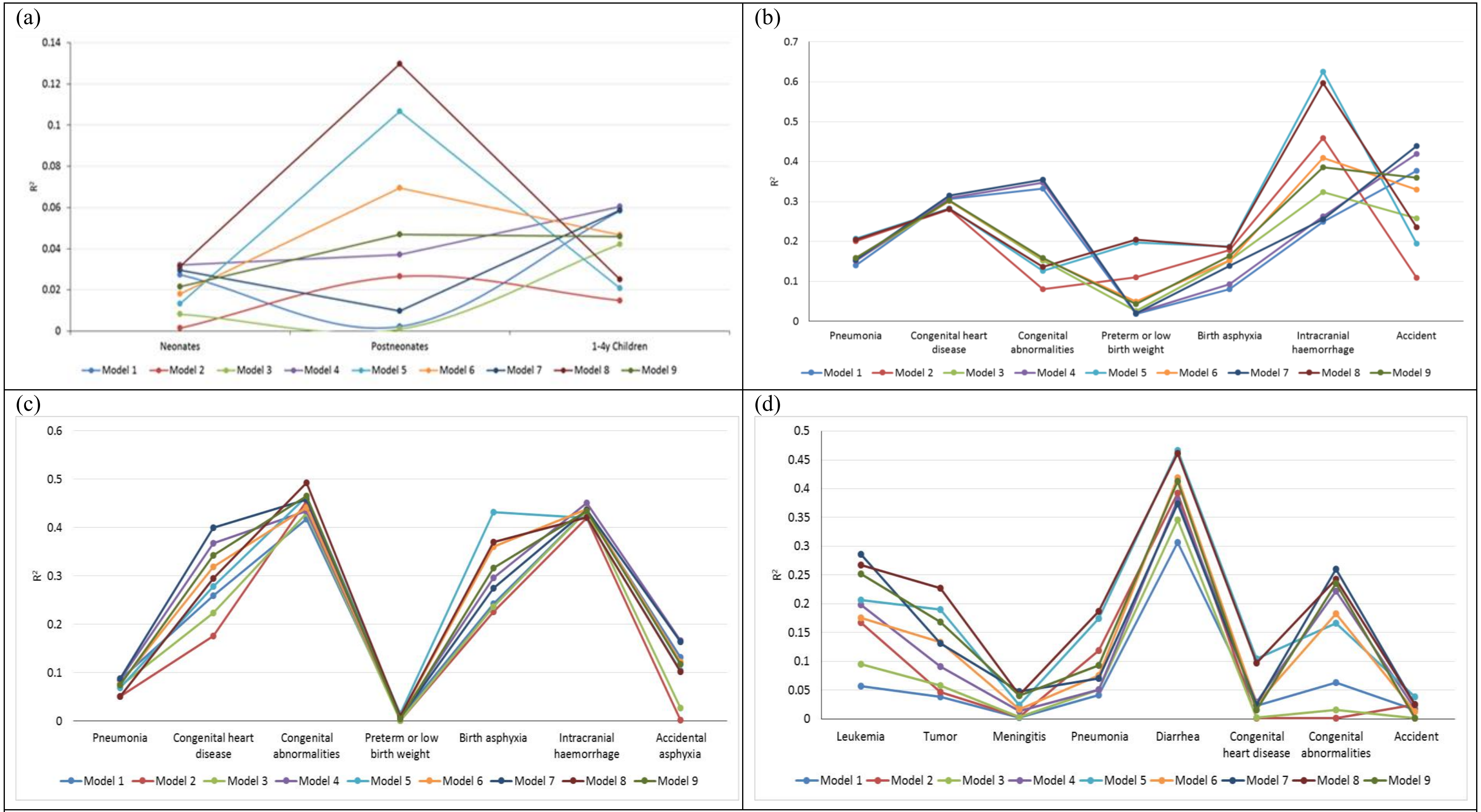

(a)
0.14
0.12
0.1
0.08
0.06
0.04
0.02
0
R²
Neonates
Postneonates
1-4y Children
Model 1 Model 2 Model 3 Model 4 Model 5 Model 6 Model 7 Model 8 Model 9
(b)
0.7
0.6
0.5
0.4
0.3
0.2
0.1
0
R²
Pneumonia
Congenital heart disease
Congenital abnormalities
Preterm or low birth weight
Birth asphyxia
Intracranial haemorrhage
Accident
Model 1 Model 2 Model 3 Model 4 Model 5 Model 6 Model 7 Model 8 Model 9
(c)
0.6
0.5
0.4
0.3
0.2
0.1
0
R²
Pneumonia
Congenital heart disease
Congenital abnormalities
Preterm or low birth weight
Birth asphyxia
Intracranial haemorrhage
Accidental asphyxia
Model 1 Model 2 Model 3 Model 4 Model 5 Model 6 Model 7 Model 8 Model 9
(d)
0.5
0.45
0.4
0.35
0.3
0.25
0.2
0.15
0.1
0.05
0
R²
Leukemia
Tumor
Meningitis
Pneumonia
Diarrhea
Congenital heart disease
Congenital abnormalities
Accident
Model 1 Model 2 Model 3 Model 4 Model 5 Model 6 Model 7 Model 8 Model 9

(e)

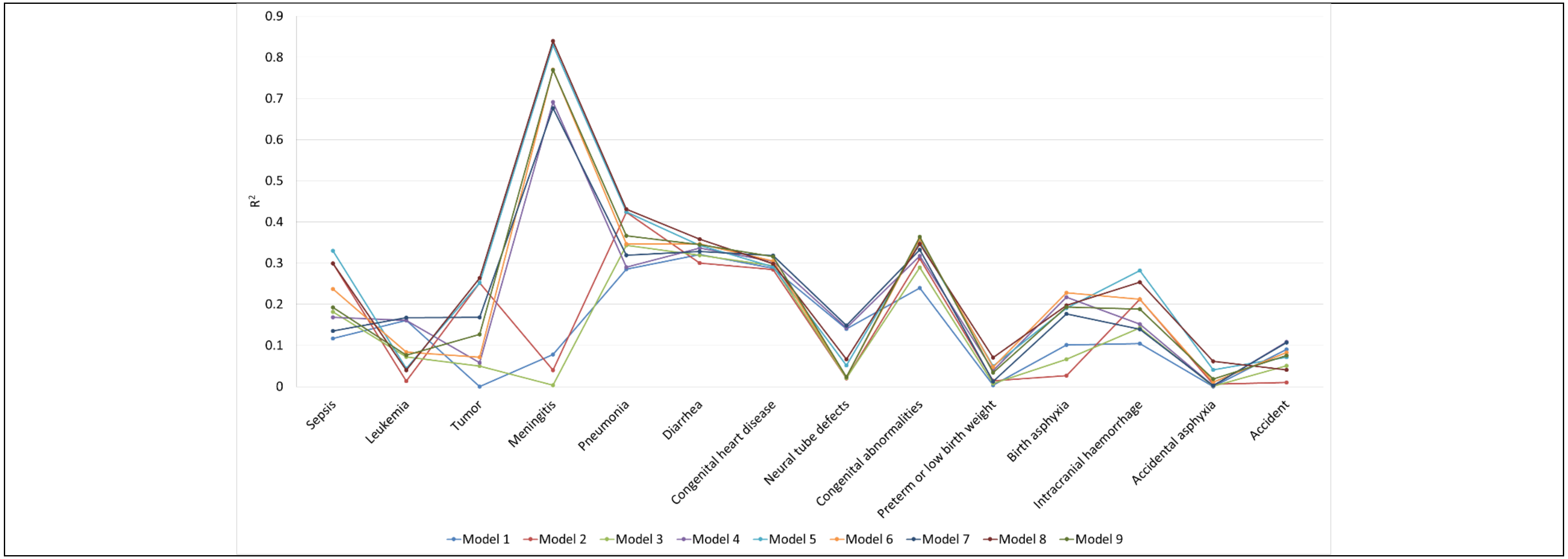

Figure S1. Comparison of the performace of the nine testing models based on R^2

*Note: The nine models were run without any a priori hypotheses about their fit to the data. All models were piloted in the same population of studies, which included all the 288 studies; (a) predicting the proportions of all 0-4 years deaths that occur in 3 separate age-groups; (b) predicting the proportions of all neonatal deaths that are due to each selected cause for neonatal period; (c) predicting the proportions of all post-neonatal infant deaths that are due to each selected cause in post-neonatal infant period; (d) predicting the proportions of deaths that occur in 1-4 years old children that are due to each selected cause in 1-4 years period; (e) predicting the proportions of all under-five deaths that are due to each selected cause for 0-4 years period.

Table S5. Statistical procedures of deriving the estimates of child death

Steps	Indicators	Methods	
		National level	Provincial level
**1**	Total number of child deaths (envelope)	Multiplying U5MR reported by MCMS by the number of live births reported by NBS and adjusted by UN IGME	Multiplying the provincial U5MRs reported by MCHARS (2010) or IHME-Chinese collaboration (2013) by the number of live births in each province reported by NBS and adjusting the total by an appropriate factor to fit the national envelope
**2**	Number of deaths in different age groups (neonates, post-neonatal infants, 1-4 years and 0-4 years) for each year in China	Modelling the proportion of all deaths in neonatal period (<1 month) and post-neonatal infant period (1-11 months) for each province using the province-level U5MR as a predictor; then, adding all the numbers of deaths and adjusting the total number of neonatal deaths by an appropriate factor to fit the national envelope for newborn deaths provided by UN IGME; and finally, computing the number of deaths of 1–4 years children (12-59 months) in each province by substracting neonatal and post-neonatal infant deaths from all 0-4 deaths in each province	Three models to estimate the age group proportions for neonates (<1 month), postneonatal infants (1–11 months), 1–4 years children (12-59 months) were developed in the systematic review. Provincial U5MRs were applied to these models to split the total number of child deaths (from Step1) into appropriate age groups at provincial level; the least informative of the three models (for 1-4 years) was dropped and the number of deaths in that age group was calculated based on estimates for other age-groups (as a remainder)
**3**	Main cause proportions for each age group	Main cause proportions were estimated for neonates (<1 month), postneonatal infants (1–11 months), 1–4 years children (12-59 months) and 0-4 years children (0-59 months) separately by applying the national U5MRs (from 2009 to 2015) to the death cause proportional models developed in the systematic review	Main cause proportions at provincial level were estimated for neonates (<1 month), postneonatal infants (1–11 months), 1–4 years children (12-59 months) and 0-4 years children (0-59 months) separately by applying the provincial U5MRs in 2015 to the death cause proportional models developed in the systematic review

Table S6. Detailed characteristics of the included studies (n=288)

Study No.	Author	Publish year	Province	SETTING	Population type	Surveillance period	Reference	Number of live births
C1	Buer BH et al.	2009	Xinjiang	Aletai autonomous prefecture	Urban and Rural	1 year (2006)	Bu-Han Buer, Yu-Mei Sun, et al. 布尔布汗,孙玉梅,等 (2009). An analysis of deaths of children under 5 years old in Aletai area in 2006* (2006 年阿勒泰地区 5 岁以下儿童死亡情况分析). Endemic Disease Bulletin (地方病通报). 24(2): 49-51	7,688
C2	Chen J et al.	2009	Anhui	1 city Anqing	Urban and Rural	3 years (2006-2008)	Jun Chen, Hong Deng, et al. 陈军,邓虹,等 (2009). Death monitoring and countermeasures of children under 5 years old in Anqing city* (安庆市 5 岁以下儿童死亡监测与干预措施). Strait J Prev Med (海峡预防医学杂志). 15(5): 24-25	161,255
C3	Chen YC et al.	2009	Hebei	1 city Chengde	Urban and Rural	7 years (01/01/2001-31/12/2007)	Yan-Chun Chen, Cui-Ping Li, et al. 陈艳春,李翠平,等 (2009). The results of death monitoring of children under 5 years old in Chengde city from 2001	278,271

							**to 2007* (2001-2007 年承德市 5 岁以下儿童死亡监测结果). Practical Preventive Medicine (实用预防医学). 16(1): 179-180**	
**C4**	**Fang M**	**2009**	**Jiangsu**	**1 district Wujin in Changzhou city**	**Urban and Rural**	**8 years (2001-2008)**	**Ming Fang 方明 (2009). Analysis and countermeasures of the results of monitoring death of children under 5 years old in Wujin district* (武进区 5 岁以下儿童死亡监测结果分析及干预措施). Jiangsu Health Care (江苏卫生保健). 11(3): 41-42**	**41,951**
**C5**	**Gu JA et al.**	**2009**	**Hebei**	**1 county Zanhuang**	**Urban and Rural**	**5 years (2004-2008)**	**Ju-Ai Gu, Zhi-Qin Wang, et al. 谷聚爱,王志芹,等 (2009). Analysis and interventions of death investigation on children under 5 years old in Zanhuang county from 2004 to 2008* (赞皇县 2004～2008 年 5 岁以下儿童死亡状况调查分析及干预措施). Clinical Misdiagnosis & Mistherapy (临床误诊误**	**20,698**

							治). 22(10): 90-91	
**C6**	**Gu YJ**	**2009**	**Jiangsu**	**7 districts and 2 counties in Wuxi city**	**Urban and Rural**	**3 years (2006-2008)**	**Yu-Jing Gu 顾宇静 (2009). An analysis of monitoring deaths of floating children under 5 years old in Wuxi city, China from 2006 to 2008* (无锡市 2006～2008 年 5 岁以下流动家庭儿童死亡监测分析). China Prac Med (中国实用医药). 4(29): 258-259**	**65,264**
**C7**	**Huang RR**	**2009**	**Guangxi**	**3 districts and 2counties in Guigang city**	**Urban and Rural**	**6 years (2002-2007)**	**Rong-Rong Huang 黄容荣 (2009). An analysis of monitoring deaths of children under 5 years old in Guigang city, China from 2002 to 2007* (2002～2007 年贵港市 5 岁以下儿童死亡监测结果分析). Maternal & Child Health Care of China (中国妇幼保健). 24(18): 2530-2531**	**403,730**
**C8**	**Huang S**	**2009**	**Zhejiang**	**14 counties, 6 streets and 2 hospitals in Yuyao city**	**Urban and Rural**	**5 years (01/10/2003-30/09/2008)**	**Su Huang 黄素 (2009). An analysis of monitoring deaths of children under 5 years old in Yuyao city, China from 2004 to 2008* (2004 年～2008 年余姚市**	**26,200**

							五岁以下儿童死亡监测分析). Chinese Journal of Birth Health & Heredity (中国优生与遗传杂志). 17(12): 117-121	
C9	Huang XL	2009	Guangdong	1 county Mei	Urban and Rural	8 years (01/10/1999-30/09/2006)	Xiao-Li Huang 黄小利 (2009). Investigation on deaths of children under 5 years old in Mei county from 1999 to 2006* (梅县 1999-2006 年 5 岁以下儿童死亡情况的调查). IMHGN (国际医药卫生导报). 15(3): 103-104	38,377
C10	Jiang CX	2009	Hubei	1 county Yingshan	Urban and Rural	5 years (2001-2005)	Chi-Xiao Jiang 姜赤晓 (2009). Analysis of death situation of children under five years old in Yingshan county during 2001-2005 (英山县 2001～2005 年五岁以下儿童死亡情况分析). Clinical Medical Engineering (临床医学工程). 16(5): 60-61	14,092
C11	Jiang H et al.	2009	Jilin	13 districts and counties in Changchun city	Urban and Rural	3 years (2006-2008)	Hua Jiang, Li-Na Ma 姜华,马丽娜 (2009). An analysis of deaths of children under 5 years old in Changchun city from	159,364

							2006 to 2008* (长春市2006-2008 年 5 岁以下儿童死亡分析). Practical Preventive Medicine (实用预防医学). 16(6): 1864-1865	
C12	Lei P	2009	Qinghai	1 city Haidong	Urban and Rural	4 years (2004-2007)	Ping Lei 雷平 (2009). An analysis of death causes of children under 5 years old in Haidong area from 2004 to 2007* (海东地区 2004 年—2007 年 5 岁以下儿童死亡原因分析). Qinghai Medical Journal (青海医药杂志). 39(3): 70-72	68,928
C13	Li GL et al.	2009	Xinjiang	1 county Yanqi	Urban and Rural	10 years (01/01/1999-30/09/2008)	Gang-Ling Li, Hui-Ping Li 李刚玲,李惠萍 (2009). An analysis of deaths of children under 5 years old in Yanqi county, Xinjiang province in last decade* (新疆巴州焉耆县 0～4 岁儿童 10 年死亡情况分析). Chinese Community Doctors (中国社区医师). 11(15): 250	12,231
C14	Li JJ et al.	2009	Hebei	17 towns in Huailai county	Urban and Rural	8 years (2000-2007)	Jing-Jing Li, Jian-Ping Guo, et al. 李晶晶,郭建平,等 (2009). An analysis of	27,766

							**monitoring deaths of children under 5 years old in Huailai county, Hebei province from 2000 to 2007* (2000～2007 年河北怀来县 5 岁以下儿童死亡监测分析). Maternal & Child Health Care of China (中国妇幼保健). 24(12): 1658-1659**	
**C15**	**Li ZM et al**	**2009**	**Yunnan**	**1 city Puer**	**Urban and Rural**	**7 years (2000-2006)**	**Zi-Mei Li, Yi Zhao 李自梅,赵一 (2009). An analysis of death monitoring result of children under 5 years old in Puer city, China from 2000 to 2006* (普洱市 2000～2006 年 5 岁以下儿童死亡监测结果分析). Maternal & Child Health Care of China (中国妇幼保健). 24(10): 1323-1324**	**124,232**
**C16**	**Lin Y**	**2009**	**Hainan**	**1 city Haikou**	**Urban and Rural**	**9 years (2000-2008)**	**Yao Lin 林尧 (2009). Monitoring analysis of children death under 5 years old from 2000 to 2008 in Haikou (海口市 2000～2008 年 5 岁以下儿童死亡监测分析). Journal of Hainan Medical**	**113,950**

							College (海南医学院学报). 15(11): 1462-1464	
C17	Liu JZ	2009	Liaoning	1 city Zhuanghe	Urban and Rural	4 years (2005-2008)	Jin-Zhuang Liu 刘金庄 (2009). Analysis and interventions of death causes of children under 5 years old in Zhuanghe city, China* (庄河市 5 岁以下儿童死亡原因分析及干预措施). China Healthcare Frontiers (中国医疗前沿). 4(21): 124-125	24,679
C18	Liu Y	2009	Hunan	5 districts and 4 counties in Changsha city	Urban and Rural	4 years (2005-2008)	Yan Liu 刘燕 (2009). An analysis of deaths of children under 5 years old in Changsha city from 2005 to 2008* (长沙市 2005-2008 年度 5 岁以下儿童死亡分析). Practical Preventive Medicine (实用预防医学). 16(6): 1863-1864	256,426
C19	liu YX et al.	2009	Guangdong	1 city Shenzhen	Urban	5 years (2003-2007)	Yi-Xin Liu, Yan Lin, et al. 刘一心,林艳,等 (2009). Analysis of under 5 years old children mortality and leading death cause in Shenzhen from 2003 to 2007 (深圳市 2003～2007	494,342

							年 5 岁以下儿童死亡监测结果分析). Morden Preventive Medicine (现代预防医学). 39(9): 1636-1638	
C20	liu YH et al.	2009	Jiangxi	1 district in Yichun city	Urban	5 years (2003-2007)	Yu-Hua Liu, Jian-Hong Yang 刘玉华,杨建红 (2009). An analysis of death causes of children under 5 years old in Yuanzhou district from 2003 to 2007* (袁州区 2003-2007 年 5 岁以下儿童死因分析). Journal of Yichun College (宜春学院学报). 31(4): 85-86	14,113
C21	Liu ZM	2009	Zhejiang	1 city Linan	Urban and Rural	10 years (1999-2008)	Zheng-Mei Liu 刘正梅 (2009). An analysis of deaths of children under 5 years old in Linan city, China from 1999 to 2008* (临安市 1999-2008 年 5 岁以下儿童死亡情况分析). Chin Prev Med (中国预防医学杂志). 10(6): 509-511	36,633
C22	Lu LM et al.	2009	Hebei	1 city Qinhuangdao	Urban and Rural	5 years (2004-2008)	Li-Min Lu, Hong-Feng Liu, et al. 卢利民,刘洪峰,等 (2009). An analysis of deaths of children under 5 years old in Qinhuangdao	157,937

							**city, China from 2004 to 2008* (秦皇岛市 2004～2008 年 5 岁以下儿童死亡结果分析). Maternal & Child Health Care of China (中国妇幼保健). 24(20): 2823-2824**	
**C23**	**Mayinuer SMT**	**2009**	**Xinjiang**	**1 county Aketao**	**Urban and Rural**	**5 years (2004-2008)**	**Sai-Mai-Ti Mayinuer 玛依努尔·赛买提 (2009). An analysis of death causes of children under 5 years old in Aketao county, China from 2004 to 2008* (阿克陶县 2004～2008 年 5 岁以下儿童死亡原因分析). China Morden Doctor (中国现代医生). 47(19): 126-128**	**17,580**
**C24**	**Peng QM et al.**	**2009**	**Jiangsu**	**1 county Binhai**	**Urban and Rural**	**5 years (2004-2008)**	**Qing-Mei Peng, Zhi Yang 彭青梅,杨智 (2009). An analysis of death causes of children under 5 years old in Binhai county from 2004 to 2008* (2004 年～2008 年滨海县 5 岁以下儿童死因分析). Jiangsu J Prev Med (江苏预防医学). 20(3): 66-67**	**70,412**
**C25**	**Shi QL**	**2009**	**Qinghai**	**1 county Pingan**	**Urban and Rural**	**5 years (2001-2005)**	**Qing-Ling Shi 石青玲 (2009). An analysis of**	**5,790**

							**death causes of children under 5 years old in Pingan county from 2001 to 2005* (平安县 2001 年～2005 年 5 岁以下儿童死因分析). Chinese Journal of Rural Medicine (中国农村医学杂志). 17(2): 60-61**	
**C26**	**Sun XY**	**2009**	**Jiangsu**	**1 county Donghai**	**Urban and Rural**	**5 years (2004-2008)**	**Xin-Yue Sun 孙新岳 (2009). An analysis and interventions of death causes of children under five years old in Donghai county from 2004 to 2008* (东海县 2004～2008 年 5 岁以下儿童死因分析及干预). Medical Information (医学信息（下旬刊）). 1(12): 277-278**	**68,143**
**C27**	**Wang DH et al.**	**2009**	**Xinjiang**	**1 city Akesu**	**Urban and Rural**	**5 years (2004-2008)**	**Dong-Hui Wang, Xi-Lian Mi, et al. 王东辉,密希连,等 (2009). Analysis and countermeasures of deaths of children under 5 years old in Akesu city from 2004 to 2008* (2004～2008 年阿克苏市 5 岁以下儿童死亡分析及对**	**30,804**

							**策). Endemic Disease Bulletin (地方病通报). 24(4): 39-40**	
**C28**	**Wang GL et al.**	**2009**	**Guangxi**	**12 counties and districts in Baise city**	**Urban and Rural**	**3 years (2005-2007)**	**Gong-Liao Wang, Mei-Xin Pan, et al. 王功僚,潘美馨,等 (2009). An analysis of monitoring results of neonatal deaths in Baise city, Guangxi province from 2005 to2007* (广西省百色市 2005 年至 2007 年新生儿死亡监测结果分析). Chin J Perinat Med (中华围产医学杂志). 12(1): 53-54**	**147,173**
**C29**	**Wang H et al.**	**2009**	**Jiangsu**	**23 towns in Qidong city**	**Rural**	**4 years (2003-2006)**	**Hua Wang, Juan-Juan Wu 王华,吴娟娟 (2009). An analysis of deaths of children under 5 years old in Qidong city, China* (启东市 5 岁以下儿童死亡分析). Maternal & Child Health Care of China (中国妇幼保健). 24(18): 2534-2536**	**22,646**
**C30**	**Wang LQ**	**2009**	**Hainan**	**1 city Danzhou**	**Urban and Rural**	**2 years (01/01/2005-31/12/2006)**	**Ling-Qin Wang 王玲勤 (2009). An analysis of deaths of children under 5 years old in Danzhou city in 2005 and 2006* (儋州市**	**19,389**

							2005 年与 2006 年 5 岁以下儿童死亡情况分析). Maternal & Child Health Care of China (中国妇幼保健). 24(24): 3382-3383	
C31	Wang PY et al.	2009	Zhejiang	1 county Jingning	Urban and Rural	8 years (01/10/1999-30/09/2007)	Pei-Ying Wang, Hai-Ju Jin 王佩英,金海菊 (2009). An analysis of deaths of children under 5 years old in Jingning county, China from 2000 to 2007* (景宁县 2000-2007 年 5 岁以下儿童死亡分析). Chinese Rural Health Service Administration (中国农村卫生事业管理). 29(3): 235-236	14,095
C32	Wang YX et al.	2009	Zhejiang	1 city Yongkang	Urban and Rural	5 years (01/01/2003-31/12/2007)	Yin-Xue Wang, Jin-Lian Huang, et al. 王银雪,黄金莲,等 (2009). An analysis of death causes of children under 5 years old in Yongkang city, China from 2003 to 2007* (永康市 2003—2007 年 5 岁以下儿童死亡原因分析). Chinese Rural Health Service Administration (中国农村卫生事业管理). 29(4): 314-316	31,919

**C33**	**Wang Z et al.**	**2009**	**Hunan**	**1 district Yongding in Zhangjiajie city**	**Urban and Rural**	**7 years (2000-2006)**	**Zhu Wang, Cai-Hong Zhao 王铸,赵彩红 (2009). An analysis of death causes of children under 5 years old in Yongding district, Zhangjiajie city from 2000 and 2006* (张家界市永定区 2000～2006 年 5 岁以下儿童死亡原因分析). Maternal & Child Health Care of China (中国妇幼保健). 24(25): 3490-3491**	**32,358**
**C34**	**Wang ZH**	**2009**	**Yunnan**	**5 towns in Changning county**	**Rural**	**5 years (01/01/2004-31/12/2008)**	**Zi-Heng Wang 王子恒 (2009). An analysis of the results of monitoring death causes of children under 5 years old in Changning county, Yunnan province from 2004 to 2008* (云南省昌宁县 2004～2008 年 5 岁以下儿童死因监测结果分析). Chin Pediatr Integr Tradit West Med (中国中西医结合儿科学). 1(6): 567-570**	**6,575**
**C35**	**Wei XJ et al.**	**2009**	**Henan**	**1 district Erqi in Zhengzhou city**	**Urban**	**3 years (01/01/2005-31/12/2007)**	**Xiu-Ju Wei, Ying-Jie Wu, et al. 魏秀菊,武英杰,等 (2009). An analysis of**	**11,749**

							**deaths of children under 5 years old in Erqi diatrict, Zhengzhou city from 2005 to 2007* (2005 至 2007 年郑州市二七区 5 岁以下儿童死亡分析). Journal of Zhengzhou University (Medical Sciences) (郑州大学学报). 44(5): 1065-1067**	
**C36**	**Wu DK**	**2009**	**Shaanxi**	**1 county Hanyin**	**Urban and Rural**	**4 years (2003-2006)**	**Deng-Kang Wu 吴登康 (2009). An analysis of deaths of children under 5 years old in Jiangyin county, China from 2003 to 2006* (汉阴县 2003～2006 年 5 岁以下儿童死亡分析). Chinese Community Doctors (中国社区医师). 11(16): 250**	**7,072**
**C37**	**Yang CL et al.**	**2009**	**Hunan**	**4 districts and 5 counties in Zhuzhou city**	**Urban and Rural**	**5 years (2004-2008)**	**Chun-Liu Yang, Xiang-Hong Chen 杨春柳,陈湘红 (2009). An analysis of neonatal death in Zhuzhou area, China from 2004 to 2008* (株洲地区 2004—2008 年新生儿死亡情况分析). Chinese Journal of Neonatology (中国新生儿科杂志).**	**208,611**

							24(6): 362-364	
C38	Yang CL et al.	2009	Hunan	1 city Zhuzhou	Urban and Rural	5 years (2004-2008)	Chun-Liu Yang, Bo Liu 杨春柳,刘波 (2009). An analysis of deaths of children under 5 years old in Zhuzhou area from 2004 to 2008* (株洲地区 2004-2008 年 5 岁以下儿童死亡情况分析). Practical Preventive Medicine (实用预防医学). 16(4): 1170-1171	209,144
C39	Yang J	2009	Yunnan	1 district Dongchuan in Kunming city	Urban	5 years (2001-2005)	Jun Yang 杨军 (2009). An analysis of neonatal deaths in Dongchuan district from 2001 to 2005* (东川区 2001 年～2005 年新生儿死亡情况分析). Soft Science of Health (卫生软科学). 23(1): 86-87	16,629
C40	Yu XH	2009	Heilongjiang	1 city Hegang	Urban and Rural	4 years (01/10/2005-30/09/2008)	Xiu-Hua Yu 于秀华 (2009). A longitudinal analysis and countermeasures of deaths of children under 5	57,392

							years old* (5 岁以下儿童死亡纵向分析及对策). World Health Digest (中外健康文摘). 8(3): 35	
C41	Zhang DJ	2009	Sichuan	1 city Langzhong	Urban and Rural	7 years (2000-2006)	De-Jun Zhang 张德军 (2009). An analysis of death causes of children under 5 years old in Langzhong city, China from 2000 and 2006* (阆中市 2000～2006 年 5 岁以下儿童死亡原因分析). Maternal & Child Health Care of China (中国妇幼保健). 24(25): 3485-3487	32,164
C42	Zhang LZ et al.	2009	Hunan	1 city Zhuzhou	Urban and Rural	2 years (2003,2007)	Li-Zhi Zhang, Jie Long, et al. 张利之,龙捷,等 (2009). An analysis of death tendency of children under 5 years old in Zhuzhou city, China from 2003 to 2007* (株洲市 2003～2007 年 5 岁以下儿童死亡趋势分析). China Morden Doctor (中国现代医生). 47(13): 56-57	78,154
C43	Zhang SL	2009	Shanxi	urban zones in Changzhi city	Urban	6 years (01/01/2001-30/09/2006)	Su-Lan Zhang 张素兰 (2009). An analysis of monitoring deaths of children under 5 years old	14,098

							in urban areas of Changzhi city from 2001 and 2006* (长治市城区 2001～2006 年 5 岁以下儿童死亡监测分析). Maternal & Child Health Care of China (中国妇幼保健). 24(10): 1451-1452	
C44	Zhang ZY et al.	2009	Zhejiang	25 towns in Songyang county	Rural	4 years (1996,2000,2003,2007)	Zhu-Yun Zhang, Ju-Hua Chen 章珠云,陈菊花 (2009). An analysis of monitoring deaths of children under 5 years old in Songyang county, China from 1996 and 2007* (松阳县 1996～2007 年 5 岁以下儿童死亡监测分析). Maternal & Child Health Care of China (中国妇幼保健). 24(9): 1232-1233	8,983
C45	Zhao JY et al.	2009	Beijing	1 district Haidian in Beijing city	Urban	4 years (2005-2008)	Jun-Ya Zhao, Jian-Yong Tong, et al. 赵俊雅,童建勇,等 (2009). An analysis of death causes of children under 5 years old in Haidian district, Beijing from 2005 to 2008* (2005～2008 年北京市海淀区 5 岁以下儿童死	44,114

							因分析). Captial Journal of Public Health (首都公共卫生). 3(6): 282-284	
C46	Zhao X	2009	Liaoning	5 districts and 2 counties in Liaoyang city	Urban and Rural	1 year (2007)	Xin Zhao 赵昕 (2009). An analysis of the death causes of children under 5 years old in Liaoyang city, Liaoning province in 2007* (辽宁省辽阳市 2007 年 5 岁以下儿童死亡原因分析). Chin Pediatr Integr Tradit West Med (中国中西医结合儿科学). 1(3): 279-280	13,526
C47	Zhu YX et al.	2009	Zhejiang	11 counties/districts/cities in Wenzhou city	Urban and Rural	1 year (01/10/2007-30/09/2008)	Ying-Xia Zhu, Jie Chen, et al. 朱映霞,陈婕,等 (2009). An analysis of deaths of children under 5 years old in Wenzhou city, China* (温州市 5 岁以下儿童死亡情况分析). Zhejiang Prev Med (浙江预防医学). 21(10): 67-68	98,892
C48	Cao WL	2010	Hunan	13 counties in Huaihua city	Urban and Rural	1 year (2008)	Wu-Lian Cao 曹务莲 (2010). Analysis and interventions of monitoring deaths of children under 5 years old in Huaihua city in 2008* (怀化市 2008 年 0～4 岁儿	17,437

							童死亡监测分析及干预措施探讨). Maternal & Child Health Care of China (中国妇幼保健). 25(7): 932-934	
C49	Zeng JW et al.	2010	Hunan	1 city Xiangtan	Urban and Rural	1 year (2009)	Jian-Wu Zeng, Yun Feng 曾建武,冯云 (2010). Current situation and outlook of deaths of children under five years old in Xiangtan city* (湘潭市 5 岁以下儿童死亡现状与思考). Medical Information (医学信息（下旬刊）). 23(11): 329-330	29,012
C50	Qiu HN	2010	Jiangsu	20 towns in Tongzhou city	Rural	5 years (2003-2007)	Hong-Nan Qiu 仇红楠 (2010). An analysis of death causes of children under 5 years old in Tongzhou city, China* (通州市 5 岁以下儿童死因分析). Maternal & Child Health Care of China (中国妇幼保健). 25(8): 1111-1112	31,625
C51	Deng FM	2010	Sichuan	1 county Rongxian	Urban and Rural	3 years (2007-2009)	Feng-Ming Deng 邓凤鸣 (2010). Rongxian 2007~2009 results of monitoring of child deaths	16,121

							(荣县 2007～2009 年儿童死亡监测结果分析). Journal of Clinical and Experimental Medicine (临床和实验医学杂志). 9(20): 1547-1548	
C52	Dou XM et al.	2010	Henan	2 districts and 5 counties in Puyang city	Urban and Rural	5 years (2003-2007)	Xiao-Min Dou, Chao Zhang 豆筱敏,张超 (2010). Analysis of the mortality in children under 5 of puyang city from 2003~2007 (濮阳市 2003～2007 年 5 岁以下儿童死亡监测结果分析). China Clin Prac Med (中国临床实用医学). 4(8): 252-253	189,102
C53	Gao JP et al.	2010	Xinjiang	1 city and 2 counties in Hami	Urban and Rural	6 years (2003-2008)	Jian-Ping Gao, Wei-Na He 高建平,贺伟娜 (2010). Tendency analysis and countermeasures of monitoring deaths of children under 5 years old in Hami area from 2003 to 2008* (2003～2008 年哈密地区 5 岁以下儿童死亡监测变化趋势及干预措施分析). Xinjiang Medical Journal (新疆医学). 40(2): 134-135	27,459

**C54**	**Guo QY et al.**	**2010**	**Yunnan**	**4 communities and 36 villages**	**Urban and Rural**	**10 years (1999-2008)**	**Qiong-Ying Guo, Yun-Fei Zhang, et al. 郭琼英,张云飞,等 (2010). An analysis of monitoring deaths of children under 5 years old in Chengjiang city from 1999 to 2008* (澂江县 1999 年～2008 年 5 岁以下儿童死亡监测分析). Soft Science of Health (卫生软科学). 24(1): 65-67**	**20,567**
**C55**	**Guo Y et al.**	**2010**	**Guangdong**	**1 district Nanhai in Foshan city**	**Urban**	**3 years (01/10/2005-30/09/2008)**	**Yan Guo, Li-Zhen Kuang, et al. 郭艳,邝丽贞,等 (2010). Analysis of mortality surveillance of children aged under 5 years during 2006~2008 in Nanhai district of Foshan city (2006 年至 2008 年佛山市南海区 5 岁以下儿童死亡监测分析). Journal of Zhengzhou University (Medical Sciences) (郑州大学学报). 45(4): 638-640**	**88,308**
**C56**	**He YN et al.**	**2010**	**Ningxia**	**1 district in Yichuan city**	**Urban**	**5 years (2004-2008)**	**Yan-Ning He, Yue-Xia Zhu, et al. 贺艳宁,朱月霞,等 (2010). An analysis of death causes of children under 5 years old in Jinfeng district from 2004**	**9,134**

							to 2008* (金凤区 2004-2008 年 5 岁以下儿童死亡原因分析). Ningxia Med J (宁夏医学杂志). 32(2): 190-191	
C57	Huang RJ	2010	Yunnan	5 towns in Mojiang county	Rural	11 years (1995-2005)	Run-Jiang Huang 黄润江 (2010). An analysis of monitoring deaths of children under 5 years old in Mojiang county, China from 1995 to 2005* (墨江县 1995～2005 年 5 岁以下儿童死亡监测结果分析). China Morden Doctor (中国现代医生). 48(4): 118-120	9,380
C58	Jia LQ	2010	Yunnan	11 counties in Menghai county	Rural	10 years (1999-2008)	Li-Qin Jia 贾丽琴 (2010). An analysis of deaths of children under 5 years old in Menghai county* (勐海县五岁以下儿童死亡情况分析). Medicine and Pharmacy of Yunnan (云南医药). 31(4): 477-479	29,164

**C59**	**Jing W et al.**	**2010**	**Xinjiang**	**1 construction corp in Wulumuqi city**	**Urban and Rural**	**5 years (2005-2009)**	**Wen Jing, Bao-Zhu Gao 敬雯, 高宝珠 (2010). To analyze the agents of the 334 death of neonatus from 2005 to 2009 in Xinjiang Formation corps (新疆生产建设兵团 2005—2009 年 334 例新生儿死亡原因分析). Chinese Primary Health Care (中国初级卫生保健). 24(9): 26-27**	**58,474**
**C60**	**Liu B et al.**	**2010**	**Liaoning**	**1 city Shenyang**	**Urban and Rural**	**4 years (01/10/2002-30/09/2007)**	**Bing Liu, Liu Yang, et al. 刘冰,杨柳,等 (2010). An analysis of deaths of floating children under 5 years old in Shenyang city, China* (沈阳市流动人口 5 岁以下儿童死亡状况调查). Maternal & Child Health Care of China (中国妇幼保健). 25(9): 1231-1233**	**19,019**
**C61**	**Liu WH**	**2010**	**Fujian**	**43 streets and towns in Zhangzhou**	**Urban and Rural**	**5 years (2003-2007)**	**Wen-Huang Liu 刘文煌 (2010). An analysis of deaths of children under 5 years old in Zhangzhou city, China from 2003 to 2007* (漳州市 2003～2007 年 5 岁以下儿童死亡**	**88,523**

							分析). Maternal & Child Health Care of China (中国妇幼保健). 25(5): 642-643	
C62	Liu XL et al.	2010	Qinghai	1 city and 9 counties	Urban and Rural	5 years (2004-2008)	Xue-Li Liu, Jin-Shou Yang 刘学莉,杨进寿 (2010). Analysis of cause of death of children under 5 years in Qinghai (青海省 5 岁以下儿童死因分析). Morden Preventive Medicine (现代预防医学). 37(19): 3637-3638	34,216
C63	Ma ML	2010	Ningxia	21 towns in Longde county	Rural	8 years (2000-2007)	Ming-Lu Ma 马明录 (2010). An analysis of deaths of children under 5 years old in Longde county, China from 2000 to 2007* (2000～2007 年隆德县 5 岁以下儿童死亡情况分析). Maternal & Child Health Care of China (中国妇幼保健). 25(4): 503-504	15,933
C64	Pu B et al.	2010	Yunnan	14 counties in Kunming city	Urban and Rural	8 years (2000-2007)	Bin Pu, Xiu-Lian Xu, et al. 蒲斌,徐秀莲,等 (2010). Analysis of mortality status of children under 5 years in Kunming from 2000 to 2007 (昆明市	338,719

							2000—2007 年 5 岁以下儿童死亡状况分析). CJCHC (中国儿童保健杂志). 18(6): 521-524	
C65	Quan JH	2010	Jilin	5 counties and cities in Yanbian autonomous prefecture	Urban and Rural	5 years (2005-2009)	Jing-Hua Quan 全京花 (2010). An analysis of death review result of children under 5 years old in Yanbian monitoring points, China from 2005 to 2009* (延边州监测点 2005—2009 年 5 岁以下儿童死亡评审结果分析). CJCHC (中国儿童保健杂志). 18(7): 624-625	11,703
C66	Shen YY	2010	Qinghai	1 county Huzhu	Urban and Rural	7 years (2000-2006)	Yu-Ying Shen 申玉英 (2010). An analysis of death causes of children under 5 years old in Huzhu county, China from 2000 to 2006* (互助县 2000～2006 年度 5 岁以下儿童死亡原因分析). Maternal & Child Health Care of China (中国妇幼保健). 25(22): 3131-3133	323,074
C67	Shi HM	2010	Gansu	all counties in Gannan state	Urban and Rural	10 years (01/2000-12/2009)	Hua-Ming Shi 石华明 (2010). Analysis of the changes of 0-5 year-old children death in Tibetan	92,800

							**Autonomous Prefecture of Gannan of Gansu province in the past 10 years (甘肃省甘南藏族自治州 0～5 岁儿童死亡近 10 年变化分析). Chinese Journal of Healthy Birth & Child Care (中国优生优育). 16(6): 291-293**	
**C68**	**Shu RX**	**2010**	**Zhejiang**	**1 county Jinyun**	**Urban and Rural**	**10 years (01/10/1999-30/09/2009)**	**Ru-Xin Shu 舒如新 (2010). An analysis of deaths of children under 5 years old in Jinyun county, China in last decade* (缙云县 10 年 5 岁以下儿童死亡分析). Chinese Rural Health Service Administration (中国农村卫生事业管理). 30(6): 485-486**	**43,347**
**C69**	**Sun LP et al.**	**2010**	**Henan**	**1 district in Hebi city**	**Urban and Rural**	**10 years (2000-2009)**	**Li-Ping Sun, Xiang-Mei Zhu 孙丽萍,祝香梅 (2010). Investigation on death results of children under 5 years old in Qibin district from 2000 to 2009* (2000-2009 年淇滨区 5 岁以下儿童死亡结果调查). Chronic Pathematology Journal**	**11,048**

							(慢性病学杂志). 12(10): 1365-1366	
**C70**	**Sun SJ**	**2010**	**Liaoning**	**1 county Jianping**	**Urban and Rural**	**5 years (2005-2009)**	**Su-Juan Sun 孙素娟 (2010). An analysis of death monitoring of children under 5 years old in Jianping county from 2005 to 2009* (2005-2009 年建平县 5 岁以下儿童死亡监测分析). Jiangsu Health Care (江苏卫生保健). 12(3): 39-40**	**24,612**
**C71**	**Tang CF**	**2010**	**Guangxi**	**1 county Quanzhou**	**Urban and Rural**	**8 years (2001-2008)**	**Chen-Fen Tang 唐晨芬 (2010). An analysis of the death monitoring results of children under 5 years old in Quanzhou county, China from 2001 to 2008* (全州县 2001～2008 年 5 岁以下儿童死亡监测结果分析). Medical Innovation of China (中国医学创新). 7(8): 49-50**	**65,918**
**C72**	**Wang MZ**	**2010**	**Zhejiang**	**1 city Cixi**	**Urban and Rural**	**18 years (1991-2008)**	**Mi-Zhen Wang 汪咪珍 (2010). An analysis of death causes of children under 5 years old in Cixi city, China from 1991 to 2008* (慈溪市 1991-2008 年 5 岁以下儿童死因分**	**160,625**

							析). Zhejiang Prev Med (浙江预防医学). 122(2): 66-67	
C73	Wang EX et al.	2010	Shandong	1 city Zhangqiu	Urban and Rural	10 years (2000-2009)	En-Xia Wang, Xia Dong, et al. 王恩霞,董霞,等 (2010). An analysis of death causes of children under 5 years old in Zhangqiu city, China from 2000 to 2009* (章丘市 2000～2009 年 5 岁以下儿童死因分析). Chin J Mod Drug Appl (中国现代药物应用). 4(20): 260	96,236
C74	Wang L	2010	Henan	1 city Jiyuan	Urban and Rural	4 years (2005-2008)	Li Wang 王莉 (2010). An analysis of deaths of children under 5 years old in Jiyuan city* (济源市 5 岁以下儿童死亡分析). J Med Theor & Prac (医学理论与实践). 23(3): 372	24,950
C75	Wang W et al.	2010	Jilin	1 province Jilin	Urban and Rural	10 years (1999-2008)	Wei Wang, Xi-Lian Ni, et al. 王巍,倪锡莲,等 (2010). An analysis of death monitoring result of children under 5 years old in Jilin province, China from 1999 to 2008*	361,168

							(1999—2008 年吉林省 5 岁以下儿童死亡监测结果分析). CJCHC (中国儿童保健杂志). 18(3): 250-252	
C76	Wang YH	2010	Jilin	1 district Chuanying in Jilin city	Urban and Rural	10 years (1999-2008)	Yi-Han Wang 王乙涵 (2010). An analysis of deaths of children under 5 years old in Chuanying district, Jilin city, China from 1999 to 2008* (吉林市船营区 1999 年至 2008 年 5 岁以下儿童死亡分析). China Foreign Medical Treatment (中外医疗). (28): 48-49	24,737
C77	Xu H	2010	Jilin	10 counties in Jilin city	Urban and Rural	9 years (01/2001-12/2009)	Hong Xu 徐红 (2010). An analysis of deaths of children under 5 years old in Jilin city, China from 2001 to 2009* (2001～2009 年吉林市 5 岁以下儿童死亡分析). Chin J Mod Drug Appl (中国现代药物应用). 4(21): 245-246	240,548
C78	Ye MF	2010	Zhejiang	3 streets and 16 towns	Urban and Rural	5 years (2004-2008)	Mei-Fen Ye 叶美芬 (2010). An analysis of death causes of children under 5 years old in Longquan city from 2004 to 2008* (龙泉市 2004—	13,516

							2008 年 5 岁以下儿童死因分析). Shanghai Journal of Preventive Medicine (上海预防医学). 22(2): 117-118	
C79	Zhang JL et al.	2010	Hebei	8 towns in Qianxi county	Rural	7 years (2001-2007)	Jun-Lan Zhang, Kui-Shan Ma 张俊兰,马奎山 (2010). Analysis and countermeasures of death causes of children under 5 years old* (5 岁以下儿童死因分析及对策). Maternal & Child Health Care of China (中国妇幼保健). 25(28): 4064-4065	18,927
C80	Zhang LL	2010	Hunan	31 towns in Hunan province	Urban and Rural	6 years (2004-2009)	Ling-Li Zhang 张伶俐 (2010). Analysis on Death Surveillanee for Children Under 5 Yeasr Old from 2004-2009 in Hunan province (湖南省 2004～2009 年 5 岁以下儿童死亡监测分析). Central South University (中南大学). ():	210,265
C81	Zhang YC	2010	Sichuan	1 county Jintang	Urban and Rural	4 years (01/2006-12/2009)	Yu-Chi Zhang 张宇驰 (2010). Analysis of mortality trends of children under age of five in some country from 2006 to 2009 (某县 2006～	24,919

							2009 年 5 岁以下儿童死亡变化趋势分析). China Prac Med (中国实用医药). 5(34): 237-238	
C82	Zhao YP et al.	2010	Ningxia	1 district Xingqing in Yinchuan city	Urban	10 years (01/01/1998-31/12/2007)	Ya-Ping Zhao, Qin Li, et al. 赵亚萍,李勤,等 (2010). An analysis of deaths of children under 5 years old in Xingqing district, Yinchuan city from 1998 to 2007* (银川市兴庆区 1998～2007 年 5 岁以下儿童死亡分析). Maternal & Child Health Care of China (中国妇幼保健). 25(12): 1669-1670	5,064
C83	An XY et al.	2011	Beijing	1 district Xuanwu in Beijing city	Urban	8 years (01/2003-12/2010)	Xiao-Yun An, Hong Cui, et al. 安晓云,崔红,等 (2011). An analysis of death reviewing of children under 5 years old in Xuanwu district, Beijing city* (北京市宣武区 5 岁以下儿童死亡评审分析). Maternal & Child Health Care of China (中国妇幼保健). 26(32): 4972-4974	18,598
C84	Cao WX et al.	2011	Gansu	2 districts and 3 counties in Baiyin city	Urban and Rural	6 years (2003-2008)	Wen-Xia Cao, Xiao-Xia Zhao 曹文霞,赵晓霞	92,774

							(2011). An analysis of monitoring deaths of children under 5 years old in Baiyin city,China from 2003 to 2008* (2003～2008 年白银市 5 岁以下儿童死亡监测情况). Maternal & Child Health Care of China (中国妇幼保健). 26(16): 2468-2469	
C85	Zha DY et al.	2011	Guangdong	1 district Baiyun in Guangzhou city	Urban	5 years (01/10/2006-30/09/2010)	Da-Yong Zha, Dong-Ling Gu, et al. 查达永,古冬玲,等 (2011). The death cause analysis for children under 5 years old of Baiyun District in Guangzhou City (广州市白云区 2006～2010 年 5 岁以下儿童死因分析). China Modern Medicine (中国当代医药). 18(22): 154-155	35,192
C86	Chen LN	2011	Guangdong	1 district Fanyu in Guangzhou city	Urban	4 years (2007-2010)	Lun-Neng Chen 陈伦能 (2011). Analysis and preventive measures for the death of children under 5 years old in panyu district (番禺区 5 岁以下儿童死亡情况分析与预防措施). CJCHC (中	105,695

							国儿童保健杂志). 19(6): 578-580	
**C87**	**Dong SY**	**2011**	**Jiangsu**	**1 district Quanshan in Xuzhou city**	**Urban**	**4 years (2007-2010)**	**Shu-Ying Dong 董淑英 (2011). An analysis of death monitoring of children under 5 years old in Quanshan district, Xuzhou from 2007 to 2010* (2007—2010 年徐州市泉山区 5 岁以下儿童死亡监测分析). Chin J School Doctor (中国校医). 25(8): 593-594**	**17,427**
**C88**	**Dong YL et al.**	**2011**	**Xinjiang**	**1 city Kelamayi**	**Urban and Rural**	**6 years (2003-2008)**	**Yue-Lian Dong, Li Zhou 董月莲,周丽 (2011). An analysis of deaths of children under 5 years old in Kelamayi city, China from 2003 to 2008* (克拉玛依市 2003～2008 年 5 岁以下儿童死亡分析). Maternal & Child Health Care of China (中国妇幼保健). 26(28): 4371-4372**	**14,868**
**C89**	**Duan CW**	**2011**	**Henan**	**1 city Pingdingshan**	**Urban and Rural**	**5 years (2004-2008)**	**Chuan-Wei Duan 段传伟 (2011). An analysis of deaths of children under 5 years old in Pingdingshan city, China* (平顶山市 5 岁以下儿童死亡情况分**	**209,254**

							析). Maternal & Child Health Care of China (中国妇幼保健). 26(28): 4358-4359	
C90	Fu XY	2011	Jiangxi	rural areas in Yushui district	Rural	4 years (2005-2008)	Xiao-Yi Fu 傅小艺 (2011). Analysis of monitoring result on the death of children under five during the years from 2005 to 2008 in rural area of Yushui district of Xinyu city (新余市渝水区农村 2005～2008 年 5 岁以下儿童死亡监测结果). Maternal & Child Health Care of China (中国妇幼保健). 26(19): 2941-2943	31,137
C91	Gong ZK et al.	2011	Guangxi	1 city Nanning	Urban and Rural	6 years (2004-2009)	Zu-Kang Gong, Wu Jiang, et al. 龚祖康,蒋武,等 (2011). Death surveillance among children aged <5 years in Nanning, 2004-2009 (2004-2009 年南宁市 5 岁以下儿童死亡监测分析). J Prev Med Inf (预防医学情报杂志). 27(4): 290-293	525,805
C92	Guo F et al.	2011	Anhui	4 districts and 3 counties in Hefei city	Urban and Rural	6 years (2003-2008)	Feng Guo, Su-Lin Fu, et al. 郭锋,傅苏林,等 (2011). Mortality of children	130,960

							under 5 years old in Hefei city from 2003 to 2008 (合肥市 2003-2008 年<5 岁儿童死亡监测分析). Chin J Public Health (中国公共卫生). 27(6): 706-707	
C93	Huang XY et al.	2011	Yunnan	1 city Qujing	Urban and Rural	5 years (2002-2006)	Xiao-Yan Huang, Yu-E Lu 黄晓燕,吕玉娥 (2011). An analysis of monitoring deaths of children under 5 years old in Qujing city, China* (曲靖市 5 岁以下儿童死亡监测结果分析). Maternal & Child Health Care of China (中国妇幼保健). 26(22): 3430-3431	345,782
C94	Li DS et al.	2011	Liaoning	1 district Dadong in Shenyang city	Urban	5 years (2005-2009)	Dong-Song Li, Zhu-Shan Qian 李冬松, 钱铸山 (2011). 2005-2009 below the big east area five years old the child died analysis (2005 年-2009 年大东区五岁以下儿童死亡分析). Chinese Manipopulation & Rehabilitation Medicine (按摩与康复医学（下旬刊）). 2(1): 238-239	19,176
C95	Li JC	2011	Qinghai	1 county Huzhu	Urban and Rural	10 years (2001-2010)	Ji-Cun Li 李积存 (2011). An analysis of death	45,420

							causes of children under five years old from 2001 to 2005* (2001-2005 年 5 岁以下儿童死亡原因分析). Chinese Journal of Rural Medicine (中国农村医学杂志). 9(1): 78-80	
C96	Liao J et al.	2011	Guangdong	32 districts and towns in Dongguan city	Urban and Rural	5 years (2004-2008)	Jie Liao, Jian-Xin Liu, et al. 廖捷,刘建新,等 (2011). An analysis of death causes of children under 5 years old in Dongguan xity, Guangdong province from 2004 to 2008* (广东省东莞市 2004～2008 年 5 岁以下儿童死亡原因分析). Maternal & Child Health Care of China (中国妇幼保健). 26(4): 572-573	85,619
C97	Liu D et al.	2011	Zhejiang	2 districts and 1 county in Shaoxing city	Urban and Rural	3 years (2006-2008)	Dan Liu, Hong Yu 刘丹,余红 (2011). Analysis on monitoring result of death of children under 5 years in Shaoxing city from 2006 to 2008 (绍兴市 2006～2008 年 5 岁以下儿童死亡监测结果分析). Maternal & Child Health Care of China (中国妇幼	33,556

							保健). 26(35): 5533-5534	
**C98**	**Liu GQ**	**2011**	**Anhui**	**1 county Feidong**	**Urban and Rural**	**4 years (01/10/2006-30/09/2010)**	**Guo-Qin Liu 刘国琴 (2011). An analysis of deaths of children under 5 years old in one county, China from 2006 to 2010* (某县 2006 年至 2010 年 5 岁以下儿童死亡分析). Guide of China Medicine (中国医药指南). 9(13): 288-289**	**55,992**
**C99**	**Liu J**	**2011**	**Shandong**	**2 streets and 9 towns in Linyi county**	**Urban and Rural**	**10 years (01/06/1999-31/05/2009)**	**Jing Liu 刘静 (2011). An analysis of monitoring deaths of children under 5 years old in Linyi county, China from 1999 to 2009* (1999～2009 年临邑县 5 岁以下儿童死亡监测分析). Maternal & Child Health Care of China (中国妇幼保健). 26(28): 4366-4367**	**59,804**
**C100**	**Liu J et al.**	**2011**	**Liaoning**	**1 city Fuxin**	**Urban and Rural**	**3 years (2007-2009)**	**Jing Liu, Shu-Ping Li, et al. 刘静,李淑萍,等 (2011). An analysis of deaths of 305 neonates* (305 例新生儿死亡情况分析). Contemporary Medicine (当代医学). 17(19): 92-93**	**36,861**
**C101**	**Liu Y**	**2011**	**Shandong**	**8 districts and**	**Urban and**	**8 years (2001-2008)**	**Yan Liu 刘燕 (2011). An**	**442,602**

				**counties in Liaocheng city**	**Rural**		**analysis of monitoring deaths of children under 5 years old in Liaocheng city, China from 2001 to 2008* (聊城市 2001～2008 年 5 岁以下儿童死亡监测分析). Maternal & Child Health Care of China (中国妇幼保健). 26(36): 5778-5780**	
**C102**	**Lu YF et al.**	**2011**	**Jiangsu**	**1 city Yangzhong**	**Urban and Rural**	**10 years (2001-2010)**	**Yue-Fen Lu, Hong-Lian Zhu 陆月芬,朱红莲 (2011). Monitoring analysis of death of children under 5 in Yangzhong (扬中市 5 岁以下儿童死亡监测分析). CJCHC (中国儿童保健杂志). 19(9): 858-860**	**19,178**
**C103**	**Ma JF et al.**	**2011**	**Ningxia**	**6 counties in Yinchuan city**	**Urban and Rural**	**1 year (2010)**	**Jin-Feng Ma, Li-Jun Ha, et al. 马金凤,哈丽君,等 (2011). An analysis of death factors of children under 5 years old in Yinchuan city in 2010* (银川市 2010 年 5 岁以下儿童死亡因素分析). Ningxia Med J (宁夏医学杂志). 33(12): 1250-1251**	**14,230**
**C104**	**Ma XZ**	**2011**	**Gansu**	**1 county Kangle**	**Urban and**	**6 years (2005-2010)**	**Xiu-Zhen Ma 马秀珍**	**18,504**

					Rural		(2011). Analysis and corresponding suggestions of death causes of children under 5 years old in Kangle county, China from 2005 to 2010* (康乐县 2005～2010 年 5 岁以下儿童死亡原因分析与建议). Chin J Mod Drug Appl (中国现代药物应用). 5(11): 138-139	
C105	Mao XM	2011	Gansu	1 district Xifeng in Qingyang city	Urban	10 years (2000-2009)	Xiang-Ming Mao 毛向明 (2011). An analysis of death causes of children under 5 years old in Xifeng district, China from 2000 to 2009* (西峰区 2000～2009 年度 5 岁以下儿童死亡原因分析). Chinese Community Doctors (中国社区医师). 13(26): 307-309	31,923
C106	Mo ZL	2011	Guangxi	1 county Xincheng	Urban and Rural	6 years (1998-2000,2007-2009)	Zeng-Luan Mo 莫增銮 (2011). An retrospective analysis of deaths of children under 5 years old in Xincheng city, China* (忻城县 5 岁以下儿童死亡回顾性分析). Maternal & Child Health Care of	25,019

							China (中国妇幼保健). 26(32): 5013-5015	
C107	Qian Y et al.	2011	Jiangsu	urban zones in Xuzhou city	Urban	5 years (2005-2009)	Ying Qian, Pei-An Lou, et al. 钱颖,娄培安,等 (2011). An analysis of death causes of children under 5 years old in Xuzhou city, China from 2005 to 2009* (2005—2009 年徐州市 5 岁以下儿童死因分析). Chin J School Doctor (中国校医). 25(8): 592-594	78,743
C108	Rong H et al.	2011	Sichuan	1 city Zigong	Urban and Rural	4 years (2006-2009)	Hua Rong, Lei Zhu, et al. 荣华,朱蕾,等 (2011). Analysis of death causes of children under 5 years old from 2006 to 2009 in Zigong (2006-2009 年自贡市 5 岁以下儿童死亡变化趋势与分析). Journal of Luzhou Medical College (泸州医学院学报). 34(2): 181-183	96,237
C109	Su GY	2011	Sichuan	1 county Butuo	Urban and Rural	5 years (2004-2008)	Guang-Yu Su 苏光玉 (2011). Investigation analysis of death cases of children under 5 years old in Butuo county from 2005 to 2008* (2005-2008 年布拖县 5 岁以下儿童死	14,942

							**亡病例调查分析). Chin J of Clinical Rational Drug Use (临床合理用药杂志). 4(9A): 125-126**	
**C110**	**Sun FL et al.**	**2011**	**Hebei**	**17 towns in She county**	**Rural**	**9 years (2001-2009)**	**Feng-Lan Sun, Yun-Ping Li, et al. 孙凤兰,李云平,等 (2011). An analysis of deaths of children under 5 years old in She county, China from 2001 to 2009* (涉县 2001～2009 年 5 岁以下儿童死亡分析). Maternal & Child Health Care of China (中国妇幼保健). 26(10): 1483-1485**	**38,888**
**C111**	**Tan ZL**	**2011**	**Liaoning**	**1 city Yingkou**	**Urban and Rural**	**2 years (2001 and 2010)**	**Zhen-Lai Tan 谭振来 (2011). An analysis of the survival and health conditions of children under 5 years old in Yingkou city, China in 2010s* (21 世纪 10 年代营口市 5 岁以下儿童生存和健康状况分析). Chin Pediatr Integr Tradit West Med (中国中西医结合儿科学). 3(2): 187-188**	**33,018**
**C112**	**Wang B**	**2011**	**Henan**	**1 city Jiaozuo**	**Urban and Rural**	**5 years (10/2004-09/2009)**	**Bing Wang 王冰 (2011). An analysis of death causes of children under 5**	**178,484**

							**years old in Jiaozuo city, China from 2005 to 2009* (焦作市 2005-2009 年 5 岁以下儿童死亡原因分析). Chinese Journal of Coal Industry Medicine (中国煤炭工业医学杂志). 17(7): 1029-1030**	
**C113**	**Wang X et al.**	**2011**	**Jilin**	**10 districts and counties in Jilin city**	**Urban and Rural**	**9 years (01/10/2001-30/09/2010)**	**Xin Wang, Yuan-Ping Ding 王新,丁元萍 (2011). Analysis on result of under 5 mortality surveillance of Jilin city 2002-2010 (2002-2010 年吉林市 5 岁以下儿童死亡监测结果分析). Chin J Women Child Health (中国妇幼卫生杂志). 2(2): 77-79**	**253,977**
**C114**	**Wang XR**	**2011**	**Neimenggu**	**1 city Xinganmeng**	**Urban and Rural**	**5 years (2005-2009)**	**Xiu-Rong Wang 王秀荣 (2011). 2005 to 2009, deaths of children under age 5 Xinganmeng Analysis (2005-2009 年兴安盟 5 岁以下儿童死亡分析). National Medical Frontiers of China (中国医疗前沿). 6(18): 91-92**	**72,191**
**C115**	**Wei BH et al.**	**2011**	**Xinjiang**	**11 streets and 2 towns in Xinshi district**	**Urban and Rural**	**5 years (2005-2009)**	**Bao-Hua Wei, Zhi-Ru Xu, et al. 韦宝华,徐志茹,等**	**28,615**

							**(2011). An analysis of deaths of children under 5 years old in Xinshi district, China from 2005 to 2009* (新市区 2005～2009 年 5 岁以下儿童死亡结果分析). Chinese Journal of Reproductive Health (中国生育健康杂志). 22(4): 220, 222**	
**C116**	**Wu MH et al.**	**2011**	**Shanxi**	**1 county Taigu**	**Urban and Rural**	**20 years (1990-2009)**	**Mei-Hua Wu, Jun-Li Du, et al. 武梅花,杜俊丽,等 (2011). Analysis on death causes of children under age 5 in Taigu county from 1990 to 2009 (1990 年至 2009 年太谷县 5 岁以下儿童死亡原因分析). Journal of Shanxi Medical College for Continuing Education (山西职工医学院学报). 21(1): 59-61**	**64,140**
**C117**	**Xiang XS**	**2011**	**Shaanxi**	**1 city Xian**	**Urban and Rural**	**10 years (2000-2009)**	**Xiao-Mei Xiang 相晓妹 (2011). An analysis of death tendency of children under 5 years old in Xi'an city, China from 2000 to 2009* (西安市 2000～2009 年 5 岁以下儿童死亡趋势分析).**	**399,068**

							**Maternal & Child Health Care of China (中国妇幼保健). 26(7): 965-967**	
**C118**	**Xiang ZY**	**2011**	**Hunan**	**1 county Cili**	**Urban and Rural**	**5 years (2005-2009)**	**Zuo-Ying Xiang 向左英 (2011). Observation and analysis of mortality status of children under 5 years in Cili county from 2005 to 2009 (慈利县 2005—2009 年 5 岁以下儿童死亡监测分析). Chinese Primary Health Care (中国初级卫生保健). 25(2): 42-44**	**30,202**
**C119**	**Xiao FX**	**2011**	**Jiangxi**	**10 districts and counties in Nanchang city**	**Urban and Rural**	**4 years (2000,2006-2008)**	**Feng-Xian Xiao 肖凤仙 (2011). An analysis of monitoring deaths of children under 5 years old in Nanchang, China from 2000 to 2008* (2000～2008 年南昌市 5 岁以下儿童死亡监测资料分析). Maternal & Child Health Care of China (中国妇幼保健). 26(21): 3269-3270**	**43,551**
**C120**	**Xu L**	**2011**	**Qinghai**	**1 district Chengdong in Xining city**	**Urban**	**5 years (2006-2010)**	**Ling Xu 徐灵 (2011). Analysis of death cause of children under the age of five in a district from 2006 to 2010 (城东区 2006 年-**	**6,731**

							2010 年 5 岁以下儿童死因分析). Chinese Medical Record (中国病案). 12(11): 45-46	
C121	Yang XF et al.	2011	Gansu	22 towns in Ganzhou district	Rural	5 years (2005-2009)	Xiao-Fang Yang, Xiao-Ying Zhang, et al. 杨晓芳, 张晓英,等 (2011). An analysis of deaths of children under 5 years old in Ganzhou district, Zhangye city from 2005 to 2009* (张掖市甘州区 2005～2009 年 5 岁以下儿童死亡分析). Chinese Community Doctors (中国社区医师). 13(8): 234-235	23,575
C122	Zhang D et al.	2011	Hubei	1 city Wuhan	Urban and Rural	9 years (2001-2009)	Dan Zhang, Shao-Ping Yang 张丹,杨少萍 (2011). An analysis of deaths of children under 5 years old in Wuhan, China from 2001 to 2009* (武汉市 2001～2009 年 5 岁以下儿童死亡情况分析). Maternal & Child Health Care of China (中国妇幼保健). 26(8): 1132-1134	331,684
C123	Zhang XR	2011	Shandong	1 city Zaozhuang	Urban and Rural	8 years (2002-2009)	Xiao-Ru Zhang 张晓茹 (2011). Analysis of death causes and study on the	307,238

							**intervention measures in children under 5 years old (3677 例 5 岁以下儿童死亡原因分析). China Medicine (中国医药). 6(5): 610-612**	
**C124**	**Zhang XL et al.**	**2011**	**Shanxi**	**13 counties in Changzhi city**	**Urban and Rural**	**9 years (2000-2008)**	**Xin-Lan Zhang, Jin-Yan Li, et al. 张新兰,李晋艳,等 (2011). An analysis of monitoring deaths of children under 5 years old in Changzhi city, China from 2000 to 2008* (2000～2008 年长治市 5 岁以下儿童死亡监测结果分析). Maternal & Child Health Care of China (中国妇幼保健). 26(26): 4041-4043**	**241,208**
**C125**	**Zhang Y et al.**	**2011**	**Zhejiang**	**1 city Wenling**	**Urban and Rural**	**5 years (2006-2010)**	**Yi Zhang, Liao-Liao Wang 张奕,王燎燎 (2011). An analysis of death causes of children under 5 years old in Wenling city from 2006 to 2010* (2006～2010 年温岭市 5 岁以下儿童死因分析). China's health statistics annual conference in 2011 (2011 年中国卫生统计学**	**57,222**

							年会). (3):	
**C126**	**Zhang Y**	**2011**	**Shanxi**	**1 city Yuncheng**	**Urban and Rural**	**1 year (2009)**	**Yu Zhang 张玉 (2011). Report of death monitoring analysis of children under 5 years old in Yuncheng city in 2009* (运城市 2009 年 5 岁以下儿童死亡监测分析报告). The Medical Forum (基层医学论坛). 15(11): 290-292**	**49,456**
**C127**	**Zhao XX et al.**	**2011**	**Gansu**	**16 counties and 72 towns in Gansu province**	**Urban and Rural**	**5 years (2001-2005)**	**Xiao-Xia Zhao, Wen-Xia Cao 赵晓霞,曹文霞 (2011). An analysis of monitoring deaths of children under 5 years old in Gansu province, China from 2001 to 2005* (2001～2005 年甘肃省 5 岁以下儿童死亡监测情况). Maternal & Child Health Care of China (中国妇幼保健). 26(28): 4354-4356**	**74,137**
**C128**	**Zhuang ZZ et al.**	**2011**	**Fujian**	**1 city Quanzhou**	**Urban and Rural**	**4 years (2006-2009)**	**Zhen-Zhang Zhuang, Li-Na Chen, et al. 庄镇漳,陈丽娜,等 (2011). Monitoring analysis of children death under 5 years old from 2006 - 2009**	**78,765**

							**in Quanzhou (2006-2009 年泉州市 5 岁以下儿童死亡监测分析). Henan J Prev Med (河南预防医学杂志). 22(2): 84-85**	
**C129**	**Cai ML et al.**	**2012**	**Guangdong**	**1 district Luohu in Shenzhen city**	**Urban**	**5 years (01/10/2005-30/09/2010)**	**Miao-Ling Cai, Hong-Yan Zeng 蔡妙玲,曾红燕 (2012). Causes of death of children under five years old in Luohu district from 2006 to 2010 and the interventional measures (2006～2010 年罗湖区 5 岁以下儿童死亡原因与干预措施). Maternal & Child Health Care of China (中国妇幼保健). 27(21): 3224-3227**	**58,758**
**C130**	**Cen RZ**	**2012**	**Guangxi**	**11 counties in Hechi city**	**Urban and Rural**	**3 years (2009-2011)**	**Ruo-Zhu Cen 岑若珠 (2012). An analysis of neonatal deaths in Hechi city, China from 2009 to 2011* (河池市 2009～2011 年新生儿死亡情况分析). Maternal & Child Health Care of China (中国妇幼保健). 27(16): 2417-2418**	**165,828**
**C131**	**Chang LZ**	**2012**	**Henan**	**1 city Xuchang**	**Urban and Rural**	**10 years (2001-2010)**	**Lian-Zhi Chang 常连枝 (2012). An analysis of**	**403,985**

							deaths of children under 5 years old in Xuchang city, China for a decade from 2001 to 2010* (许昌市 2001 年至 2010 年 5 岁以下儿童 10 年死亡情况分析). China Foreign Medical Treatment (中外医疗). (8): 126	
C132	Chen FP et al.	2012	Shaanxi	1 county Nanzheng	Urban and Rural	11 years (2000-2010)	Feng-Ping Chen, Xiao-Ju Hu 陈凤萍,胡晓菊 (2012). An analysis of death causes of children under 5 years old in Nanzheng county, China from 2000 to 2010* (南郑县 2000～2010 年 5 岁以下儿童死亡原因分析). Maternal & Child Health Care of China (中国妇幼保健). 27(36): 5950-5952	50,738
C133	Chen Y	2012	Hubei	1 county Zhushan	Urban and Rural	3 years (2009-2011)	Yan Chen 陈艳 (2012). Analysis and interventions of death causes of children under 5 years old in Zhushan county* (竹山县 5 岁以下儿童死亡原因调查分析及干预). World Health Digest (中外健康文摘).	17,370

							9(48): 377-378	
C134	Chen ZP	2012	Guangxi	1 county Binyang	Urban and Rural	7 years (2004-2010)	Zhong-Ping Chen 陈忠平 (2012). An analysis of death causes of children under 5 years old from 2004 to 2010* (2004～2010 年 5 岁以下儿童死因分析). Chinese Community Doctors (中国社区医师). 14(8): 380	82,776
C135	Qiu YL et al.	2012	Jiangsu	1 city Suqian	Urban and Rural	10 years (2001-2010)	Ya-Li Qiu, Xi-Min Bai, et al. 仇亚丽,白希敏,等 (2012). An analysis of death causes of children under 5 years old in one city, China from 2001 to 2010* (2001 至 2010 年某市 5 岁以下儿童死亡原因分析). Guide of China Medicine (中国医药指南). 10(1): 88-90	466,813
C136	Dai WX et al.	2012	Jiangsu	1 county Sihong	Urban and Rural	6 years (2006-2011)	Wen-Xian Dai, Jin-Shuang Liu, et al. 戴文献,刘金双,等 (2012). An analysis of death tendency and causes of children under 5 years old in Sihong county, China from 2006 to 2011* (2006 年至 2011 年泗洪县 5 岁	82,967

							以下儿童死亡趋势与原因分析). Journal of Chinese Physician (中国医师杂志). 14(11): 1515-1517	
C137	Deng H et al.	2012	Jiangxi	1 city Xinyu	Urban and Rural	5 years (2007-2011)	Hong Deng, Xiao-Fang Yuan, et al. 邓红,袁小芳,等 (2012). An analysis of death causes and mortality tendency of children under 5 years old in Xinyu city from 2007 to 2011* (新余市 2007—2011 年 5 岁以下儿童各期死亡率变化趋势及死因分析). JCM (社区医学杂志). 10(17): 51-52	80,000
C138	Deng SH et al.	2012	Guangxi	1 city Baise	Urban and Rural	1 year (2010)	Shi-Hua Deng, Ming-Gang Ban, et al. 邓仕华,班明刚,等 (2012). An analysis of deaths of children under 5 years old in Baise city, Guangxi province in 2010* (广西百色市 2010 年 5 岁以下儿童死亡分析). Maternal & Child Health Care of China (中国妇幼保健). 27(14): 2089-2091	51,548
C139	Fu YQ et al.	2012	Guangdong	1 street Xixiang in Shenzhen city	Urban	5 years (2007-2011)	Yan-Qing Fu, Wei Wang 傅燕青,王维 (2012).	56,851

							**Analysis and interventions of death causes of children under 5 years old in Xixiang street, China from 2007 to 2011* (西乡街道 2007—2011 年 5 岁以下儿童死亡原因分析与干预措施). China Health Industry (中国卫生产业). 9(34): 172-173**	
**C140**	**Gao JX**	**2012**	**Jiangsu**	**26 towns in Pei county**	**Rural**	**10 years (11/2002-9/2011)**	**Jian-Xia Gao 高建霞 (2012). An analysis of deaths of children under 5 years old in Pei county, China from 2002 to 2011* (沛县 2002～2011 年 5 岁以下儿童死亡分析). Chinese Community Doctors (中国社区医师). 14(28): 141**	**135,559**
**C141**	**Ge GL**	**2012**	**Neimenggu**	**1 district Saihan in Huhehaote city**	**Urban**	**5 years (2005-2009)**	**Gai-Ling Ge 葛改玲 (2012). An analysis of death causes of children under five years old* (5 岁以下儿童死因分析). China Health Care & Nutrition (中国保健营养). 22(12): 5351**	**13,167**
**C142**	**Gu YJ**	**2012**	**Jiangsu**	**9**	**Urban and**	**11 years (2001-2011)**	**Yu-Jing Gu 顾宇静**	**487,224**

				cities/districts/counties in Wuxi city	Rural		(2012). An analysis of deaths monitoring and revies of children under 5 years old in Wuxi city, China from 2001 to 2010* (2001～2011 年无锡市 5 岁以下儿童死亡监测和评审结果分析). Maternal & Child Health Care of China (中国妇幼保健). 27(13): 1990-1991	
C143	Gui J	2012	Jiangsu	1 district Yuhuatai in Nanjing city	Urban	7 years (2003-2009)	Jie Gui 桂捷 (2012). An analysis of deaths of children under 5 years old in Yuhuatai district, Nanjing city from 2003 to 2009* (2003-2009 年南京市雨花台区 5 岁以下儿童死亡分析及干预措施). World Health Digest (中外健康文摘). 9(33): 56-58	15,718
C144	He GM	2012	Yunnan	1 county Deqin	Urban and Rural	7 years (2004-2010)	Gui-Mei He 和桂梅 (2012). An retrospective analysis of deaths of children under 5 years old in Deqin county, China from 2004 to 2010* (德钦县 2004 年-2010 年 5 岁以下儿童死亡回顾分析). Health Horizon•Medical	5,876

							Sciences (健康大视野：医学版). (1): 32-33	
C145	He YQ	2012	Yunnan	1 district, 4 counties and 16 towns in Lijiang city	Urban and Rural	5 years (2007-2011)	Yu-Qiong He 和玉琼 (2012). An analysis of mornitoring deaths of children under 5 years old in Lijiang city, China from 2007 to 2011* (2007～2011 年丽江市 5 岁以下儿童死亡监测分析). Chinese Community Doctors (中国社区医师). 14(11): 414-415	16,813
C146	Hou H et al.	2012	Xinjiang	7 districts and 1 county in Wulumuqi city	Urban and Rural	5 years (2006-2010)	Hong Hou, Xin-Hua Liu, et al. 侯红,刘新华,等 (2012). An analysis of monitoring deaths of children under 5 years old in Wulumiqi city from 2006 to 2010* (乌鲁木齐市 2006～2010 年 5 岁以下儿童死亡监测分析). Xinjiang Medical Journal (新疆医学). 42(4): 113-115	155,669
C147	Huang AF	2012	Zhejiang	1 county Yuhuan	Urban and Rural	10 years (2000-2009)	Ai-Fang Huang 黄爱芳 (2012). An analysis of deaths of children under 5 years old in Yuhuan county, China from 20003 to 2009* (2000-2009 年玉	42,539

							环县 5 岁以下儿童死亡状况分析). World Health Digest (中外健康文摘). 9(35): 53-54	
C148	Hui F et al.	2012	Ningxia	1 city Guyuan	Urban and Rural	4 years (2006-2009)	Fang Hui, Xia Hai 惠芳, 海霞 (2012). Analysis on death of children under five years and interventional measures in Guyuan city from 2006 to 2009 (固原市 2006～2009 年度 5 岁以下儿童死亡分析及干预措施). Maternal & Child Health Care of China (中国妇幼保健). 27(3): 331-333	89,899
C149	Jiang XM et al.	2012	Zhejiang	1 county Kaihua	Urban and Rural	5 years (2006-2010)	Xiao-Mei Jiang, Wei-Tao Zhang, et al. 江晓梅,张渭桃,等 (2012). Death monitoring among children under five in Kaihua county from 2006 to 2010 (2006-2010 年开化县 5 岁以下儿童死亡监测分析). Chinese Rural Health Service Administration (中国农村卫生事业管理). 32(7): 748-751	18,093
C150	Li CX	2012	Guangdong	1 district Xinhui in	Urban	10 years (10/2000-	Cai-Xia Li 李彩霞 (2012).	65,876

				**Jiangmen city**		**09/2010)**	**Retrospective analysis of deaths of children under 5 years old in Xinhui district, China from 2001 to 2010* (新会区 2001～2010 年 5 岁以下儿童死亡回顾性分析). Chinese Community Doctors (中国社区医师). 14(23): 319-320**	
**C151**	**Li DM et al.**	**2012**	**Xinjiang**	**1 state Changji**	**Urban and Rural**	**1 year (2011)**	**Dong-Mei Li, Dong-Dong Li 李冬梅,李冬东 (2012). An analysis of monitoring deaths of children under 5 years old in Changji Autonomous Prefecture* (昌吉州 5 岁以下儿童死亡监测分析). World Health Digest (中外健康文摘). 9(35): 56-57**	**11,633**
**C152**	**Li HH et al.**	**2012**	**Guangxi**	**4 districts and 6 counties in Liuzhou city**	**Urban and Rural**	**9 years (01/10/2003-30/09/2011)**	**Hong-Hui Li, Zheng Nong, et al. 李红辉,农铮,等 (2012). Analysis of supervising result of death for the children under the age of 5 in Liuzhou from 2003 to 2011 (柳州市 2003-2011 年 5 岁以下儿童死亡监测结果分析). CJCHC (中国儿童保健杂**	**400,255**

							志). 20(5): 476-477	
**C153**	**Li H et al.**	**2012**	**Shaanxi**	**1 county Chenggu**	**Urban and Rural**	**6 years (2005-2010)**	**Hua Li, Hui Guo 李花,郭辉 (2012). An analysis of death causes of children under 5 years old in Chenggu county from 2005 to 2010* (城固县 2005 年～2010 年 5 岁以下儿童死亡原因分析). Jilin Medical Journal (吉林医学). 33(11): 2332-2333**	**26,830**
**C154**	**Li LL**	**2012**	**Jiangsu**	**14 towns in Guannan county**	**Rural**	**6 years (2006-2011)**	**Li-Li Li 李丽丽 (2012). Death monitoring of children under 5 years old in Guannan county from 2006 to 2011* (2006—2011 年灌南县 5 岁以下儿童死亡监测). Jiangsu Health Care (江苏卫生保健). 14(5): 43-44**	**43,140**
**C155**	**Lin GF et al.**	**2012**	**Guangxi**	**city zones in Beihai city**	**Urban**	**3 years (2009-2011)**	**Gui-Fang Lin, Xuan-Feng Zhong 林桂芳,钟旋风 (2012). Countermeasures of deaths of children under 5 years old* (五岁以下儿童死亡干预措施). Jiankang Bidu (健康必读（下旬刊）). (6): 424**	**77,276**
**C156**	**Liu AY et**	**2012**	**Sichuan**	**1 city Emeishan**	**Urban and**	**11 years (2001-2011)**	**Ai-Yu Liu, Qi Li, et al. 刘**	**28,800**

	**al.**				**Rural**		**爱宇,李琦,等 (2012). Epidemiological analysis on the death of children under 5 in Emeishan city, 2001-2011 (2001-2011 年峨眉山市 5 岁以下儿童死亡分析). Journal of Occupational Health and Damage (职业卫生与病伤). 27(3): 163-165**	
**C157**	**Liu LY**	**2012**	**Neimenggu**	**1 city Wulanhaote**	**Urban and Rural**	**8 years (2003-2010)**	**Li-Yuan Liu 刘丽媛 (2012). Death analysis of 0~5 years old children from 2003 to 2010 in Ulanhot city (乌兰浩特市 2003～2010 年 0～5 岁儿童死亡情况分析). Journal of Inner Mongolia University for Nationalities (内蒙古民族大学学报). 27(4): 483-486**	**17,749**
**C158**	**Liu Y et al.**	**2012**	**Henan**	**1 county Zhongmou**	**Urban and Rural**	**5 years (2006-2010)**	**Yin Liu, Chun-Xia Cang 刘银,仓春霞 (2012). Analysis and preventions of deaths of children under 5 years old in Zhongmou county, China* (中牟县 5 岁以下儿童死亡情况分析与预防措施). Chin J Mod Drug**	**78,013**

							**Appl (中国现代药物应用). 6(18): 135-136**	
**C159**	**Mao XM et al.**	**2012**	**Ningxia**	**13 counties in Ningxia province**	**Urban and Rural**	**10 years (2001-2010)**	**Xin-Mei Mao, Gang Li, et al. 毛新梅,李刚,等 (2012). An analysis of death tendency of children under 5 years old in Ningxia province from 2001 to 2010* (宁夏 2001-2010 年 5 岁以下儿童死亡趋势分析). Journal of Ningxia Medical University (宁夏医科大学学报). 34(12): 1286-1289**	**142,815**
**C160**	**Qin JX**	**2012**	**Guangxi**	**1 city Guilin**	**Urban and Rural**	**1 year (01/10/2010-30/09/2011)**	**Ji-Xiu Qin 秦吉秀 (2012). An analysis of death causes of children under 5 years old in Guilin city, China in 2011* (2011 年桂林市 5 岁以下儿童死亡原因分析). World Health Digest (中外健康文摘). 9(51): 417-418**	**64,009**
**C161**	**Shen WX**	**2012**	**Liaoning**	**1 district Tiexi in Shenyang city**	**Urban**	**5 years (01/10/2006-30/09/2011)**	**Wei-Xing Shen 沈卫星 (2012). An investigation on deaths of children under 5 years old in Tiexi district, Shenyang city from 2007 to 2011* (2007/2011 沈阳市铁西区**	**30,815**

							5 岁以下儿童死亡调查分析). Chin Pediatr Integr Tradit West Med (中国中西医结合儿科学). 4(5): 467-469	
C162	Song FL et al.	2012	Shandong	1 city Jinan	Urban and Rural	7 years (2004-2010)	Feng-Ling Song, Liang-Zheng Yang, et al. 宋风玲, 杨良政,等 (2012). Dynamic analysis and preventive measures of deaths of children under 5 years old in Jinan city, China* (济南市 5 岁以下儿童死亡动态分析与干预措施研究). CJCHC (中国儿童保健杂志). 20(8): 758-760	412,212
C163	Tan M	2012	Yunnan	1 district and 7 counties in Lincang city	Urban and Rural	5 years (2006-2010)	Min Tan 覃敏 (2012). An analysis of death tendency of children under 5 years old in Lincang city from 2006 to 2010* (临沧市 2006～2010 年 5 岁以下儿童死亡趋势分析). Journal of Military Surgeon in Southwest China (西南军医). 14(2): 247-248	119,078
C164	Wang L	2012	Zhejiang	1 city Shangyu	Urban and Rural	10 years (1999-2008)	Li Wang 王丽 (2012). An analysis of death tendency of children under 5 years	59,007

							**old in Shangyu city, China from 1999 to 2008* (1999～2008 年上虞市 5 岁以下儿童死亡变化趋势分析). Maternal & Child Health Care of China (中国妇幼保健). 27(4): 542-543**	
**C165**	**Wang PY**	**2012**	**Zhejiang**	**24 towns in Jingning county**	**Rural**	**7 years (01/10/2004-30/09/2011)**	**Pei-Ying Wang 王佩英 (2012). An analysis of death causes of children under 5 years old in Jingning county, China* (景宁县 5 岁以下儿童死亡原因分析). Zhejiang Prev Med (浙江预防医学). 24(7): 70-72**	**10,812**
**C166**	**Wang Q**	**2012**	**Zhejiang**	**1 city Yiwu**	**Urban and Rural**	**6 years (2006-2011)**	**Qin Wang 王钦 (2012). An analysis of the results of monitoring deaths of children under 5 years old in Yiwu city from 2006 to 2011* (对 2006 年～2011 年义乌市 5 岁以下儿童死亡监测结果的分析). Seek Medical and Ask the Medicine (求医问药(下半月)). 10(11): 1029-1030**	**43,049**
**C167**	**Wei HG et al.**	**2012**	**Gansu**	**1 district Suzhou in Jiuquan city**	**Urban and Rural**	**10 years (2001-2010)**	**Hong-Guang Wei, Bian-Fang Chen, et al. 魏红光,**	**34,828**

							陈边防,等 (2012). An analysis of death causes of children under 5 years old in Suzhou district, Jiuquan city from 2001 to 2010* (酒泉市肃州区 2001—2010 年 5 岁以下儿童死亡原因分析). Health Vocational Education (卫生职业教育). 30(16): 103-104	
C168	Wulayin S et al.	2012	Xinjiang	1 county Shanshan	Urban and Rural	5 years (2004-2008)	Sha-Dai-Ti-Han Wuyinla, Gui-Lan Yang 吾拉音•沙代提汗, 扬挂兰 (2012). An analysis of mortalities of children under 5 years old from 2004 to 2008* (2004-2008 年 5 岁以下儿童死亡率分析). Jiankang Bidu (健康必读（下旬刊）). (5): 455	15,277
C169	Wu Q et al.	2012	Guangdong	1 district Haizhu in Guangzhou city	Urban	10 years (01/10/2000-30/09/2010)	Ji Wu, Xue-Zhen Lu, et al. 吴奇,卢雪珍,等 (2012). An analysis of death infulencing factors of children under 5 years old in Haizhu district, China from 2001 to 2010* (海珠区 2001～2010 年 5 岁以下儿童死亡相关因素分	40,924

							析). Maternal & Child Health Care of China (中国妇幼保健). 27(25): 3935-3937	
C170	Xia BJ et al.	2012	Hebei	1 district Fengrun in Tangshan city	Urban	3 years (10/2008-09/2011)	Bing-Jie Xia, Chun-Ru Xia, et al. 夏冰杰,夏春茹,等 (2012). An analysis of deaths of children under 5 years old in Fengrun district, China from 2008 to 2011* (丰润区 2008～2011 年 5 岁以下儿童死亡情况分析). Chinese Journal of Reproductive Health (中国生育健康杂志). 23(6): 438-440	30,709
C171	Xie Q et al.	2012	Chongqing	1 district Yubei in Chongqing city	Urban	5 years (2007-2011)	Qin Xie, Ling Wei 谢琴,魏玲 (2012). An analysis of death causes of children under 5 years old in Yubei district, Chongqing city from 2007 to 2011* (2007—2011 年重庆市渝北区 5 岁以下儿童死亡原因分析). J Mod Med Health (现代医药卫生). 28(16): 2553-2554	43,150
C172	Xu XH et al.	2012	Jiangsu	1 county Haian	Urban and Rural	5 years (2007-2011)	Xiao-Hong Xu, Jin Lu 徐晓红,卢进 (2012). An analysis of monitoring	25,957

							deaths of children under 5 years old in Haian city, China from 2007 to 2011* (海安县 2007～2011 年 5 岁以下儿童死亡监测分析). Chinese Community Doctors (中国社区医师). 14(31): 363	
C173	Yang KL	2012	Yunnan	1 county Yulong	Urban and Rural	9 years (2003-2011)	Kun-Li Yang 杨昆丽 (2012). An analysis of deaths and related influencing factors of child under 5 years old in Yulong county, Yunnan province* (云南省玉龙县 5 岁以下儿童死亡及相关影响因素分析). Chin J Women Child Health (中国妇幼卫生杂志). 3(6): 356-359	15,527
C174	Yang Y et al.	2012	Ningxia	1 city Lingwu	Urban and Rural	10 years (2000-2009)	Yan Yang, Hai-Ying Liu 杨彦,刘海英 (2012). An analysis of deaths of children under 5 years old in Lingwu city, China* (灵武市 5 岁以下儿童死亡分析). Maternal & Child Health Care of China (中国妇幼保健). 27(1): 73-74	28,138

**C175**	**Yao SJ et al.**	**2012**	**Hubei**	**1 county Hongan**	**Urban and Rural**	**4 years (2008-2011)**	**Shao-Jun Yao, Yi-Hong Wu, et al. 姚少军,吴艺红,等 (2012). An analysis of death investigation results of children under 5 years old in Hongan county from 2008 to 2011* (2008-2011 年红安县 5 岁以下儿童死亡调查结果分析). Yiayao Qianyan (医药前沿). 2(17): 144-145**	**22,931**
**C176**	**Yu H et al.**	**2012**	**Zhejiang**	**1 city Shaoxing**	**Urban and Rural**	**1 year (2011)**	**Hong Yu, Dan Liu 余红,刘丹 (2012). Death surveillance in migrant children aged <5 years in Shaoxing, 2011 (2011 年浙江省绍兴市 5 岁以下流动儿童死亡监测结果分析). Disease Surveillance (疾病监测). 27(11): 903-905**	**43,562**
**C177**	**Yu FY et al.**	**2012**	**Zhejiang**	**1 district Xiaoshan in Hangzhou city**	**Urban**	**5 years (01/10/2005-30/09/2010)**	**Fei-Yan Yu, Dao-Liang Wang 俞飞燕,王道良 (2012). Longitudinal analysis and countermeasures of deaths of children under 5 years old in Xiaoshan district, China* (萧山区 5 岁以下儿童死亡纵向分析及对策). Chinese Rural**	**44,632**

							**Health Service Administration (中国农村卫生事业管理). 32(7): 746-747**	
**C178**	**Zhang HL et al.**	**2012**	**Shaanxi**	**1 county Xixiang**	**Urban and Rural**	**6 years (01/2005-01/2010)**	**Hui-Ling Zhang, Li-Jun Xue 张惠玲, 薛丽君 (2012). Death analysis of children under 5 years of age in Xixiang county from 2005 to 2010 and intervention (西乡县 2005 至 2010 年 5 岁以下儿童死亡分析及干预). Chinese Journal of Women and Child Health Research (中国妇幼健康研究). 23(5): 655-657**	**18,642**
**C179**	**Zhang SQ et al.**	**2012**	**Guangdong**	**1 district Longgang in Shenzhen city**	**Urban**	**6 years (2004-2009)**	**Shao-Qiang Zhang, Ling Zhang, et al. 张绍强, 张玲,等 (2012). Analysis of death of children under age 5 in Longgang district of Shenzhen (深圳市龙岗区 5 岁以下儿童死亡情况分析). Chinese Journal of Women and Child Health Research (中国妇幼健康研究). 23(2): 145-147**	**176,633**
**C180**	**Zhang SX**	**2012**	**Hubei**	**1 city Anlu**	**Urban and Rural**	**5 years (2006-2010)**	**Shu-Xian Zhang 张书先 (2012). An analysis of**	**25,230**

							**deaths of children under 5 years old in Anlu city, China from 2006 to 2010* (安陆市 2006-2010 年五岁以下儿童死亡分析). China Hwalth Care & nutrition (中国保健营养（中旬刊）). (8): 322**	
**C181**	**Zhang Y et al.**	**2012**	**Tianjin**	**1 district Tianqiao in Tianjin city**	**Urban and Rural**	**4 years (2007-2010)**	**Ya Zhang, Mei-Rong Wu 张娅,吴美荣 (2012). An analysis of death records of children under 5 years old in Tianqiao district, Jinan city from 2007 to 2010* (2007～2010 年济南市天桥区 5 岁以下儿童死亡资料分析). Prev Med Trib (预防医学论坛). 18(1): 62-63**	**21,579**
**C182**	**Zhao LL**	**2012**	**Zhejiang**	**1 city Dongyang**	**Urban and Rural**	**3 years (2009-2011)**	**Ling-Ling Zhao 赵玲玲 (2012). An analysis of monitoring death causes of children under 5 years old in Dongyang city from 2009 to 2011* (2009 年～2011 年东阳市 5 岁以下儿童死亡原因监测分析). Seek Medical and Ask the Medicine (求医问药(下半月)). 10(11): 645**	**30,369**

**C183**	**Zhao WX**	**2012**	**Liaoning**	**4 districts and 3 counties in Dandong city**	**Urban and Rural**	**7 years (2005-2011)**	**Wen-Xiu Zhao 赵文秀 (2012). Investigation of children's mortality status under 5 years in Dandong from 2005 to 2011 (丹东市 2005-2011 年 5 岁以下儿童死亡状况调查). Jilin University (吉林大学). ():**	**102,390**
**C184**	**Zhao Y et al.**	**2012**	**Tianjin**	**1 district Hebei in Tianjin city**	**Urban**	**5 years (2007-2011)**	**Ying Zhao, Chun-Yan Liu 赵莹,刘春艳 (2012). An analysis of the results of death monitoring of children under 5 years old in Hebei district, Tianjin city from 2006 to 2011* (2006-2011 年度天津市河北区 5 岁以下儿童死亡监测结果分析). Journal of Tianjin Medical University (天津医科大学学报). 18(2): 178-180**	**17,803**
**C185**	**Zhong R et al.**	**2012**	**Guangxi**	**1 city Baise**	**Urban and Rural**	**5 years (2006-2010)**	**Run Zhong, Ming-Gang Ban, et al. 钟润,班明刚,等 (2012). An investigation on neonatal deaths in Baise city from 2006 to 2010* (2006～2010 年百色市新生儿死亡情况调查). Youjiang Medical Journal (右江医学). 40(1): 107-108**	**254,269**

**C186**	**Zhou LK**	**2012**	**Yunnan**	**1 county Shizong**	**Urban and Rural**	**5 years (2007-2011)**	**Li-Kun Zhou 周丽坤 (2012). Monitoring and analysis of death factors of children under 5 years old in Shizong county in recent 5 years* (师宗县近五年 5 岁以下儿童死亡因素监测与分析). Seek Medical and Ask the Medicine (求医问药(下半月)). 10(12): 3**	**31,623**
**C187**	**Zhou YF**	**2012**	**Jiangsu**	**1 district in Yangzhou city**	**Urban and Rural**	**5 years (2007-2011)**	**Yan-Fen Zhou 周艳芬 (2012). An analysis of death monitoring of children under 5 years old in Jiangdu district, Yangzhou city from 2007 to 2011* (扬州市江都区 2007～2011 年 5 岁以下儿童死亡监测情况分析). J Huaihai Med (淮海医药). 30(3): 248-250**	**35,340**
**C188**	**Zhu XY et al.**	**2012**	**Zhejiang**	**39 towns and streets in Quzhou city**	**Urban and Rural**	**5 years (2006-2010)**	**Xiao-Yan Zhu, Cheng-Yin Huang 朱晓燕,黄诚茵 (2012). An analysis of death monitoring result of children under 5 years old from 2006 to 2010* (2006～2010 年 5 岁以下儿童死亡监测结果分析).**	**112,793**

							**Maternal & Child Health Care of China (中国妇幼保健). 27(13): 1994-1995**	
**C189**	**Zhuang XW et al.**	**2012**	**Shandong**	**1 county Junan**	**Urban and Rural**	**1 year (2010)**	**Xu-Wei Zhuang, Shu-Qin Ming 庄绪伟,明淑芹 (2012). Analysis of the monitoring data of the death of children under 5 years old in Ju'nan county in 2010 (莒南县 2010 年 5 岁以下儿童死亡监测分析). Journal of Shandong Medical College (山东医学高等专科学校学报). 34(4): 307-308**	**10,617**
**C190**	**Bai RS et al.**	**2013**	**Ningxia**	**1 county Yanchi**	**Urban and Rural**	**10 years (2003-2012)**	**Yong-Sheng Bai, Yan-Feng Liu, et al. 白永胜,刘雁峰,等 (2013). An analysis of death monitoring of children under 5 years old in Yanchi county from 2003 to 2012* (盐池县 2003-2012 年 5 岁以下儿童死亡监测分析). Journal of Ningxia Medical University (宁夏医科大学学报). 35(11): 1282-1284**	**18,843**
**C191**	**Cai RZ et al.**	**2013**	**Shanghai**	**1 city Shanghai**	**Urban and Rural**	**1 year (2011)**	**Ren-Zhi Cai, Hui-Ting Yu, et al. 蔡任之,虞慧婷,**	**203,821**

							等 (2013). Analysis on death for children under 5 in Shanghai registered population and floating population in 2011 (上海市 2011 年户籍及非户籍人口 5 岁以下儿童死亡分析). Asia-Pacific Traditional Medicine (亚太传统医药). 9(9): 205-207	
C192	Cao FL	2013	Hubei	1 city Yidu	Urban and Rural	5 years (2008-2012)	Fang-Lan Cao 曹方兰 (2013). Death tendency of children under 5 years old in Yidu city, Hubei province from 2008 to 2012* (湖北宜都市 2008-2012 年 5 岁以下儿童死亡趋势). J of Pub Health and Prev Med (公共卫生与预防医学). 24(5): 120-121	14,689
C193	Chen SY et al.	2013	Fujian	1 district Tongan in Xiamen city	Urban	5 years (09/2005-10/2009)	Su-Yun Chen, Xu Zhao 陈素云,赵旭 (2013). An analysis of deaths of children under 5 years old in Tongan district, Xiamen city from 2005 to 2009* (厦门市同安区 2005～2009 年 5 岁以下儿	19,475

							**童死亡分析). Maternal & Child Health Care of China (中国妇幼保健). 28(4): 637-639**	
**C194**	**Dong SH**	**2013**	**Anhui**	**citi zones in Bengbu city**	**Urban**	**9 years (2003-2011)**	**Su-Hua Dong 董素华 (2013). Analysis on cause of death of children under 5 years old in Bengbu city from 2003 to 2011 (2003 年至 2011 年蚌埠市 5 岁以下儿童死亡率及死亡原因分析). Anhui Medical Journal (安徽医学). 34(8): 1233-1236**	**59,948**
**C195**	**Fang HY et al.**	**2013**	**Anhui**	**urban areas in Tongling city**	**Urban**	**7 years (01/10/2006-30/09/2012)**	**Hong-Ying Fang, Li-Li Zhu, et al. 方红英,朱莉莉,等 (2013). Analysis of minotoring results of death for the children under 5 years old in Tongling (铜陵市 5 岁以下儿童死亡监测结果分析). CJCHC (中国儿童保健杂志). 21(8): 878-880**	**18,380**
**C196**	**Fang M**	**2013**	**Jiangsu**	**1 district Wujin in Changzhou city**	**Urban and Rural**	**3 years (2010-2012)**	**Ming Fang 方明 (2013). Death monitoring of children under 5 years old in Wujin district, Changzhou city from 2010 to 2012* (常州市武进区**	**41,132**

							2010—2012 年 5 岁以下儿童死亡监测). Jiangsu Health Care (江苏卫生保健). 15(6): 48-49	
C197	Feng HX	2013	Shandong	1 district Linzi in Zibo city	Urban	13 years (2000-2012)	Hai-Xia Feng 冯海霞 (2013). An analysis of death monitoring results of children under 5 years old in Linzi district, Zibo city of Shandong province from 2000 to 2012* (山东省淄博市临淄区 2000-2012 年 5 岁以下儿童死亡监测结果分析). Chindren's Health Academic Exchange Conference (2013 山东省儿童保健学术交流会). (4):	58,732
C198	Fu HD et al.	2013	Hubei	1 city Xiaogan	Urban and Rural	5 years (10/2006-09/2011)	Han-Dong Fu, Min Lu, et al. 付汉东,陆敏,等 (2013). Analysis of the causes of death among children under 5 years old in Xiaogan city from 2007 to 2011 (孝感市 2007—2011 年 5 岁以下儿童死亡原因调查分析). Chinese General Practice (中国全科医学). 16(1C): 286-288	214,857

**C199**	**Gao JH et al.**	**2013**	**Ningxia**	**1 county Haiyuan**	**Urban and Rural**	**3 years (2008-2010)**	**Jin-Hua Gao, Ning Li, et al. 高进华,李宁,等 (2013). An analysis of deaths of children under 5 years old from 2008 to 2010* (2008～2010 年 5 岁以下儿童死亡情况分析). Maternal & Child Health Care of China (中国妇幼保健). 28(13): 2026-2028**	**23,487**
**C200**	**Guo YX et al.**	**2013**	**Ningxia**	**1 district Jinfeng in Yinchuan city**	**Urban**	**8 years (2005-2012)**	**Yin-Xia Guo, Hui-Zhi Li, et al. 郭银霞,李惠芝,等 (2013). An analysis of death causes of children under 5 years old in Jinfeng district, Yinchuan city from 2005 to 2012* (银川市金凤区 2005-2012 年 5 岁以下儿童死亡原因分析). Ningxia Med J (宁夏医学杂志). 35(10): 984-985**	**13,946**
**C201**	**Hu XZ et al.**	**2013**	**Hubei**	**1 district Zengdu in Suizhou city**	**Urban and Rural**	**5 years (2008-2012)**	**Xiu-Zhen Hu, Tao Jiang, et al. 胡秀珍,蒋涛,等 (2013). The analysis of death situation of children under five years in Zengdouqu district of Suizhou from 2008 to 2012 (随州市曾都区 2008—2012 年 5 岁以下儿**	**71,902**

							童死亡情况分析). Chinese Primary Health Care (中国初级卫生保健). 27(8): 57-59	
**C202**	**Jin HY et al.**	**2013**	**Jilin**	**8 counties in Yanbian city**	**Urban and Rural**	**4 years (2009-2012)**	**Hai-Yu Jin, Jing-Hua Quan 金海玉,全京花 (2013). Death cause analysis of under five years old children from 2009-2012 in Yanbian area (2009～2012 年延边地区 5 岁以下儿童死亡原因分析). China Prac Med (中国实用医药). 8(7): 263-264**	**55,627**
**C203**	**Kou XJ et al.**	**2013**	**Shandong**	**1 county Shouguang**	**Urban and Rural**	**3 years (2009-2011)**	**Xiao-Juan Kou, Zhong-Jian Fu, et al. 寇晓娟,付中建,等 (2013). An analysis of deaths of children under 5 years old in Shouguang city from 2009 to 2011* (2009～2011 年寿光市 5 岁以下儿童死亡情况分析). Prev Med Trib (预防医学论坛). 19(10): 795-796**	**28,655**
**C204**	**Li JQ**	**2013**	**Shanxi**	**1 district Yingze in Taiyuan city**	**Urban**	**5 years (2006-2010)**	**Jun-Qin Li 李俊琴 (2013). Analysis of the death rate of children under 5 in Yingze district, Taiyuan**	**17,053**

							during 2006~2010 (太原市迎泽区 2006~2010 年 5 岁以下儿童死亡情况分析). Journal of Shanxi Medical College for Continuing Education (山西职工医学院学报). 23(5): 42-44	
C205	Liu L et al.	2013	Heilongjiang	8 districts and 10 counties in Haerbin city	Urban and Rural	11 years (2000-2010)	Li Liu, Chun-Hua Wang, et al. 刘丽,王春华,等 (2013). Trend analysis on infantile death in Harbin city from 2000 to 2010 (2000～2010 年哈尔滨市婴儿死亡趋势分析). Maternal & Child Health Care of China (中国妇幼保健). 28(5): 785-788	723,873
C206	Liu SM	2013	Guangxi	1 county Fuchuan	Urban and Rural	5 years (26/12/2009-25/12/2013)	Shi-Min Liu 刘世敏 (2013). An analysis of death causes of children under 5 years old in Fuchuan county, China from 2009-2013* (富川县 2009-2013 年五岁以下儿童死亡原因分析). World Health Digest (中外健康文摘). (51): 53-54	25,468
C207	Long YZ et al.	2013	Jiangxi	1 city Nanchang	Urban and Rural	5 years (2008-2012)	Yuan-Zhu Long, Chu-Yan Long, et al. 龙元珠,龙楚彦,等 (2013). An	57,865

							analysis of the monitoring child death of children under 5 years old in Nanchang area, China from 2008 to 2012* (2008-2012 年南昌地区 5 岁以下儿童死亡监测分析). Chinese Journal of Women and Children Health (中国妇幼卫生杂志). 4(5): 35-36	
C208	Luo KM et al.	2013	Yunnan	1 district Linxiang in Lincang city	Urban and Rural	10 years (2001-2010)	Kai-Min Luo, Yong-Zhong Wang 罗开敏,汪永忠 (2013). An analysis of death year report materials of children under 5 years old in Linxiang district, China from 2001 to 2010* (2001～2010 年临翔区 5 岁以下儿童死亡年报资料分析). Chinese Community Doctors (中国社区医师). 15(6): 374-375	28,854
C209	Lv T	2013	Liaoning	7 counties in Anshan city	Urban and Rural	5 years (2007-2011)	Tao Lu 吕涛 (2013). Monitoring results of children mortality under 5 years old in Anshan city from 2007 to 2011 (2007 至 2011 年鞍山市 5 岁以	131,797

							下儿童死亡监测结果分析). Chinese Journal of Women and Child Health Research (中国妇幼健康研究). 24(2): 149-151	
C210	Ma M et al.	2013	Gansu	1 district Chengguan in Lanzhou city	Urban and Rural	6 years (2005-2010)	Ming Ma, Shu-Wen Zhang, et al. 马铭,张淑文,等 (2013). Analysis on death causes of children under 5 years old in Chengguan district of Lanzhou city from 2005 to 2010 (2005～2010 年兰州市城关区 5 岁以下儿童死因分析). Maternal & Child Health Care of China (中国妇幼保健). 28(3): 458-461	55,868
C211	Ni YF et al.	2013	Jiangsu	9 districts and counties in Nantong city	Urban and Rural	7 years (2005-2011)	Yu-Fei Ni, Ya-Bing Lu, et al. 倪钰飞,吕亚兵,等 (2013). An analysis of monitoring deaths of children under 5 years old in Nantong city, China from 2005 to 2011* (南通市 2005～2011 年 5 岁以下儿童死亡监测分析). Maternal & Child Health Care of China (中国妇幼保健). 28(27): 4511-4514	333,299

**C212**	**Peng HL et al.**	**2013**	**Shaanxi**	**2 districts and counties in Hanzhong city**	**Urban and Rural**	**11 years (2000-2010)**	**Hai-Ling Peng, Qian Bai, et al. 彭海玲,白倩,等 (2013). Contrastive analysis of the death of children under 5 years old in urban and rural areas of Hanzhong (汉中市农村与城区 5 岁以下儿童死亡对比分析). Chinese Journal of Women and Child Health Research (中国妇幼健康研究). 24(3): 293-295**	**91,482**
**C213**	**Qin WX et al.**	**2013**	**Fujian**	**1 city Xiamen**	**Urban and Rural**	**5 years (2007-2011)**	**Wei-Xia Qin, Han-Song Zhu, et al. 秦维霞,祝寒松,等 (2013). Epidemic feature analysis of children under-five mortality cause in Xiamen city (2007-2011 年厦门市 5 岁以下儿童死因流行特征分析). CJCHC (中国儿童保健杂志). 21(6): 654-657**	**155,932**
**C214**	**Shi WX**	**2013**	**Shanghai**	**1 district Fengxian in Shanghai**	**Urban**	**5 years (2008-2012)**	**Wei-Xing Shi 施卫兴 (2013). An analysis of deaths of children under 5 years old in Fengxian district, Shanghai from 2008 to 2012* (2008-2012**	**33,364**

							年上海市奉贤区 5 岁以下儿童死亡分析). hanghai Medical & Pharmaceutical Journal (上海医药). 34(8): 52-54	
**C215**	**Tang ZQ**	**2013**	**Shandong**	**1 city Pingdu**	**Urban and Rural**	**4 years (2009-2012)**	**Shao-Qiu Tang 唐召秋 (2013). An analysis of deaths of children under 5 years old in Pingdu city of Shandong province from 2009 to 2012* (山东省平度市 2009～2012 年 5 岁以下儿童死亡情况分析). Capital Medicine (首都医药). (14): 37**	**47,508**
**C216**	**Teng ZX**	**2013**	**Sichuan**	**1 city Huaying**	**Urban and Rural**	**5 years (2006-2010)**	**Chao-Xia Teng 滕朝霞 (2013). An analysis of death monitoring results of children under 5 years old in Huaying city from 2006 to 2010* (华蓥市 2006 年～2010 年 5 岁以下儿童死亡监测结果分析). Jilin Medical Journal (吉林医学). 34(22): 4489-4490**	**15,035**
**C217**	**Wang YX et al.**	**2013**	**Yunnan**	**1 district and 8 counties in Yuxi city**	**Urban and Rural**	**5 years (2008-2012)**	**Yun-Xia Wang, Mei-Ling Liu, et al. 王云霞,刘美玲,等 (2013). An analysis of neonatal monitoring**	**106,442**

							**deaths in Yuxi city from 2008 to 2012* (玉溪市 2008～2012 年新生儿死亡监测情况分析). Medicine and Pharmacy of Yunnan (云南医药). 34(5): 432-433**	
**C218**	**Wang Z et al.**	**2013**	**Hunan**	**1 city Hengyang**	**Urban and Rural**	**6 years (2006-2011)**	**Zhen Wang, Yong-Hong Duan 王珍,段永红 (2013). Analysis of children under 5 years of age mortality of Hengyang city from 2006 to 2011 (衡阳市 2006-2011 年 5 岁以下儿童死亡情况分析). Chinese Journal of Women and Children Health (中国妇幼卫生杂志). 4(1): 10-11**	**468,921**
**C219**	**Wang ZD et al.**	**2013**	**Shandong**	**1 city Zibo**	**Urban and Rural**	**11 years (2000-2010)**	**Zhong-De Wang, Qian-Yun Wang, et al. 王忠德,王茜云,等 (2013). Analysis on mortality surveillance among children aged under 5 years, Zibo city (2000～2010 年淄博市 5 岁以下儿童死亡监测资料分析). Prev Med Trib (预防医学论坛). 19(2): 125-127**	**416,542**
**C220**	**Wei HZ et al.**	**2013**	**Guangdong**	**1 street Shajing in Shenzhen city**	**Urban**	**5 years (2007-2011)**	**Huang-Zhong Wei, Guang-Ying Zhao, et al.**	**42,749**

							魏煌忠,赵广英,等 (2013). An analysis of deaths of children under 5 years old in one street of Shenzhen city from 2007 to 2011* (深圳某街道 2007-2011 年 5 岁以下儿童死亡情况分析). J of Pub Health and Prev Med (公共卫生与预防医学). 24(1): 115-116	
C221	Wu JX et al.	2013	Zhejiang	1 city Yuyao	Urban and Rural	5 years (01/10/2007-30/09/2012)	Jin-Xi Wu, Su Huang 吴金曦,黄素 (2013). An analysis of death causes of children under 5 years old in Yuyao area, China from 2008 to 2012* (2008 年～2012 年余姚地区 5 岁以下儿童死亡原因分析). Chinese Journal of Birth Health & Heredity (中国优生与遗传杂志). 21(10): 124-125	24,166
C222	Wu M	2013	Sichuan	11 districts and counties in Leshan city	Urban and Rural	6 years (2005-2010)	Min Wu 吴敏 (2013). Analysis of the causes of child death under five years of age in Leshan city from 2005 to 2010 (乐山市 2005 年-2010 年 5 岁以下儿童死亡原因分析). West China Medical Journal	153,923

							(华西医学). 28(5): 761-763	
**C223**	**Wu WS**	**2013**	**Zhejiang**	**1 county Sanmen**	**Urban and Rural**	**5 years (2007-2011)**	**Wei-Shuang Wu 吴伟爽 (2013). An analysis of death causes of children under 5 years old in Sanmen county, China from 2007 to 2011* (三门县 2007-2011 年 5 岁以下儿童死因分析). Chinese Journal of Rural Medicine and Pharmacy (中国乡村医药). 20(16): 53-54**	**23,659**
**C224**	**Wu XW**	**2013**	**Henan**	**6 districts and 6 counties in Zhengzhou city**	**Urban and Rural**	**3 years (2010-2012)**	**Xiao-Wen Wu 武晓雯 (2013). An analysis of death causes of children under 5 years old in Zhengzhou city from 2010 to 2012* (2010~2012 年郑州市 5 岁以下儿童死亡原因分析). Journal of Henan Medical College for Staff and Workers (河南职工医学院学报). 25(6): 715-716**	**336,018**
**C225**	**Xiang XS et al.**	**2013**	**Shaanxi**	**13 districts and counties in Xian city**	**Urban and Rural**	**3 years (2010-2012)**	**Xiao-Shu Xiang, Shui-Ping Zhang, et al. 相晓姝, 张水平, 等 (2013). Analysis of neonatal death review in Xi'an city from**	**189,472**

							2010 to 2012 (2010 至 2012 年西安市新生儿死亡评审分析). Chinese Journal of Women and Child Health Research (中国妇幼健康研究). 24(4): 478-481	
C226	Xiong XY	2013	Hubei	1 city Enshi	Urban and Rural	3 years (2010-2012)	Xiao-Yan Xiong 熊晓妍 (2013). An analysis of death causes of children under 5 years old in Enshi county, China from 2010 to 2012* (恩施市 2010-2012 年 5 岁以下儿童死亡原因分析). Health Horizon (健康大视野：医学版). 21(9): 873-874	20,872
C227	Yan ZR	2013	Chongqing	1 district Beibei in Chongqing city	Urban	6 years (01/10/2005-30/09/2011)	Ze-Rong Yan 晏泽容 (2013). Investagetion and analysis of death causes of children under 5 years old in Beibei district, China from 2006 to 2011* (2006～2011 年北碚区 5 岁以下儿童死因调查及分析). Chin J Mod Drug Appl (中国现代药物应用). 7(2): 130-132	24,645
C228	Yang HF et al.	2013	Jiangsu	1 city Nanjing	Urban and Rural	5 years (2007-2011)	Hua-Feng Yang, Xu-Peng Chen, et al. 杨华凤,陈旭	264,706

							鹏,等 (2013). Analysis on the death situation of the children under 5 years in Nanjing from 2007 to 2011 (南京市 2007～2011 年 5 岁以下儿童死亡情况分析). Maternal & Child Health Care of China (中国妇幼保健). 28(3): 442-444	
C229	Yang RH	2013	Sichuan	1 city Chongzhou	Urban and Rural	10 years (2001-2010)	Rong-Hui Yang 杨荣惠 (2013). Analysis of the monitoring results on children death under the age of five in Chongzhou city between 2001 and 2010 (崇州市 2001~2010 年 5 岁以下儿童死亡监测结果分析). Morden Preventive Medicine (现代预防医学). 40(20): 3768-3770	38,654
C230	Yin GZ et al.	2013	Anhui	1 city Hefei	Urban and Rural	5 years (2006-2010)	Gang-Zhu Yin, Su-Lin Fu, et al. 殷刚柱,傅苏林,等 (2013). An analysis of monitoring deaths of children under 5 years old in Hefei city, China from 2006 to 2010* (2006～2010 年合肥市 5 岁以下儿	209,282

							童死亡监测分析). Maternal & Child Health Care of China (中国妇幼保健). 28(17): 2679-2681	
C231	Yu CE	2013	Guizhou	1 county Dushan	Urban and Rural	5 years (01/10/2007-30/09/2012)	Chun-E Yu 余春娥 (2013). Analysis of the investigation result of death causes of children under 5 years old in Dushan county, China from 2008 to 2012* (独山县 2008-2012 年 5 岁以下儿童死亡原因调查结果分析). China Hwalth Care & nutrition (中国保健营养（中旬刊）). (12): 35-36	17,869
C232	Yu QX	2013	Jiangsu	1 district Huishan in Wuxi city	Urban	10 years (2001-2010)	Qiu-Xia Yu 俞秋霞 (2013). Analysis of the death cause of children less than 5 years old in Huishan district of Wuxi (江苏省无锡市惠山区 5 岁以下儿童十年死亡原因分析). Chin J Prim Med Pharm (中国基层医药). 20(21): 3269-3271	40,298
C233	Zhan YX et al.	2013	Fujian	1 city Putian	Urban and Rural	6 years (2007-2012)	Yu-Xia Zhan, Yi-Jun Ruan, et al. 詹玉霞,阮一君,等 (2013). Analysis and countermeasures of	242,968

							**deaths of children under 5 years old in Putian city from 2007 to 2012* (莆田市 2007-2012 年 5 岁以下儿童死亡分析与对策). Strait J Prev Med (海峡预防医学杂志). 19(4): 76-77**	
**C234**	**Zhang MJ**	**2013**	**Neimenggu**	**22 towns in Balizuoqi**	**Rural**	**5 years (01/10/2007-30/09/2012)**	**Min-Jie Zhang 张敏杰 (2013). An analysis of monitoring deaths of children under 5 years old in Balinzuoqi, China from 2008 to 2012* (巴林左旗 2008--2012 年 5 岁以下儿童死亡监测分析). China Health Care & Nutrition (中国保健营养（上旬刊）). (12): 7378-7379**	**15,005**
**C235**	**Zhao LP**	**2013**	**Shaanxi**	**11 districts and counties in Weinan city**	**Urban and Rural**	**5 years (2006-2010)**	**Li-Ping Zhao, Min-Hui Cao 赵丽萍, 曹敏辉 (2013). Analysis of death cause of children under age 5 from 2006 to 2010 in Weinan (渭南市 2006 至 2010 年 5 岁以下儿童死因分析). Chinese Journal of Women and Child Health Research (中国妇幼健康研究). 24(1): 10-12**	**45,302**
**C236**	**Zhu JL**	**2013**	**Gansu**	**4 counties, 1 district**	**Urban and**	**5 years (01/10/2006-**	**Jian-Ling Zhu, Zhan-**	**41,092**

	**et al.**			**and 2 cities in Jiuquan city**	**Rural**	**30/09/2011)**	**Shun Cui 朱建玲,崔占顺 (2013). Tendency analysis of mortality rates of children under 5 years old in Jiuquan city, China from 2007 to 2011* (2007～2011 年酒泉市 5 岁以下儿童死亡率变化趋势探讨). Maternal & Child Health Care of China (中国妇幼保健). 28(12): 1906-1908**	
**C237**	**Bu FL**	**2014**	**Shandong**	**1 county Pingyi**	**Urban and Rural**	**7 years (2006-2012)**	**Fan-Ling Bu 卜凡玲 (2014). Analysis and intervention countermeasures of death causes of perinatal infants and children under 5 years old in Pingyi county from 2006 to 2012* (平邑县 2006～2012 年围产儿和 5 岁以下儿童死亡原因分析及干预对策). Contemporary Medicine (当代医学). 20(19): 160-162**	**67,352**
**C238**	**Chen YX et al.**	**2014**	**Hainan**	**6 towns and 2 counties in Sanya sity**	**Urban and Rural**	**13 years (2000-2012)**	**Yan-Xia Chen, Chuan-Bi Chen, et al. 陈艳霞,陈川碧,等 (2014). Tendency analysis and preventive countermeasures of**	**71,117**

							**deaths of children under 5 years old in Sanya city, China from 2000 to 2012* (三亚市 2000～2012 年 5 岁以下儿童死亡变化趋势分析及防治对策). Maternal & Child Health Care of China (中国妇幼保健). 29(8): 1156-1158**	
**C239**	**Deng YL**	**2014**	**Guangxi**	**1 county Quanzhou**	**Urban and Rural**	**5 years (2008-2012)**	**You-Ling Deng 邓佑玲 (2014). Analysis of the result of mornitoring neonatal deaths in one county, China from 2008 to 2012* (某县 2008 年~2012 年新生儿死亡监测的结果分析). Guide of China Medicine (中国医药指南). 12(5): 59-60**	**54,023**
**C240**	**Feng GF et al.**	**2014**	**Guangdong**	**1 district Liwan in Guangzhou city**	**Urban**	**5 years (01/10/2008-30/09/2013)**	**Gui-Fen Feng, Bao-Shan Gan, et al. 冯桂芬,甘宝姗, 等 (2014). Death cause analysis and corresponding intervention measures for children under 5 years old in Guangzhou city Liwan district (广州市荔湾区 5 岁以下儿童死因分析及相关干预对策). China**	**31,982**

							**Morden Doctor (中国现代医生). 52(9): 126-129**	
**C241**	**Gao JH et al.**	**2014**	**Guangdong**	**24 towns and cities in Zhongshan city**	**Urban and Rural**	**10 years (2003-2012)**	**Jian-Hui Gao, Yu-Shao Liu, et al. 高建慧,刘玉韶, 等 (2014). Analysis of the causes and intervention strategy of neonatal death in Zhongshan city from 2003 to 2012 (中山市 2003-2012 年新生儿死亡分析及干预对策). CJCHC (中国儿童保健杂志). 22(10): 1101-1103**	**453,925**
**C242**	**Kang HQ**	**2014**	**Ningxia**	**1 county Longde**	**Urban and Rural**	**5 years (2008-2012)**	**Hui-Qin Kang 康慧琴 (2014). An analysis of death causes of children under 5 years old in Longde county from 2008 to 2012* (隆德县 2008-2012 年 5 岁以下儿童死因分析). Journal of Ningxia Medical University (宁夏医科大学学报). 36(11): 1284-1285**	**5,418**
**C243**	**Li HH et al.**	**2014**	**Zhejiang**	**2 districts and 3 counties in Huzhou city**	**Urban and Rural**	**5 years (2008-2012)**	**Hai-Hua Li, Yan-Hong He, et al. 李海华,何艳宏, 等 (2014). An analysis of death causes of children under 5 years old in Huzhou city, China from**	**96,403**

							**2008 to 2012* (2008~2012年湖州市5岁以下儿童死亡原因分析). Maternal & Child Health Care of China (中国妇幼保健). 29(34): 5610-5612**	
**C244**	**Li YH et al.**	**2014**	**Hebei**	**1 county Chengde**	**Urban and Rural**	**4 years (2010-2013)**	**Yan-Hui Li, Chun-Yan Niu 李艳辉,牛春艳 (2014). An analysis of 144 death cases of children under 5 years old in Chengde county, China* (承德县144例5岁以下儿童死亡分析). Chinese Journal of Ethnomedicine and ethnopharmacy (中国民族民间医药). 23(8): 110**	**18,021**
**C245**	**Li Y et al.**	**2014**	**Hubei**	**1 district Wuchang in Wuhan city**	**Urban**	**7 years (2005-2011)**	**Ying Li, Yuan Lu 李莹,卢媛 (2014). Analysis of death causes and countermeasures of children under 5 years old in Wuchang district, Wuhan city from 2005 to 2011* (武汉市武昌区2005~2011年5岁以下儿童死因及干预措施分析). Maternal & Child Health Care of China (中国妇幼保健). 29(16): 2475-2478**	**39,231**

**C246**	**Ling XH**	**2014**	**Jiangsu**	**1 district Liuhe in Nanjing city**	**Urban and Rural**	**10 years (2004-2013)**	**Xu-Hong Ling 凌序红 (2014). An analysis of deaths of children under five years old in Liuhe district from 2004 to 2013* (六合区 2004~2013 年 5 岁以下儿童死亡分析). Medical Information (医学信息). 27(12): 353**	**64,812**
**C247**	**Liu FY et al.**	**2014**	**Liaoning**	**1 district Dongling in Shenyang city**	**Urban**	**5 years (2009-2013)**	**Fang-Yu Liu, Juan Lu 刘芳妤,路娟 (2014). An analysis of death records of children under 5 years old in Dongling district, Shenyang* (沈阳市东陵区＜5 岁儿童死亡资料分析). hinese Practical Journal of Rural Doctor (中国实用乡村医生杂志). 21(4): 8-9**	**16,576**
**C248**	**Liu HM**	**2014**	**Guizhou**	**1 city Fuquan**	**Urban and Rural**	**5 years (2006-2010)**	**Hong-Mei Liu 刘红梅 (2014). Discussion on multi death infulencing factors of children under 5 years old* (影响 5 岁以下儿童死亡多因素探讨). Chinese Baby (母婴世界). (14): 87-88**	**15,504**
**C249**	**Liu MX et al.**	**2014**	**Hubei**	**1 county Xunxi**	**Urban and Rural**	**10 years (2004-2013)**	**Ming-Xing Liu, Xue Sun 刘明星,孙雪 (2014). An**	**50,388**

							analysis of death causes of children under 5 years old in Xunxi county, China from 2004 to 2013* (郧西县 2004-2013 年 5 岁以下儿童死亡原因分析). China Medical Equipment (中国医学装备). 11(): 79-80	
C250	Liu SY	2014	Guizhou	1 district in Zunyi city	Urban and Rural	4 years (2010-2013)	Shi-Ying Liu 刘世英 (2014). An analysis of monitoring deaths of children under 5 years old* (5 岁以下儿童死因监测分析). Health Care Today (现代养生). (12): 56	23,592
C251	Lu HC	2014	Guangxi	1 county Lingshan	Urban and Rural	1 year (2013)	Hai-Chan Lu 陆海婵 (2014). An analysis of monitoring deaths of children under 5 years old in Lingshan county in 2013* (灵山县 2013 年 5 岁以下儿童死亡监测分析). Yiayao Qianyan (医药前沿). (22): 386-387	26,755
C252	Luo XF	2014	Henan	1 district in Nanyang city	Urban and Rural	2 years (2010-2011)	Xiao-Fan Luo 罗晓帆 (2014). Review of death of children below 5 years old: an analysis of 191	18,736

							**cases (191 例 5 岁以下儿童死亡评审分析). Henan J Prev Med (河南预防医学杂志). 25(5): 341-343**	
**C253**	**Lv GQ**	**2014**	**Hunan**	**3 districts and 9 counties in Shaoyang city**	**Urban and Rural**	**6 years (2008-2013)**	**Guo-Qiang Lu 吕国强 (2014). An analysis of death records of children under five years old in Shaoyang city from 2008 to 2013* (邵阳市 2008 年～2013 年 0～4 岁儿童死亡资料分析). Medical Information (医学信息). 27(5): 126-127**	**522,072**
**C254**	**Meng ZL**	**2014**	**Tianjin**	**1 district Wuqing in Tianjin city**	**Urban and Rural**	**3 years (2010-2012)**	**Shao-Ling Meng 孟召苓 (2014). An analysis of monitoring deaths of children under 5 years old in Wuqing district, China from 2010 to 2012* (武清区 2010~2012 年 5 岁以下儿童死亡监测分析). Chinese Journal of Urban and Rural Enterprise Hygiene (中国城乡企业卫生). (2): 144-146**	**23,264**
**C255**	**Nong BY et al.**	**2014**	**Yunnan**	**1 county Guangnan**	**Urban and Rural**	**5 years (2008-2012)**	**Bing-Yu Nong, Bin Yan, et al. 侬炳毓,严斌,等 (2014). An analysis of deaths of children under 5**	**56,846**

							**years ofl in Guangnan County, China from 2008 to 2012* (2008-2012 年广南县 5 岁以下儿童死亡情况分析). Chinese-foreign Women's Health (中外女性健康（下半月）). (1): 90-91**	
**C256**	**Qiao YG et al.**	**2014**	**Anhui**	**1 district Shushan in Hefei city**	**Urban and Rural**	**5 years (2008-2012)**	**Yi-Ge Qiao, Rong Qu, et al. 乔艺阁,曲荣,等 (2014). Analysis of mortality and death causes of children under five years old in Shushan district of Hefei city from 2008 to 2012 (合肥市蜀山区 2008 至 2012 年 5 岁以下儿童死亡率及死亡原因分析). Anhui Medical Journal (安徽医学). 35(2): 226-229**	**18,971**
**C257**	**Rong Z**	**2014**	**Anhui**	**1 district Yingdong in Fuyang city**	**Urban and Rural**	**3 years (2011-2013)**	**Zheng Rong 荣征 (2014). An analysis of death monitoring results of children under 5 years old in Yingdong district from 2011 to 2013* (颍东区 2011～2013 年 5 岁以下儿童死亡监测结果分析). Anhui J Prev Med (安徽预防医学杂志). 20(6):**	**21,948**

							477-478	
C258	Shen XZ et al.	2014	Zhejiang	1 district Yuhang in Hangzhou city	Urban and Rural	4 years (10/2008-03/2012)	Xue-Zhen Shen, Chun-Yan Zhang 沈雪珍,章春燕 (2014). Analysis and preventive measure management of deaths of children under 5 years old in Yuhang district, China* (余杭区 5 岁以下儿童死亡情况分析与预防措施管理). Chinese Rural Health Service Administration (中国农村卫生事业管理). 34(4): 450-452	33,149
C259	Tang C	2014	Guizhou	1 county Qianxi	Urban and Rural	5 years (2009-2013)	Chun Tang 唐春 (2014). An analysis of monitoring deaths of children under 5 years old in Qianxi county, China from 2009 to 2013* (黔西县 2009--2013 年 5 岁以下儿童死亡监测分析). China Health Care & Nutrition (中国保健营养（下旬刊）). (5): 2895	50,111
C260	Tao CY et al.	2014	Jiangsu	1 city Haimen	Urban and Rural	10 years (2003-2012)	Chang-Yu Tao, Zhuo-Jian Ni, et al. 陶长余,倪倬健,等 (2014). Study on death of children under five	75,003

							years old and the trend in Haimen from 2003 to 2012 (海门市 2003~2012 年 5 岁以下儿童死亡状况及趋势研究). Maternal & Child Health Care of China (中国妇幼保健). 29(35): 5812-5816	
C261	Wang HL	2014	Hebei	1 city Chengde	Urban and Rural	5 years (2009-2013)	Hong-Lei Wang 王宏蕾 (2014). An analysis of death monitoring results of children under 5 years old in Chengde city from 2009 to 2013* (2009 年-2013 年承德市 5 岁以下儿童死亡监测结果分析). Medicine and Health Care (医药与保健). 22(5): 95-96	195,384
C262	Wang J	2014	Liaoning	1 city Fengcheng	Urban and Rural	5 years (2009-2013)	Jin Wang 王瑾 (2014). An analysis of deaths of children under 5 years old in Fengcheng city, China from 2009 to 2013* (凤城市 2009-2013 年度 5 岁以下儿童死亡分析). World Health Digest (中外健康文摘). (7): 205	16,811
C263	Wang YX et al.	2014	Guangdong	1 city Jiangmen	Urban and Rural	11 years (2003-2013)	Ying-Xiang Wang, Shu-Kun Zhang, et al. 王英翔, 张淑琨,等 (2014). An	121,068

							**analysis of monitoring death results and interventions of children under 5 years old in Jiangmen area, China from 2003 to 2013* (2003 年～2013 年江门地区 5 岁以下儿童死亡监测结果及干预措施分析). Chinese Journal of Birth Health & Heredity (中国优生与遗传杂志). 22(12): 126-127**	
**C264**	**Wang CP**	**2014**	**Anhui**	**1 county Changfeng**	**Urban and Rural**	**5 years (2009-2013)**	**Chang-Ping Wang 王长平 (2014). The analysis of the death rate of children under the 5 years old from 2009 to 2013, Changfeng county (合肥市长丰县 2009-2013 年 5 岁以下儿童死亡分析). Chinese Journal of Women and Children Health (中国妇幼卫生杂志). 5(6): 57-59**	**35,304**
**C265**	**Wu H**	**2014**	**Sichuan**	**1 district Cuiping in Yibin city**	**Urban and Rural**	**10 years (2001-2010)**	**Hong Wu 吴虹 (2014). An analysis of deaths of children under 5 years old in Cuiping district, Yibin city from 2001 to 2010* (宜宾市翠屏区 2001 年--**	**67,888**

							2010 年 5 岁以下儿童死亡分析). China Health Care & Nutrition (中国保健营养（上旬刊）). (1): 479-480	
C266	Wu YH et al.	2014	Guangdong	1 city Taishan	Urban and Rural	6 years (2007-2012)	Ying-Hua Wu, Wei-Zhan Chen, et al. 伍颖华,陈伟湛,等 (2014). An analysis of deaths of children under 5 years old in Taishan city, Guangdong province from 2007 to 2012* (广东台山市 2007-2012 年 5 岁以下儿童死亡情况分析). J of Pub Health and Prev Med (公共卫生与预防医学). 25(1): 91-93	53,568
C267	Yan SJ et al.	2014	Beijing	1 city Beijing	Urban and Rural	10 years (2003-2012)	Shu-Juan Yan, Xue-Na Zhu 闫淑娟,朱雪娜 (2014). Analysis of mortality rate and causes of death among children under 5 years old in Beijing from 2003 to 2012 (2003-2012 年北京市 5 岁以下儿童死亡率和死亡原因分析). Chin J Prev Med (中华预防医学杂志). 48(6): 484-490	758,612

C268	Yang LP et al.	2014	Hubei	1 city Tianmen	Urban and Rural	4 years (2009-2012)	Li-Ping Yang, Li-Rong Tang 阳丽萍,唐丽蓉 (2014). Monitor and analysis of death in children under 5 years during 2009-2012 in Tianmen (天门市 2009-2012 年 5 岁以下儿童死亡监测分析). Morden Preventive Medicine (现代预防医学). 41(12): 2175-2176	58,919
C269	Yang GY et al.	2014	Shandong	1 city Zhangqiu	Urban and Rural	6 years (2008-2013)	Gui-Yun Yang, Jin-Ling Shan, et al. 杨桂芸,单金玲,等 (2014). An analysis of deaths of children under 5 years old in Zhangqiu city, China from 2008 to 2013* (2008～2013 年章丘市 5 岁以下儿童死亡情况分析). China Prac Med (中国实用医药). 9(32): 253-254	52,060
C270	Yao Y et al.	2014	Shanghai	1 district Minhang in Shanghai city	Urban	3 years (2010-2012)	Yi Yao, Shu-Rong Kang, et al. 姚亦,康淑蓉,等 (2014). Analysis of mionitoring results in the death of children below 5 years old in Minhang district of Shanghai from	59,026

							2010 to 2012 (2010—2012 年上海市闵行区 5 岁以下儿童死亡监测结果分析). Chinese Primary Health Care (中国初级卫生保健). 28(4): 40-42	
C271	Zhang C et al.	2014	Jiangsu	1 county Taicang in Suzhou city	Urban and Rural	10 years (01/10/2004-30/09/2013)	Cheng Zhang, Yong Xu 张诚, 徐勇 (2014). An analysis of monitoring deaths of children under 5 years old in Taicang city from 2004 to 2013* (2004-2013 年太仓市<5 岁儿童死亡监测分析). Jiangsu J Prev Med (江苏预防医学). 25(6): 84-85	58,963
C272	Zhang XM	2014	Jiangsu	1 zong Jiangning in Nanjing city	Urban and Rural	7 years (2005-2011)	Xiao-Mei Zhang 张晓媚 (2014). An analysis of deaths of children under 5 years old in Jiangning district, Nanjing city* (南京市江宁区 5 岁以下儿童死亡分析). Maternal & Child Health Care of China (中国妇幼保健). 29(1): 59-61	63,328
C273	Zhang XJ et al.	2014	Liaoning	Rural areas in Shenyang city	Rural	10 years (01/10/2002-30/09/2012)	Xue-Jiao Zhang, Li-Li Li, et al. 张雪娇,李荔荔,等 (2014). Trend analysis of mortality of children	272,249

							under 5 years in countryside of Shenyang from 2003 to 2012 (2003 至 2012 年沈阳市农村 5 岁以下儿童死亡趋势分析). Chinese Journal of Women and Child Health Research (中国妇幼健康研究). 25(2): 193-195	
C274	Zhang XJ et al.	2014	Liaoning	1 city Shenyang	Urban and Rural	5 years (01/10/2007-30/09/2012)	Xue-Jiao Zhang, Li-Li Li, et al. 张雪娇,李荔荔,等 (2014). Analysis of monitoring mortality result on children under 5 years old in Shenyang from 2008 to 2012 (沈阳市 2008-2012 年 5 岁以下儿童死亡监测结果分析). CJCHC (中国儿童保健杂志). 22(2): 216-218	260,964
C275	Zhao X	2014	Liaoning	1 city Liaoyang	Urban and Rural	5 years (01/10/2007-30/09/2012)	Xin Zhao 赵昕 (2014). Analysis on death of children under 5 years old from 2008-2012 in Liaoyang city (辽阳市 2008～2012 年 5 岁以下儿童死亡情况分析). China Medicine and Pharmacy (中国医药科学). 4(4): 87-89	59,344

**C276**	**Zhou HQ**	**2014**	**Yunnan**	**1 county Midu**	**Urban and Rural**	**7 years (2006-2012)**	**Hui-Qin Zhou 周慧琴 (2014). An analysis of death causes of children under five years old in Midu county from 2006 to 2012* (弥渡县 2006~2012 年五岁以下儿童死亡原因分析). Medical Information (医学信息). 27(1): 135-136**	**21,419**
**C277**	**Zhou J**	**2014**	**Jiangsu**	**1 city Yizheng**	**Urban and Rural**	**8 years (2006-2013)**	**Jing Zhou 周晶 (2014). Analysis of the death rate and cause of death among children under 5 years old in Yizheng city from 2006 to 2013 (仪征市 2006-2013 年 5 岁以下儿童死亡率及死亡原因分析). Chinese Journal of Women and Children Health (中国妇幼卫生杂志). 5(6): 60-63**	**32,042**
**C278**	**Zhou WL et al.**	**2014**	**Shanghai**	**1 district Changning in Shanghai city**	**Urban**	**7 years (2007-2013)**	**Wen-Li Zhou, Li Chen, et al. 周文莉,陈莉,等 (2014). Analysis of the mortality of the children under five years old in Changning District from 2007-2013 (长宁区 2007-2013 年 5 岁以下儿童死亡状况分析). hanghai Medical &**	**99,610**

							**Pharmaceutical Journal (上海医药). 35(20): 43-45**	
**C279**	**Zhu HF et al.**	**2014**	**Zhejiang**	**1 city Ningbo**	**Urban and Rural**	**6 years (2006-2011)**	**Han-Fei Zhu, Lu Liu, et al. 朱寒飞,刘璐,等 (2014). Analysis and countermeasures of deaths of children under 5 years old in Ningbo city from 2006 to 2011* (2006 至 2011 年宁波市 5 岁以下儿童死亡分析及对策). Zhejiang Clinical Medical Journal (浙江临床医学). 16(5): 768-769**	**29,196**
**C280**	**Zhu SL**	**2014**	**Yunnan**	**1 county Ludian**	**Urban and Rural**	**6 years (2008-2013)**	**Shun-Li Zhu 朱顺利 (2014). An analysis of death monitoring situation of children under 5 years old in Ludian county, China from 2008 to 2013* (鲁甸县 2008--2013 年 5 岁以下儿童死亡监测情况分析). China Health Care & Nutrition (中国保健营养（上旬刊）). (6): 3519**	**23,608**
**C281**	**Sun YQ et al.**	**2009**	**Heilongjiang**	**1 city Mishan**	**Urban and Rural**	**6 years (2003-2008)**	**Yu-Qing Sun, Dong-Lei Geng 孙玉清，耿东磊 (2009) Relavent factors of life monitoring results of children under 5 years old**	**16,623**

							in Mishan city from 2001 to 2008 (密山市 2001 年-2008 年 5 岁以下儿童生命监测结果相关因素分析). World Health Digest Medical Periodical 中外健康文摘,6(02X):194-195.	
C282	Zhan HL	2009	Yunnan	1district, 4 counties and 63 towns in Lijiang city	Urban and Rural	10 years (1999-2008)	Hui-Lian Zhan 詹会莲 (2009) Tendency analysis of death causes of pre-term births in Lijiang city from 1999 to 2008 (丽江市 1999～2008 年早产儿死亡趋势及死因分析). CJCHC 中国儿童保健杂志,17(4):495-496.	113,002
C283	Zhao H et al.	2009	Guizhou	6 counties in Guizhou city	Urban and Rural	3 years (2006-2008)	Hong Zhao, Zhen-Ju Jin 赵鸿，金真菊 (2009) Death factors of 1509 neonates (1509 例新生儿死亡因素分析). Chinese Journal of Woman and Child Health Research 中国妇幼健康研究,20(6):686-688.	97,040
C284	Zhong R et al.	2009	Guangxi	1 city Baise	Urban and Rural	10 years (1998-2007)	Run Zhong, Jian-Wei Nong, et al. 钟润，农建伟，等 (2009). Result analysis of life monitoring of children under 5 years	441,148

							**old in Baise city in recent 10 years (百色市 5 岁以下儿童生命监测 10 年结果分析). CJCHC 中国儿童保健杂志,17(2):228-229.**	
**C285**	**Chen ZB**	**2011**	**Ningxia**	**1 county Pengyang**	**Urban and Rural**	**7 years (01/01/2004-30/12/2010)**	**Zhi-Bin Chen 陈志斌 (2011) Result analysis of life monitoring of children under 5 years old in Pengyang county (彭阳县 5 岁以下儿童生命监测结果分析).Maternal & Child Health Care of China 中国妇幼保健,26(28):4364-4365.**	**25,534**
**C286**	**Wang ZW**	**2014**	**Yunnan**	**10 towns in Longling county**	**Rural**	**5 years (2007-2011)**	**Zi-Wei Wang 王子位 (2014) An analysis and countermeasures of death causes of children under 5 years old in Longling county from 2007 to 2011 (龙陵县 2007--2011 年 5 岁以下儿童死因分析及对策). China Health & Nutrition 中国保健营养（上旬刊）,(4):2283.**	**20,072**
**C287**	**Huo K et al.**	**2010**	**Henan**	**1 province Henan**	**Urban and Rural**	**5 years (2004-2009)**	**Huo K, Zhao Y, Feng H, Yao M, Savman K, Wang X, et al. Mortality rates of children aged under five**	**4,910,352**

							in Henan province, China, 2004-2008. Paediatr Perinat Epidemiol. 2010 1990-07-01;24(4):343-8.	
C288	Yi B et al.	2011	Gansu	12 counties and 4 districts in Gansu province	Urban and Rural	6 years (2004-2008)	Yi B, Wu L, Liu H, Fang W, Hu Y, Wang Y. Rural-urban differences of neonatal mortality in a poorly developed province of China. BMC PUBLIC HEALTH. 2011;11:477.	115,765

Table S7. Detailed descriptions of the parameters in all statistical models

**Predictor**	**Criterion variable**	**Relationship**	**R^2**
Predicting the proportion of all 0-4 years deaths that are due to each of the 8 most common causes for 0-4 years period (in relation to overall U5MR)			
U5MR	% preterm or low birth weight	$\ln(PB)=0.90*\ln(U5MR)-0.19*(\ln(U5MR))^2+1.80$	0.07
U5MR	% birth asphyxia	$\ln(BA)=2.31*\ln(U5MR)-0.40*(\ln(U5MR))^2-0.47$	0.20
U5MR	% congenital abnormalities	$\ln(CA)=0.79*\ln(U5MR)-0.27*(\ln(U5MR))^2+2.36$	0.35
U5MR	% accident	$\ln(ACC)=1.39*\ln(U5MR)-0.26*(\ln(U5MR))^2+0.79$	0.04
U5MR	% pneumonia	$\ln(PN)=0.05*\ln(U5MR)+0.10*(\ln(U5MR))^2+1.75$	0.43
U5MR	% SIDS	$\ln(SIDS)=2.68*\ln(U5MR)-0.56*(\ln(U5MR))^2-1.23$	0.06
U5MR	% diarrhea	$\ln(DI)=3.49*\ln(U5MR)-0.42*(\ln(U5MR))^2-4.97$	0.36
U5MR	% neonatal sepsis	$\ln(SEP)=-0.98*\ln(U5MR)+0.05*(\ln(U5MR))^2+2.48$	0.30
Predicting the proportion of all 0-4 years deaths that occur in 3 separate age-groups: neonates, post-neonatal infants and 1-4 years children (in relation to overall U5MR)			
U5MR	% in neonates	$\ln(NEO)=0.41*\ln(U5MR)-0.08*(\ln(U5MR))^2+3.56$	0.03
U5MR	% in postneonatal infants	$\ln(PINF)=-1.12*\ln(U5MR)+0.23*(\ln(U5MR))^2+4.38$	0.13
U5MR	% in 1-4 years	$\ln(1\text{-}4y)=-0.61*\ln(U5MR)+0.10*(\ln(U5MR))^2+3.75$	0.03
Predicting the proportion of all neonatal deaths that are due to each of the 4 most common causes for neonatal period (in relation to overall U5MR)			
U5MR	% preterm or low birth weight	$\ln(PB)=0.72*\ln(U5MR)-0.16*(\ln(U5MR))^2+2.54$	0.21
U5MR	% birth asphyxia	$\ln(BA)=0.53*\ln(U5MR)-0.06*(\ln(U5MR))^2+2.31$	0.19
U5MR	% congenital abnormalities	$\ln(CA)=-4.17*\ln(U5MR)+0.75*(\ln(U5MR))^2+7.72$	0.14
U5MR	% pneumonia	$\ln(PN)=-0.27*\ln(U5MR)+0.12*(\ln(U5MR))^2+2.27$	0.20
Predicting the proportion of all postneonatal infant deaths that are due to each of the 4 most common causes for postneonatal period (in relation to overall U5MR)			
U5MR	% pneumonia	$\ln(PN)=0.26*\ln(U5MR)+0.00*(\ln(U5MR))^2+2.50$	0.05
U5MR	% congenital abnormalities	$\ln(CA)=0.91*\ln(U5MR)-0.37*(\ln(U5MR))^2+3.08$	0.49
U5MR	% SIDS	$\ln(SIDS)=-5.16*\ln(U5MR)+1.00*(\ln(U5MR))^2+8.93$	0.10
U5MR	% accident	$\ln(ACC)=-0.67*\ln(U5MR)+0.10*(\ln(U5MR))^2+3.21$	0.04
Predicting the proportion of all deaths that occur in 1-4 years children that are due to each of the 4 most common causes for 1-4 years period (in relation to overall U5MR)			
U5MR	% accident	$\ln(ACC)=0.11*\ln(U5MR)-0.01*(\ln(U5MR))^2+3.53$	0.03
U5MR	% congenital abnormalities	$\ln(CA)=3.13*\ln(U5MR)-0.66*(\ln(U5MR))^2-0.99$	0.24
U5MR	% pneumonia	$\ln(PN)=-3.41*\ln(U5MR)+0.68*(\ln(U5MR))^2+6.15$	0.19
U5MR	% diarrhea	$\ln(DI)=3.35*\ln(U5MR)-0.48*(\ln(U5MR))^2-3.53$	0.46

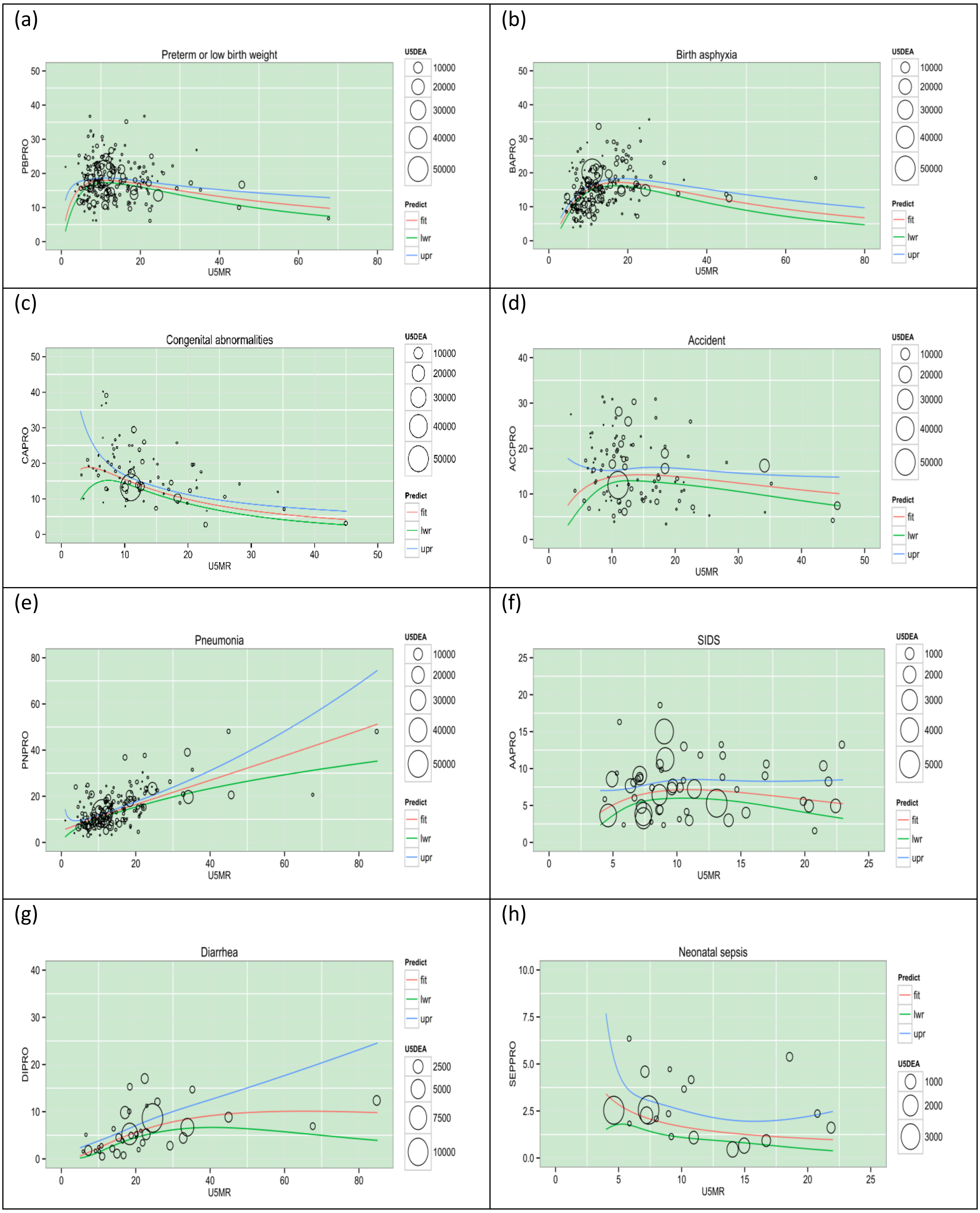

Figure S2. The relationship between U5MR and proportion of deaths in children under five years due to the most common 8 causes of death

*Note: Data points represent studies with available information and the size of the "bubbles" is proportional to the total number of child deaths observed in each study, 95% confidence interval is shown across the range of data with lower (lwr) and upper (upr) confidence

bounds.

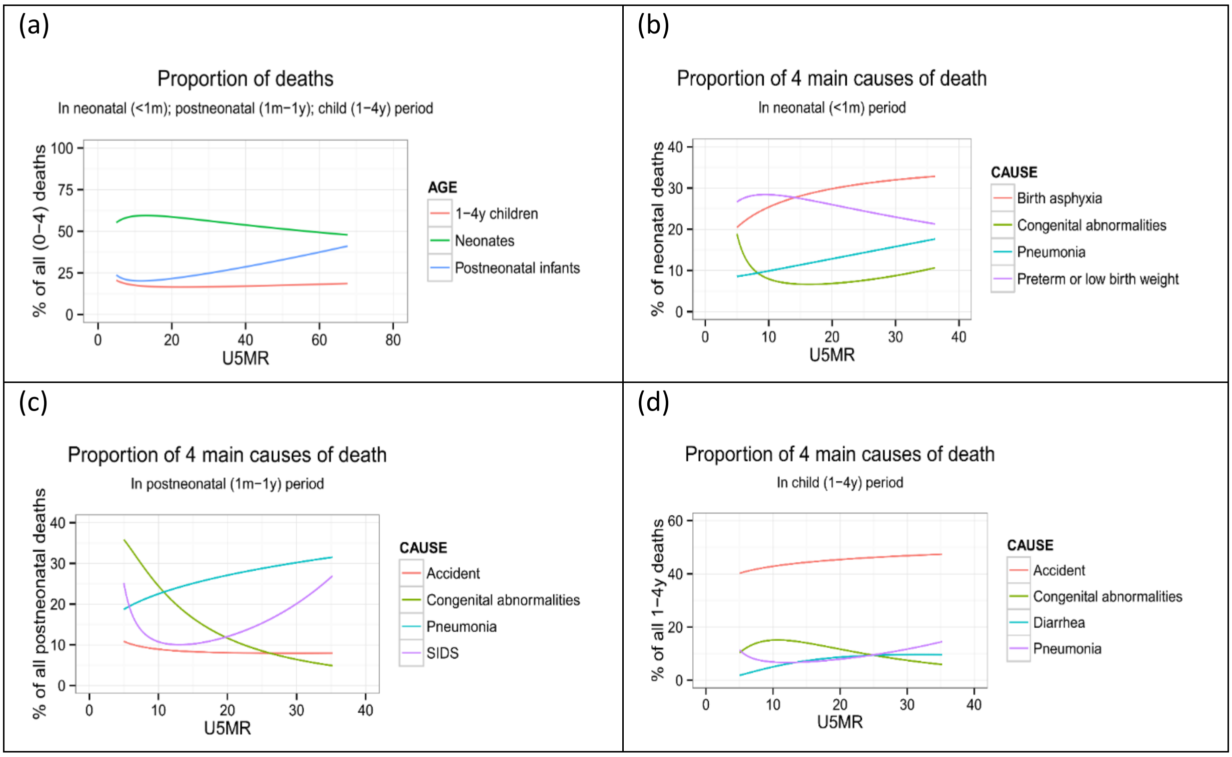

Figure S3. The relationship between U5MR and proportion of age group or deaths in children under five years

*Note: (a) relationship between U5MR and proportion of all 0-4 year deaths observed in 3 in different age groups: neonates, postneonatal infants, and 1-4 years children; (b) relationship between U5MR and proportion of neonatal deaths due to each of the 4 most common causes: Birth asphyxia, Preterm birth, Pneumonia, Congenital abnormalities; (c) relationship between U5MR and proportion of post-neonatal infant deaths due to each of the 4 most common causes: Pneumonia, SIDS, Congenital abnormalities, Accidents; (d) relationship between U5MR and proportion of deaths in children aged 1-4 years due to each of the 4 most common causes: Accidents, Congenital abnormalities, Pneumonia, Diarrhea.

REFERENCES - SUPPLEMENTARY ONLINE MATERIAL

55. NWCCW N, UNICEF. Children in China: An Atlas of Social Indicators. 2014.
56. Department of Maternal and Child Health NHaFPCoC. National Maternal and Child Health Surveillance work manual (in Chinese). Beijing, China2013.
57. Beaglehole R, Bonita R. Global public health: a new era. Oxford University Press; 2009.
58. Zeng Y, Poston Jr DL, Vlosky DA, Gu D. Healthy longevity in China: Demographic, socioeconomic, and psychological dimensions. Vol 20: Springer Science & Business Media; 2008.
59. Yu L, Lin X, Liu H, Shi J, Nong Q, Tang H, et al. Sex and Age Differences in Mortality in Southern China, 2004–2010. International journal of environmental research and public health. 2015;12(7):7886-7898.
60. Liu S, Wu X, Lopez AD, Wang L, Cai Y, Page A, et al. An integrated national mortality surveillance system for death registration and mortality surveillance, China. Bulletin of the World Health Organization. 2016;94(1):46-57.
61. Guo K, Yin P, Wang L, Ji Y, Li Q, Bishai D, et al. Propensity score weighting for addressing under-reporting in mortality surveillance: a proof-of-concept study using the nationally representative mortality data in China. Population health metrics. 2015;13(1):1-11.
62. McNicoll G. Analysing China's Population: Social Change in a New Demographic Era. WILEY-BLACKWELL 111 RIVER ST, HOBOKEN 07030-5774, NJ USA; 2015.
63. Yang G, Hu J, Rao KQ, Ma J, Rao C, Lopez AD. Mortality registration and surveillance in China: history, current situation and challenges. Popul Health Metr. 2005;3(3).
64. Rao C, Lopez AD, Yang G, Begg S, Ma J. Evaluating national cause-of-death statistics: principles and application to the case of China. Bulletin of the World Health Organization. 2005;83(8):618-625.
65. Banister J, Hill K. Mortality in China 1964–2000. Population studies. 2004;58(1):55-75.
66. Zou XN, Wan X, Dai Z, Yang GH. Epidemiological characteristics of cancer in elderly Chinese. ISRN oncology. 2012;2012.
67. Wang J-B, Jiang Y, Wei W-Q, Yang G-H, Qiao Y-L, Boffetta P. Estimation of cancer incidence and mortality attributable to smoking in China. Cancer Causes & Control. 2010;21(6):959-965.
68. Yang L, Parkin DM, Li L, Chen Y. Sources of information on the burden of cancer in China. Asian Pacific Journal of Cancer Prevention. 2003;4(1):23-30.
69. Zhao J, Jow-Ching Tu E, McMurray C, Sleigh A. Rising mortality from injury in urban China: demographic burden, underlying causes and policy implications. Bulletin of the World Health Organization. 2012;90(6):461-467.
70. World Bank Group. World development indicators 2015. World Bank Publications; 2015.
71. Zhang G, Zhao Z. Searching for the answer for Chinas fertility puzzle: data collection and data use in the last two decades. 2005.
72. Zhao Z, Chen W. China's far below-replacement fertility and its long-term impact: Comments on the preliminary results of the 2010 census. Demographic Research. 2011;25(26):819-836.
73. Basten S. Family planning restrictions and a generation of excess males: analysis of national and provincial data from the 2010 Census of China. University of Oxford,

Department of Social Policy and Intervention Oxford Centre for Population Research: Working paper. 2012;59.
74. National Bureau of Statistics of the People's Republic of China. China Statistical Yearbook 2014 (Chinese-English Edition). China Statistics Press; 2014.
75. Liang J, Mao M, Dai L, Li X, Miao L, Li Q, et al. Neonatal mortality due to preterm birth at 28–36 weeks' gestation in China, 2003–2008. Paediatric and Perinatal epidemiology. 2011;25(6):593-600.
76. Du Q, Næss Ø, Bjertness E, Yang G, Wang L, Kumar BN. Differences in reporting of maternal and child health indicators: A comparison between routine and survey data in Guizhou Province, China. International journal of women's health. 2012;4:295.
77. Gan X-L, Hao C-L, Dong X-J, Alexander S, Dramaix MW, Hu L-N, et al. Provincial Maternal Mortality Surveillance Systems in China. BioMed research international. 2014;2014.
78. Kuruvilla S, Schweitzer J, Bishai D, Chowdhury S, Caramani D, Frost L, et al. Success factors for reducing maternal and child mortality. Bulletin of the World Health Organization. 2014;92(7):533-544.
79. Feng XL, Theodoratou E, Liu L, Chan KY, Hipgrave D, Scherpbier R, et al. Social, economic, political and health system and program determinants of child mortality reduction in China between 1990 and 2006: a systematic analysis. Journal of global health. 2012;2(1).
